# Radical Rearrangement of Terminal Epoxides to Methyl Ketones via Cobalt Photocatalysis

**DOI:** 10.1021/acs.orglett.6c01341

**Published:** 2026-05-11

**Authors:** Brian Funk, Michael Yasuda, Julian G. West

**Affiliations:** Department of Chemistry, 3990 Rice University, 6100 Main St MS 602, Houston, Texas 77005, United States

## Abstract

The acid-catalyzed Meinwald rearrangement of epoxides is a powerful and economical method for generating carbonyl compounds, but regioselectivity is dominated by carbocation stability. Herein, we report a redox-neutral and highly selective cobaloxime-photocatalyzed protocol for the isomerization of terminal epoxides to methyl ketones under visible-light irradiation. This system provides complementary products to the Meinwald rearrangement under mildly basic conditions and exhibits broad functional group tolerance. Additionally, preliminary investigations suggest it proceeds through a radical mechanism.

## Graphical abstract

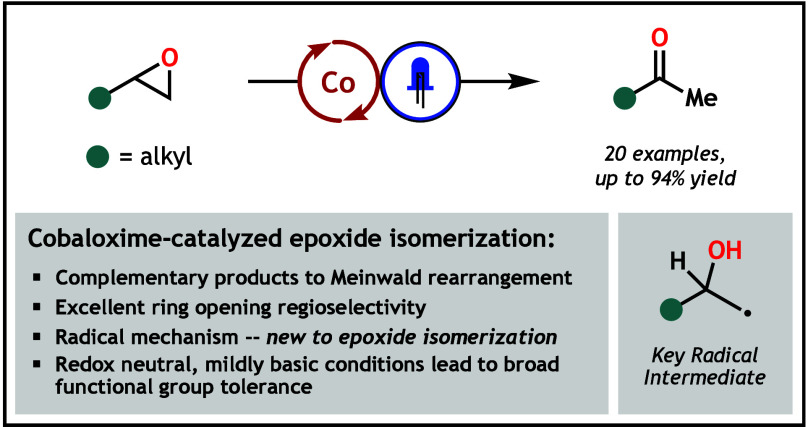

Epoxides are useful and readily available intermediates in organic synthesis due to their ease of production and versatile reactivity.^1–3^ In addition to ring opening by nucleophiles to produce alcohols, they can also undergo Lewis acid-catalyzed isomerization to carbonyl compounds, commonly known as the Meinwald rearrangement (Scheme 1A).^4^ This transformation has been extensively studied,^5–8^ and is a useful method to furnish Wacker oxidation products without the need for expensive Pd catalysts.^9,10^ However, classical Meinwald reactions often afford multiple products due to unselective, substrate-dependent ring opening and substituent migration.^11–13^ Consequently, internal epoxides typically rearrange to methyl ketones, whereas terminal epoxides generally furnish aldehydes exclusively.^14,15^

**Scheme 1:**
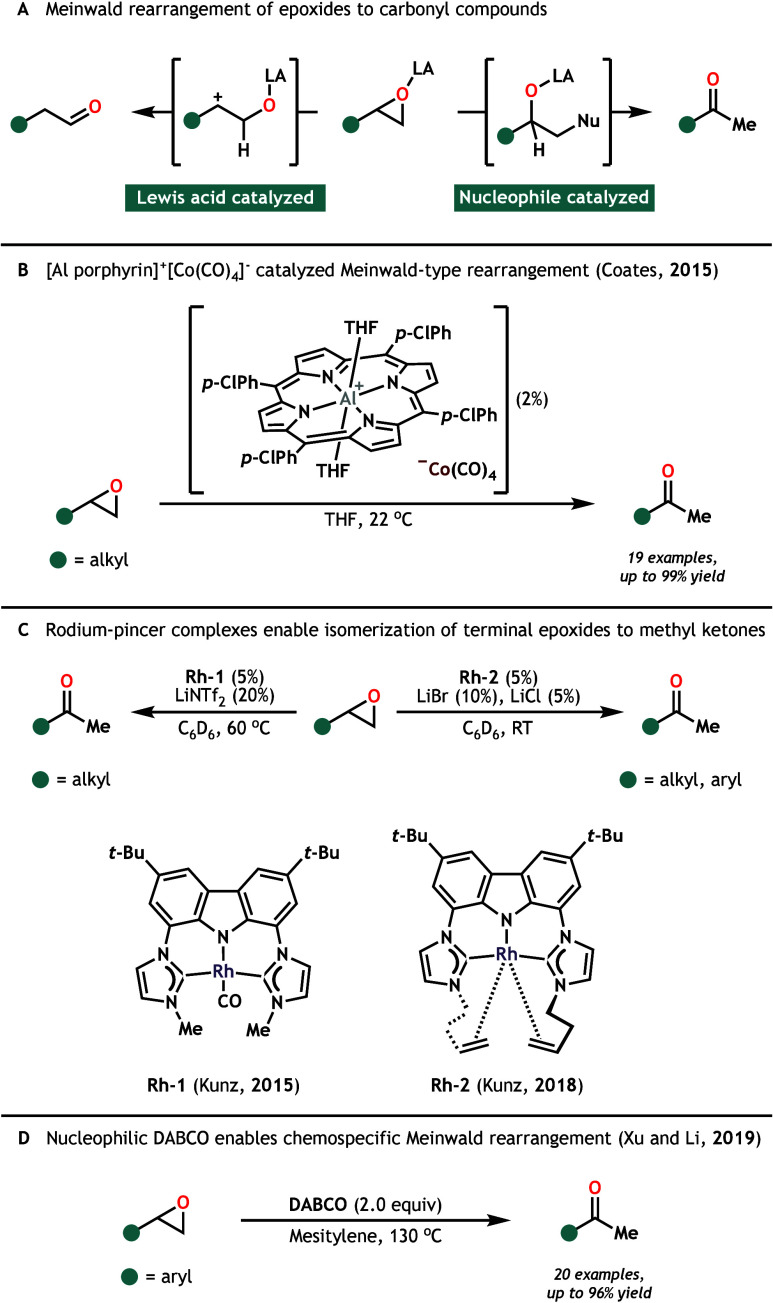
General, Metal-Catalyzed, and Base-Mediated Meinwald Rearrangement Methods

Following early reports by Eisenmann^16^ and Kagan,^17^ metal^18–20^ and amino acid^21^ catalysts have been employed for the selective rearrangement of terminal epoxides to methyl ketones, mimicking formal Meinwald rearrangements. In 2015, Coates reported the use of an air-sensitive Lewis acid-nucleophilic metal [Al porphyrin]^+^[Co­(CO)_4_]^−^ complex to effectively facilitate the isomerization of epoxides to methyl ketones at room temperature (Scheme 1B).^22^ Following this, Kunz and colleagues showed the nucleophilic Rh-pincer complex **Rh-1**, in combination with LiNTf_2_, to be a suitable alternative at elevated temperature (Scheme 1C).^23^ While these systems are selective for the rearrangement of alkyl epoxides, they lose regiocontrol when applied to aryl epoxides, affording mixtures of methyl ketones and terminal aldehydes due to competing Lewis acidic and nucleophilic pathways. In response to this, Kunz developed a modified Rh-pincer complex (**Rh-2**) with enhanced nucleophilicity, which enabled the rearrangement of both alkyl and aryl epoxides at room temperature.^24^ Later, Xu and Li developed a DABCO-mediated, chemospecific Meinwald rearrangement of aryl epoxides at high (130 °C) temperature (Scheme 1D).^25^ This thermal requirement may be reduced (80 °C) through inclusion of weak Lewis acid catalyst CuCl_2_·2H_2_O. Despite relatively strong thermal conditions and the need for superstoichiometric DABCO, their system represents a rare example of epoxide rearrangement occurring in the absence of Lewis acid additives.

Surveying these precedents, we wondered if it might be possible to recapitulate the low temperature operation of the metal-catalyzed methods of Coates^22^ and Kunz^23,24^ while also preserving the basic conditions of Xu and Li^25^ in a general and selective method for terminal epoxide rearrangement. Drawing inspiration from strategic use of cobalt catalysts to instill enantioselectivity,^26,27^ and more-pertinently regioselectivity,^28,29^ in cyclic ether ring opening, we hypothesized that achieving this goal might be possible by constructing a complementary catalytic cycle using an alkyl cobaloxime photocatalyst. Cobaloxime, or cobalt dimethylglyoximate (dmg), complexes were initially developed as structural mimics for the study of vitamin B_12_ (VB_12_) reactions.^30^ Since then, they have established their own utility within photocatalysis, facilitating a range of unprecedented synthetic transformations.^31–33^ Importantly, air stable alkyl cobaloximes can act as useful radical precursors, similarly to alkyl VB_12_ ^34^ and other organocobalt species,^35,36^ via visible light induced homolysis of a labile Co­(III)-C bond (Scheme 2A).^37–39^ The Co­(II)-alkyl radical pair resulting from this photolysis can collapse through adjacent hydrogen atom abstraction (HAA) to release an olefin and a Co-hydride equivalent.^40,41^ While this species is commonly denoted as Co­(III)-H and has similar hydrogen atom transfer (HAT) reactivity to related early metal hydrides, it has been suggested that the isomeric Co­(I) complex with protonated ligand is the operative structure.^42–44^ These Co-hydride equivalent species have a p*K*_a_ of ∼ 10 and are readily deprotonated to access nucleophilic Co­(I).^42^

**Scheme 2:**
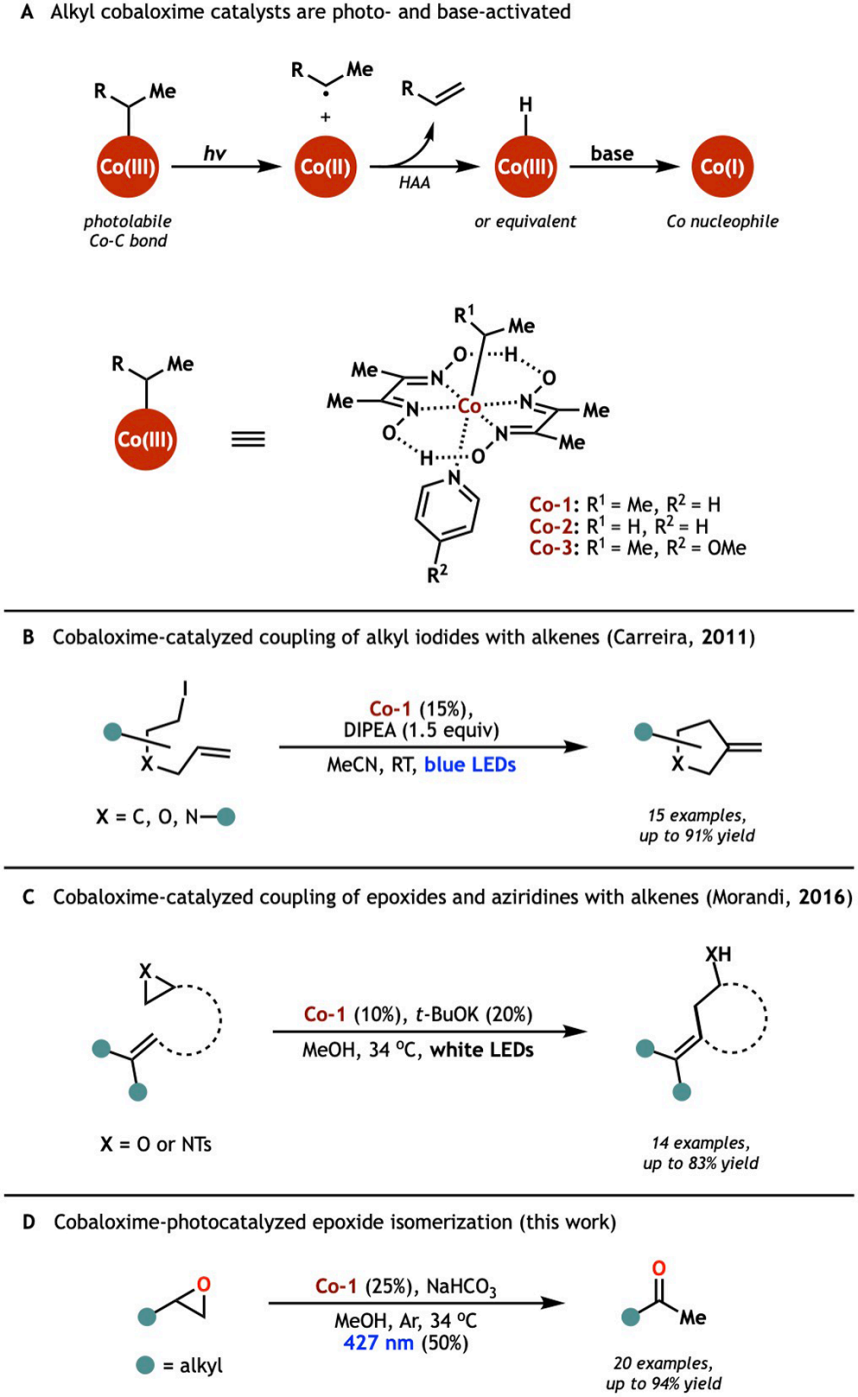
Photoactivated Alkyl Cobaloximes Catalyze a Range of Useful Transformations through Interaction with Halide and Epoxide Electrophiles

Carreira and Morandi have recently showcased this suite of reactivity in their development of photocatalytic radical cyclization methodologies using isopropyl cobaloxime precatalyst **Co-1** under basic conditions.^45,46^ Through Co­(I) nucleophilic attack on alkene-tethered iodide (Scheme 2B), epoxide, and aziridine (Scheme 2C) electrophiles and photolysis of the resultant Co­(III)–C bond, they induce radical cyclization and furnish Heck-like coupling products after HAA by Co­(II). This ability of polar cobaloxime nucleophiles to act selectively at the less-substituted position of epoxides before crossing into radical HAA reactivity suggested to us that they might enable an orthogonal approach to selective epoxide isomerization. Specifically, in the absence of competitive radical traps or cyclization, HAA from an epoxide-derived radical would form an enol that could rapidly tautomerize to a ketone.

Herein, we report our realization of this design, delivering a simple and selective cobaloxime-photocatalyzed isomerization of terminal epoxides to methyl ketones (Scheme 2D). Our system is mildly basic and exhibits excellent selectivity for ketone formation versus aldehydes, making it an advantageous complement to traditional acid-catalyzed Meinwald reactions. Further, our system exhibits a broad scope and wide functional group tolerance, and preliminary mechanistic studies are consistent with a radical mechanism new to epoxide isomerization.

We began our investigation by assessing the potential for glycidyl phenyl ether (**1A**) to undergo isomerization in the presence of alkyl cobaloxime **Co-1**. Gratifyingly, a simple system of catalytic **Co-1** (25 mol %) and NaHCO_3_ (1.0 equiv) in Argon-degassed MeOH (0.2 M) induced the isomerization of **1A** under blue light irradiation (427 nm, 50% intensity, Kessil PR160L-427), affording methyl ketone **2A** in 88% yield (Table 1, entry 1). These conditions also produced a small amount of methoxylated alcohol **3A** formed from MeOH-induced solvolytic ring opening. Control experiments revealed the necessity of both cobaloxime and light irradiation to drive this reactivity (Table 1, entries 2–3). The isomerization proceeded in the absence of explicit base, though with diminished efficiency (Table 1, entry 4). Attempts to increase or decrease **Co-1** loading invariably resulted in decreased **2A** yield (Table 1, entries 5–6). Further, modifying both the organometallic ligand (**Co-2**) and the axial base (**Co-3**) of the photocatalyst led to decreased conversion of **1A** (Table 1, entries 7–8), revealing **Co-1** to be optimal among these three. In continuing our investigation, we observed moderate (50%) irradiation intensity to be beneficial to our reaction, with increased and decreased flux resulting in lower yield (Table 1, entries 9–10). We also found heat to be ineffective in enabling the Co-mediated isomerization, instead strongly driving solvolytic ring opening (Table 1, entry 11). This side reactivity was also bolstered by stronger carbonate base (Table 1, entry 12). Finally, we were able to mitigate competitive solvolytic ring opening with less-nucleophilic *i*-PrOH solvent (Table 1, entry 13); however, this adjustment also suppressed the desired formation of **2A**, leading us to proceed with the conditions outlined in Table 1, entry 1.

**Table 1:**
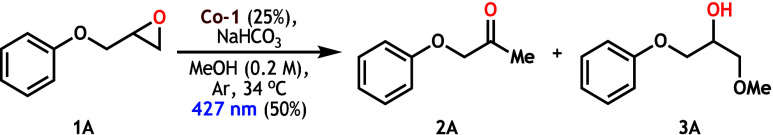

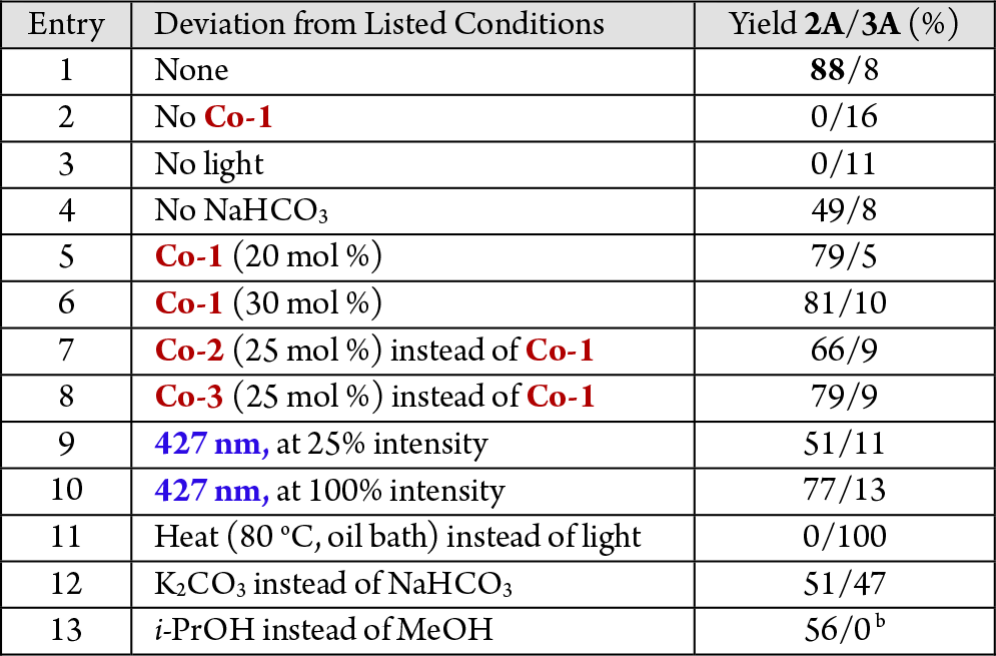
Effect of Reaction Conditions on Cobaloxime-Catalyzed Epoxide Isomerization^a^

a *Listed conditions:* **1A** (0.2 mmol, 1.0 equiv), **Co-1** (0.04 mmol, 25 mol %), NaHCO_3_ (0.2 mmol, 1.0 equiv), MeOH (degassed with Argon, 0.2 M), 427 nm irradiation (Kessil PR160L-427, 50% intensity), 22 h. Yields determined by ^1^H NMR using 1,3,5-trimethoxybenzene as an internal standard. Remaining mass balanced by unreacted starting material.

b The unformed side product refers to the isopropyl ether analogue of the methyl ether shown in **3A**.

With optimized conditions in hand, we moved to assess the scope and functional group tolerance of our mild, photocatalytic epoxide rearrangement, observing that a variety of terminal epoxides effectively isomerize to methyl ketones under these conditions (Scheme 3). In addition to accessing **2A** (94%) from our optimizing substrate, we found that simple epoxides rearranged to the corresponding methyl ketones in synthetically useful yields (**2B–D**). Further, an array of aryl glycidyl ethers indicated the tolerance of our conditions for nitrile (**1F**), amide (**1H**), and ketone (**1I**) electrophiles, forming the desired methyl ketones in >75% yields. Because of the redox neutrality of our photocatalytic system, **2J** (66%) was formed from **1J** with retention of the reductively labile benzyl ether, demonstrating the chemoselectivity of this approach. Indole-derived **2K** was able to be formed in a moderate 46% yield from epoxide **1K**; however, increased **Co-1** loading (35%) was required due to low conversion under standard conditions. To our delight, epoxides bearing silyl ether (**1M**) and Boc (**1N**) protecting groups were well-tolerated, forming methyl ketones **2M** and **2N** in 66% and 90% yields, respectively. These moieties may be susceptible to cleavage in classical acid-catalyzed Meinwald rearrangements, further demonstrating the chemoselectivity of this photocatalytic method.

**Scheme 3:**
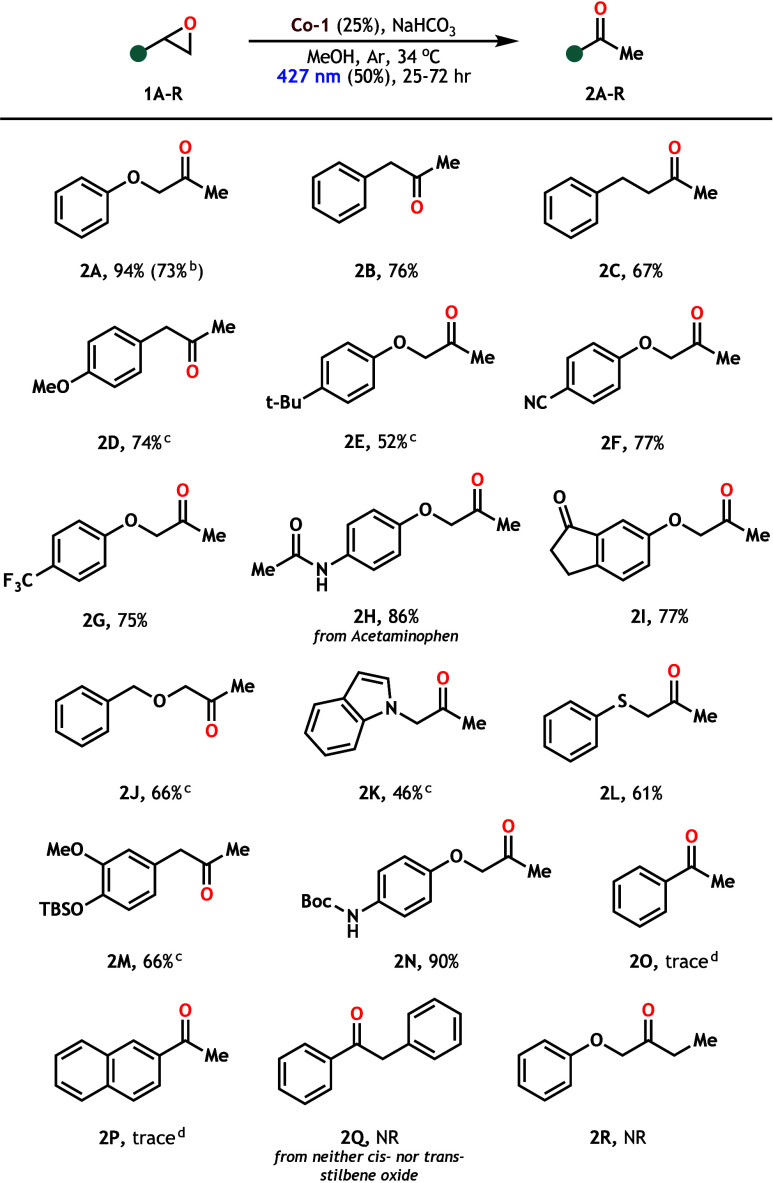
Cobaloxime-Photocatalyzed Isomerization of Terminal Epoxides to Methyl Ketones[Fn s3fn1]

a
*Reaction conditions:* epoxide (**1A-R**, 1.0 equiv), **Co-1** (25 mol %), NaHCO_3_ (1.0 equiv), MeOH (degassed with Argon, 0.2 M), 427 nm irradiation (Kessil PR160L-427, 50% intensity). Isolated yield.
b 1 mmol scale.
c Low conversion under standard conditions. Increased loading of **Co-1** (35 mol %).
d Less than 5%. Yield was determined by ^1^H NMR using 3,4,5-trichloropyridine as an internal standard.

Finally, the reactions of aryl epoxides **1O** and **1P** produced only trace amounts of the corresponding methyl ketones, with ring-opening regioselectivity favoring aldehyde formation. We wondered whether stilbene oxides (**1Q**), which structurally block the aldehyde formation pathway, may produce the desired internal ketone; however, neither *cis*- nor *trans*-stilbene oxide converted under these conditions. We hypothesize this lack of reactivity to be a steric issue, as methylated internal epoxide **1R** was also completely inert when subjected to these conditions. These results suggest that this system is uniquely selective to alkyl, monosubstituted epoxides, presenting an opportunity for chemoselectivity in cases of multiple sterically distinct epoxides.

Emboldened by the functional group tolerance and generality of our cobaloxime-photocatalyzed epoxide isomerization, we set out to evaluate whether our initial mechanistic design bore some resemblance to reality. Previous metal-catalyzed methods often propose concerted β-hydride elimination to furnish carbonyl products;^22,47^ however, the geometric improbability of this step in the context of cobaloximes^48^ provides circumstantial evidence of a different pathway at work in our system. We began our search by investigating the most fundamental element of our proposal: epoxide-derived radical generation. We first analyzed the effect radical trapping agent TEMPO had on our reaction manifold and found that stoichiometric addition of TEMPO caused a complete shutdown of isomerization reactivity (Scheme 4A). While this result is consistent with a radical mechanism inhibited by TEMPO, we are cognizant that these results must be interpreted with care.^49^ Further, this observation does not discern whether the trap is inhibiting catalyst photoactivation, presumed radical generation after Co­(I) epoxide ring opening, or both. Consequently, we subjected radical clock substrate **1S** to our standard conditions and observed competitive formation of cyclized alcohol **4S** (see Supporting Information, p. 18–21 for more details) in addition to expected methyl ketone **2S** (Scheme 4B). The formation of **4S** is highly indicative of radical generation at the terminal site of the epoxide upon ring opening.

**Scheme 4:**
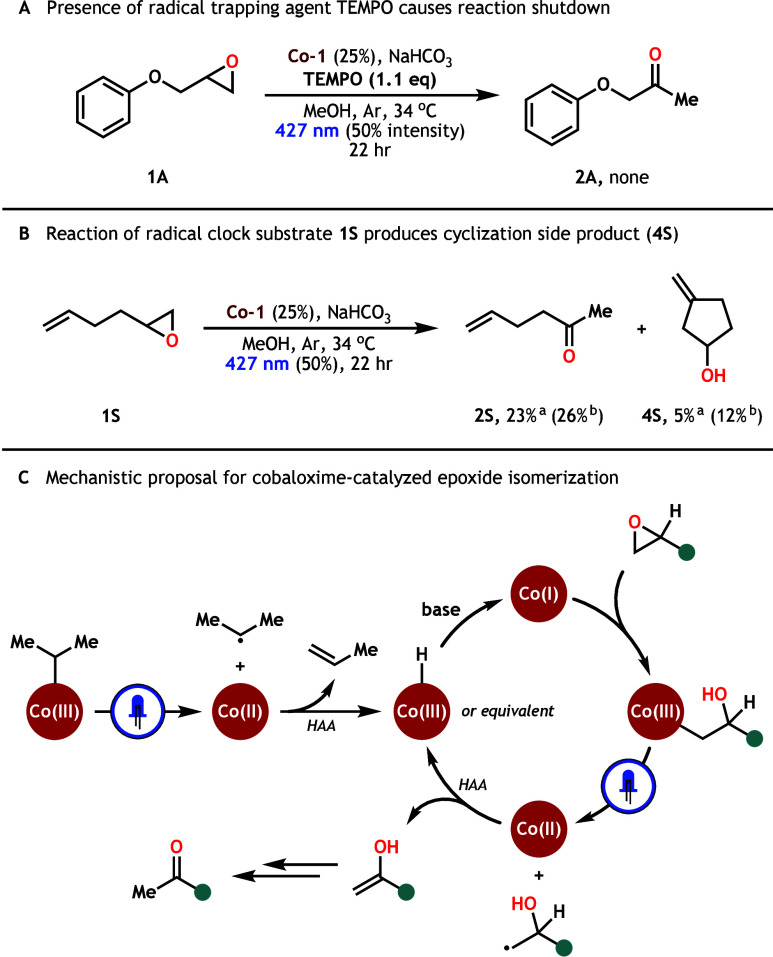
Mechanistic Insights of Epoxide Isomerization via Cobaloxime Photocatalysis

a Yield was determined by ^1^H NMR using 3,4,5-trichloropyridine as an internal standard.
b Increased loading of **Co-1** (35 mol %). Yield was determined by ^1^H NMR using 3,4,5-trichloropyridine as an internal standard.

We envisioned this radical could be quenched via Co­(II) abstraction of the adjacent hydrogen atom to form an enol intermediate. Enzymatic VB_12_ facilitates an analogous reactive pathway in the CarH photoreceptor of bacterial *T. thermophilus* and *M. xanthus* cells.^50,51^ Our lab recently leveraged this radical trapping capability to enable the olefination of alkyl electrophiles via VB_12_ photocatalysis.^52^ Furthermore, cobaloximes have been repeatedly shown to engage in HAA from radical species and the process, which releases an olefin and a cobalt hydride, is well-established throughout the literature.^46,53–56^

Taking these ideas in aggregate, we propose the following mechanism for our cobaloxime-photocatalyzed epoxide rearrangement (Scheme 4C). First, **Co-1** undergoes photolysis to release a Co­(II) metalloradical and isopropyl radical pair, which collapses through HAA to produce propene and a Co-hydride equivalent.^42–44^ Deprotonation of this species under mildly basic conditions gives rise to Co­(I) and, after nucleophilic attack, generates a Co­(III)-alkyl intermediate derived from the epoxide substrate. Photolysis of *this* alkyl cobaloxime affords a new Co­(II)-alkyl radical pair which is quenched by adjacent HAA to produce the enol and regenerate the Co-hydride equivalent, completing the catalytic cycle. Finally, the enol tautomerizes to furnish the methyl ketone product.

In summary, we have developed an epoxide isomerization protocol photocatalyzed by alkyl cobaloxime **Co-1**. This simple system presents a new method for producing methyl ketones from alkyl monosubstituted epoxides under photochemical conditions that complements classical Meinwald rearrangements. Our system is both mildly basic and redox-neutral, tolerates electrophilic and acid-labile functionalities, and exhibits excellent selectivity for methyl ketone formation due to the steric demand of the **Co-1** nucleophile, making it a useful alternative to traditionally acid- and metal-catalyzed epoxide rearrangements. Furthermore, preliminary studies are consistent with our proposed mechanism, supporting Co­(I) epoxide ring opening and photolytic radical generation, followed by Co­(II)-mediated hydrogen atom abstraction. To the best of our knowledge, the development of this method marks the first example of a catalytic epoxide rearrangement occurring via a radical mechanism, and our laboratory is actively investigating new applications of this versatile reactivity.

## Supplementary Material



## Data Availability

The data underlying this study are available in the published article and its Supporting Information.

## Notes

Experimental methods and procedures, characterization details and NMR spectra of featured compounds (PDF)
