# Supplementary material for: Radical Rearrangement of Terminal Epoxides to Methyl Ketones via Cobalt Photocatalysis
Source: Org Lett. 2026 May 11;28(20):6281–6. doi: 10.1021/acs.orglett.6c01341 (PMC13200259; doi:10.1021/acs.orglett.6c01341)
Supplement: Supplementary file 1 [file ol6c01341_si_001.pdf]

Supporting Information

Radical Rearrangement of Terminal Epoxides to Methyl Ketones via Cobalt Photocatalysis

Brian Funk, Michael Yasuda, and Julian G. West*

Department of Chemistry, Rice University, 6100 Main St MS 602, Houston, Texas 77005

*Email: jgwest@rice.edu

Table of Contents

Supplemental Methods 3
General Optimization 4
Reaction Setup 5
Synthesis of Cobaloxime Catalysts
General Procedure 1 6
Synthesis of Epoxide Substrates
General Procedure 2 8
General Procedure 3 9
Synthesis of 1-(oxiran-2-ylmethyl)-1H-indole (**1K**) 11
Synthesis of tert-butyl(2-methoxy-4-(oxiran-2-ylmethyl)phenoxy)dimethylsilane (**1M**) 11
Synthesis of 2-methyl-3-(phenoxymethyl)oxirane (**1R**) 12
Cobaloxime-photocatalyzed Epoxide Isomerization
General Procedure 4 14
Mechanistic Investigations
TEMPO Radical Trap Experiment 18
Radical Clock Experiment 18
Synthesis of Radical Clock Dopant 19
Proposed Mechanism 22
References 23
^{1}H, ^{13}C, and ^{19}F NMR Spectra 25

Supplemental Methods

All reactions were carried out at ambient temperature (21-25 °C), in wet solvent, and with magnetic stirring unless otherwise specified. Reactions were monitored by thin-layer chromatography (TLC) and/or ^1H NMR as appropriate. All commercially available reagents and solvents were purchased from common suppliers and used as received without any further purification.

Silica gel column chromatography was carried out using Silicycle P60 silica gel (40-63 µm, 230-400 mesh). Analytical thin-layer chromatography (TLC) was performed with Silicycle F-254 glass plates (250 µm). Analytical TLC was analyzed using short-wave UV light (254 nm) as a visualizing agent as well as $KMnO_4$ and Phosphomolybdic acid (PMA) as heat-developing stains prepared in our laboratory. Preparative TLC was conducted using Uniplate UV254 glass plates (1000 µm).

High resolution mass spectrometry (HRMS) analysis was performed on an Agilent 1290 Infinity II Binary HPLC system interfaced with an Agilent 6545XT Q-TOF mass spectrometer equipped with an Agilent Jet Stream electrospray ionization (ESI) source. Samples were diluted in LCMS grade methanol or acetonitrile to a final concentration of approximately 10 ng/µL. Flow injection analysis (FIA) was performed by injecting 1-5 µL of the sample directly into the ESI source via a flow injection loop. The flow rate was 0.4 mL/min. A mixture of 30% acetonitrile/water with 0.1% formic acid was employed as mobile phase.

^1H, ^{13}C, and ^{19}F NMR spectra were acquired on a Bruker 600 Avance Spectrometer operating at 600 MHz for ^1H NMR, 151 MHz for ^{13}C NMR, and 594 MHz for ^{19}F NMR. The spectra were calibrated based on residual non-deuterated solvent peaks ($CDCl_3$, δ 7.26 ppm in ^1H NMR and δ 77.0 ppm in ^{13}C NMR). For quantitative analyses (i.e. yields by ^1H NMR), delay time (D1) was adjusted to 10 seconds. Peak multiplicities are abbreviated as follows: s = singlet, d = doublet, t = triplet, q = quartet, p = pentet, m = multiplet, bs = broad singlet, dd = doublet of doublets, dt = doublet of triplets, td = triplet of doublets, ddt = doublet of doublet of triplets, dqd = doublet of quartet of doublets.

General Optimization

Table S1. Cobaloxime-photocatalyzed epoxide isomerization, expanded optimization

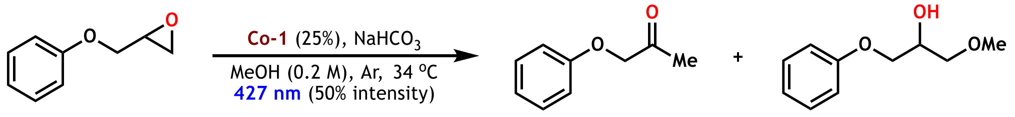

Entry	Deviation from Optimized Conditions	Conversion 1A (%)	Yield 2A (%)	Yield 3A (%)
1	None	96	**88**	8
2	No **Co-1**	16	--	16
3	No light	12	--	11
4	No $NaHCO_3$	58	49	8
5	**Co-1** (10 mol %)	44	36	6
6	**Co-1** (20 mol %)	85	79	5
7	**Co-1** (30 mol %)	91	81	10
8	**Co-2** instead of **Co-1**	76	66	9
9	**Co-3** instead of **Co-1**	90	79	9
10	427 nm, at 25% intensity	62	51	11
11	427 nm, at 100% intensity	90	77	13
12	456 nm, at 50% intensity	76	64	11
13	Heat (80 °C, oil bath) instead of light	100	--	100
14	$NaHCO_3$ (2.0 equiv)	82	73	9
15	KOtBu instead of $NaHCO_3$	100	56	42
16	DIPEA instead of $NaHCO_3$	92	84	8
17	K_2CO_3 instead of $NaHCO_3$	100	51	47
18	i-PrOH instead of MeOH	57	56	--[b]
19	MeCN or THF instead of MeOH	--	**--**	--
20	MeOH (0.13 M)	95	84	9
21	MeOH (0.1 M)	92	**86**	5
22	N_2 purge instead of Ar purge	77	63	14

Optimized conditions: **Co-1** (0.04 mmol, 25 mol %) and $NaHCO_3$ (0.2 mmol, 1.0 equiv) were added to an 8-mL septa-capped vial equipped with a magnetic stir bar. The vial was sparged with Argon for 5 minutes before **1A** (0.2 mmol, 1.0 equiv) was added and the mixture was dissolved in Argon-degassed MeOH (0.2 M). The septum was sealed with grease and covered with tape, and the reaction was stirred and irradiated with 427-nm LED (Kessil®, 50% intensity) for 22 h. Yields determined by ^1H NMR using 1,3,5-trimethoxybenzene as an internal standard. [b] In this entry, the unformed side product refers to the isopropyl ether analogous to the methyl ether shown in **3A**.

Reaction Set Up

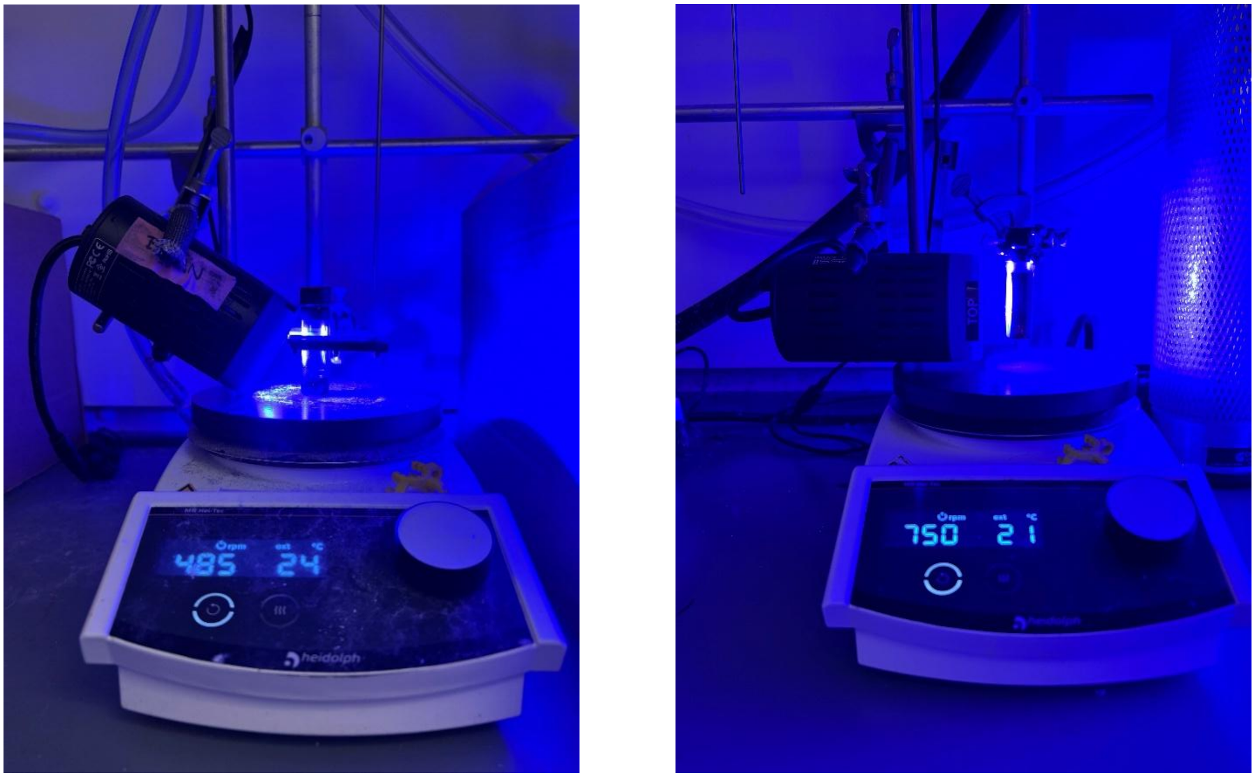

Figure S1. (Left) Cobaloxime-photocatalyzed epoxide isomerization reaction setup/apparatus (0.2-mmol scale). (Right) 1-mmol scale-up reaction.

Synthesis of Cobaloxime Catalysts

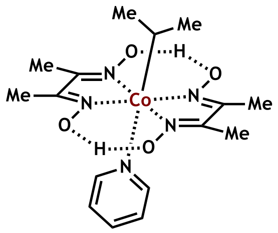

Co-1
[Co(dmgH)$_2$(py)i-Pr]

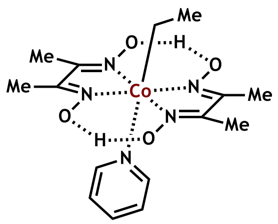

Co-2
[Co(dmgH)$_2$(py)Et]

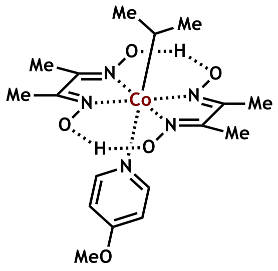

Co-3
[Co(dmgH)$_2$(PMP)i-Pr]

General Procedure 1 (adapted from Carreira and co-workers[S1])

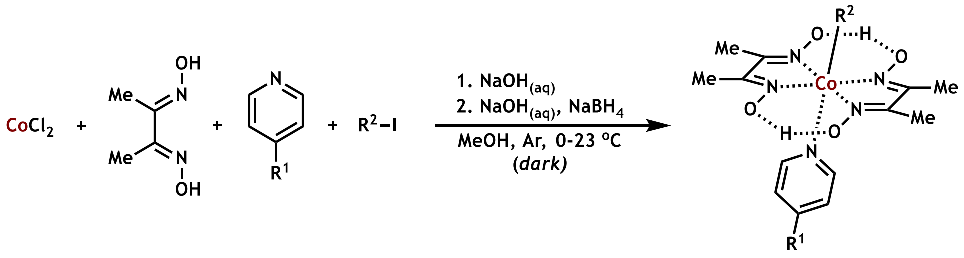

In a 250-mL round bottom flask equipped with magnetic stir bar, $CoCl_2$ (1.0 equiv) and dimethylglyoxime (2.0 equiv) were dissolved in MeOH (degassed with Argon, 0.16 M) and stirred for 10 min at room temperature. **Pyridine** (*or derivative*, 1.01 equiv) and NaOH (2.5 M in degassed H_2O, 2.0 equiv) were added sequentially and the solution was stirred for 10 min before cooling to 0 °C (ice bath). Additional NaOH (2.5 M in degassed H_2O, 1.0 equiv) was added, and $NaBH_4$ (2.0 equiv) was added portionwise at 0 °C. Finally, **alkyl iodide** (1.2 equiv) was added dropwise, the solution was allowed to warm to room temperature, and stirred for 2 hr. Then, the solution was diluted with H_2O, and extracted with DCM (3x). The combined organic layers were dried over Na_2SO_4, filtered and concentrated. To isolate the cobaloxime catalyst, it was then recrystallized (MeOH, 70 °C), filtered, washed with ice-cold Et_2O (3x) and ice-cold MeOH (2x), then dried on high vacuum.

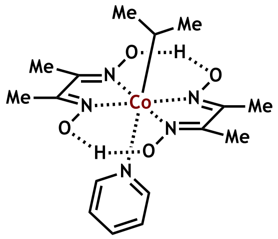

Co-1

General Procedure 1 was conducted (10-mmol scale) with pyridine (0.82 mL, 1.01 equiv) and 2-iodopropane (1.2 mL, 1.2 equiv). Upon recrystallization, **Co-1** (1.927 g, 47%) was collected as a red-orange solid. ^{1}H NMR (600 MHz, $CDCl_3$): δ 8.59 (d, J = 4.9 Hz, 2H), 7.70-7.66 (m, 1H), 7.30-7.26 (m, 2H), 2.13 (s, 12H), 1.97-1.91 (m, 1H), 0.47 (d, J = 6.8 Hz, 6H); ^{13}C NMR (151 MHz, $CDCl_3$): δ 150.0, 149.3, 137.2, 125.1, 26.3, 12.0. The spectra are consistent with those reported in literature.[S1]

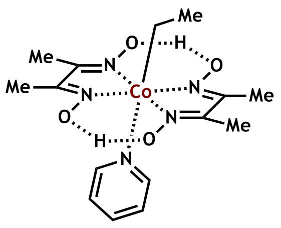

Co-2

General Procedure 1 was conducted (5-mmol scale) with pyridine (0.41 mL, 1.01 equiv) and iodoethane (0.4 mL, 1.0 equiv). Upon recrystallization, **Co-2** (667.8 mg, 34%) was collected as an orange powder. ^{1}H NMR (600 MHz, $CDCl_3$): δ 8.59 (d, J = 5.1 Hz, 2H), 7.70 (t, J = 7.6 Hz, 1H), 7.30 (t, J = 6.3 Hz, 2H), 2.12 (s, 12H), 1.72 (q, J = 7.7 Hz, 2H), 0.35 (t, J = 7.7 Hz, 3H); ^{13}C NMR (151 MHz, $CDCl_3$): δ 150.2, 149.1, 137.5, 125.3, 16.0, 12.1 (Co-**C** not observed between -20 – 220 ppm). The spectra are consistent with reports in literature.[S2]

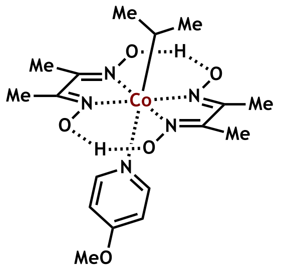

Co-3

General Procedure 1 was conducted (5-mmol scale) with *p*-methoxypyridine (PMP, 0.52 mL, 1.01 equiv) and 2-iodopropane (0.51 mL, 1.02 equiv). Upon recrystallization, **Co-3** (375.1 mg, 17%) was collected as a red crystalline solid. ^{1}H NMR (600 MHz, $CDCl_3$): δ 8.38-8.36 (m, 2H), 6.77-6.75 (m, 2H), 3.81 (s, 3H), 2.13 (s, 12H), 1.92-1.84 (m, 1H), 0.47 (d, J = 6.8 Hz, 6H); ^{13}C NMR (151 MHz, $CDCl_3$): δ 166.2, 151.0, 149.1, 111.1, 55.4, 26.2, 12.0 (Co-**C** not observed between -20 – 220 ppm); MS (ESI) calculated for $C_{14}H_{21}CoN_5O_5$ [M-C_3H_7]: 398.0875, observed 398.0867.

Synthesis of Epoxide Substrates
General Procedure 2

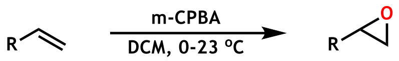

In a round-bottom flask equipped with magnetic stir bar, alkene (4.2-9.0 mmol, 1.0 equiv) was dissolved in DCM (0.33 M) and the solution was brought to 0 °C (ice bath). Then, m-CPBA (≤77% purity, 1.5 equiv) was added in three, 0.5-equivalence portions, and the solution was allowed to warm to room temperature and stir overnight. Upon completion, the reaction was diluted with DCM and Na_2SO_3 (10% in H_2O) and stirred vigorously for 30 min. The aqueous layer was removed, and the organic phase was washed with Na_2SO_3 (10% in H_2O), sat. $NaHCO_3$ (3x), and brine. The DCM layer was then dried over Na_2SO_4, filtered, and concentrated. Chromatography purification afforded the epoxide substrates listed below. *Note:* This procedure was performed on varying scales, ranging from 4.2 mmol to 9.0 mmol, and the molar quantity of the alkene is specified in each substrate characterization.

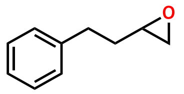

1C

4-phenyl-1-butene (787.8 mg, 5.962 mmol) was reacted for 19 hr according to **General Procedure 2**. Purification by silica gel column chromatography (20% ethyl acetate in hexane) afforded **1C** (680.5 mg, 76%) as a colorless oil. ^1H NMR (600 MHz, $CDCl_3$): δ 7.31-7.28 (m, 2H), 7.23-7.18 (m, 3H), 2.98-2.94 (m, 1H), 2.86-2.80 (m, 1H), 2.79-2.72 (m, 2H), 2.48 (dd, J = 4.8, 2.7 Hz, 1H), 1.92-1.80 (m, 2H); ^{13}C NMR (151 MHz, $CDCl_3$): δ 141.3, 128.5, 128.4, 126.0, 51.8, 47.3, 34.3, 32.3. The spectra are consistent with those reported in literature.[S3]

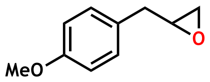

1D

Estragole (623.0 mg, 4.204 mmol) was reacted for 24 hr according to **General Procedure 2**. Purification by silica gel column chromatography (20% ethyl acetate in hexane) afforded **1D** (576.5 mg, 83%) as a colorless oil. ^1H NMR (600 MHz, $CDCl_3$) δ 7.17 (d, *J* = 8.4 Hz, 2H), 6.86 (d, J = 8.6 Hz, 2H), 3.80 (s, 3H), 3.14-3.10 (m, 1H), 2.87 (dd, J = 14.6, 5.6 Hz, 1H), 2.80-2.74 (m, 2H), 2.53 (dd, J = 5.0, 2.6 Hz, 1H); ^{13}C NMR (151 MHz, $CDCl_3$) δ 158.4, 130.0, 129.1, 113.9, 55.3, 52.7, 46.8, 37.8. The spectra are consistent with those reported in literature.[S3]

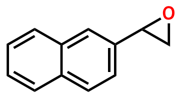

1P

2-vinylnaphthalene (758.9 mg, 4.921 mmol) was reacted for 23 hr according to **General Procedure 2**. Purification by silica gel column chromatography (5-15% ethyl ether in hexane) afforded **1P** (233.7 mg, 28%) as a white solid. ^1H NMR (600 MHz, $CDCl_3$): δ 7.85-7.80 (m, 4H), 7.51-7.46 (m, 2H), 7.33 (dd, *J* = 8.5, 1.7 Hz, 1H), 4.04 (dd, *J* = 3.9, 2.8 Hz, 1H), 3.23 (dd, *J* = 5.3, 4.2 Hz, 1H), 2.92 (dd, *J* = 5.4, 2.6 Hz, 1H); ^{13}C NMR (151 MHz, $CDCl_3$): δ 135.2, 133.4, 133.3, 128.5, 127.9, 127.9, 126.5, 126.2, 125.3, 122.8, 52.8, 51.5. The spectra are consistent with those found in literature.[S4]

General Procedure 3

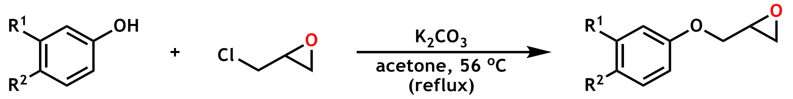

In a 150-mL round-bottom flask equipped with magnetic stir bar, the phenol derivative (6.0-13.3 mmol, 1.0 equiv) was dissolved in acetone (0.4 M) and stirred. K_2CO_3 (3.0 equiv) and epichlorohydrin (8.0 equiv) were added consecutively. The resulting mixture was heated to a gentle reflux (56 °C) and stirred overnight. The reaction was then cooled to room temperature, and the solids were filtered off. The remaining filtrate was concentrated *en vacuo*, immersed in ethyl acetate, and washed with H_2O and 1M NaOH. The organic layer was dried over Na_2SO_4, filtered, and concentrated to afford the crude product. Chromatography purification afforded the glycidyl ethers displayed below. *Note:* This procedure was performed on varying scales, ranging from 6.0 mmol to 13.3 mmol, and the molar quantity of the phenol derivative is specified in each substrate characterization.

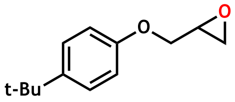

1E

4-tert-butylphenol (2.002 g, 13.327 mmol) was reacted for 24 hr according to **General Procedure 3.** Purification by silica gel column chromatography (10% ethyl acetate in hexane) afforded **1E** (979.8 mg, 36%) as a yellow oil. ^{1}H NMR (600 MHz, $CDCl_3$): δ 7.35-7.31 (m, 2H), 6.91-6.87 (m, 2H), 4.21 (dd, J = 11.0, 3.2 Hz, 1H), 3.99 (dd, J = 10.9, 5.6 Hz, 1H), 3.39-3.35 (m, 1H), 2.92 (t, J = 4.7 Hz, 1H), 2.78 (dd, J = 5.1, 2.8 Hz, 1H), 1.32 (s, 9H); ^{13}C NMR (151 MHz, $CDCl_3$): δ 156.2, 144.0, 126.3, 114.1, 68.8, 50.2, 44.8, 34.1, 31.5. The spectra are consistent with those reported in literature.[S3]

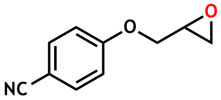

1F

4-cyanophenol (953.3 mg, 8.003 mmol) was reacted for 24 hr according to **General Procedure 3**. Purification by silica gel column chromatography (20-40% ethyl acetate in hexane) afforded **1F** (1,090.0 mg, 77%) as a white solid. ^{1}H NMR (600 MHz, $CDCl_3$): δ 7.59 (d, *J* = 9.0 Hz, 2H), 6.98 (d, *J* = 8.9 Hz, 2H), 4.32 (dd, *J* = 11.0, 2.8 Hz, 1H), 3.96 (dd, *J* = 11.0, 5.5 Hz, 1H), 3.39-3.34 (m, 1H), 2.93 (t, *J* = 4.5 Hz, 1H), 2.77 (dd, *J* = 4.8, 2.6 Hz, 1H); ^{13}C NMR (151 MHz, $CDCl_3$): δ 161.8, 134.2, 119.2, 115.5, 104.7, 69.2, 49.9, 44.6. The spectra are consistent with those found in literature.[S3]

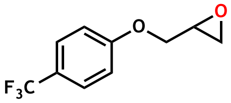

1G

4-(trifluoromethyl)phenol (1,292.4 mg, 7.972 mmol) was reacted for 25 hr according to **General Procedure 3**. Purification by silica gel column chromatography (20-40% ethyl acetate in hexane) afforded **1G** (1.224 g, 70%) as a pale yellow liquid. ^{1}H NMR (600 MHz, $CDCl_3$): δ 7.55 (d, *J* = 8.4 Hz, 2H), 6.99 (d, *J* = 8.7 Hz, 2H), 4.30 (dd, *J* = 11.0, 2.9 Hz, 1H), 3.98 (dd, *J* = 11.0, 5.8 Hz, 1H), 3.39-3.36 (m, 1H), 2.93 (t, *J* = 4.6 Hz, 1H), 2.77 (dd, *J* = 4.9, 2.6 Hz, 1H); ^{13}C NMR (151 MHz, $CDCl_3$): δ 161.0, 127.1 (q_{C-F}, *J* = 3.9 Hz), 124.5 (q_{C-F}, *J* = 272.0 Hz), 123.6 (q_{C-F}, *J* = 32.7 Hz), 114.7, 69.0, 50.1, 44.7; ^{19}F NMR (594 MHz, $CDCl_3$): δ -61.6. The spectra are consistent with those found in literature.[S3]

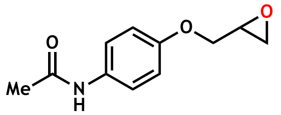

1H

from Acetaminophen

Acetaminophen (1,210.5 mg, 8.008 mmol) was reacted for 24 hr according to **General Procedure 3**. Purification by silica gel column chromatography (100% ethyl acetate) afforded **1H** (770.0 mg, 46%) as a white solid. ^{1}H NMR (600 MHz, $CDCl_3$): δ 7.44 (bs, 1H), 7.38 (d, *J* = 9.0 Hz, 2H), 6.85 (d, *J* = 9.0 Hz, 2H), 4.20 (dd, *J* = 11.0, 3.1 Hz, 1H), 3.90 (dd, *J* = 11.0, 5.7 Hz, 1H), 3.36-3.32 (m, 1H), 2.90 (dd, *J* = 4.8, 4.3 Hz, 1H), 2.75 (dd, *J* = 4.9, 2.7 Hz, 1H), 2.13 (s, 3H); ^{13}C NMR (151 MHz, $CDCl_3$): δ 168.5, 155.4, 131.7, 122.0, 115.1, 69.2, 50.3, 44.8, 24.5. The spectra are consistent with those found in literature.[S4]

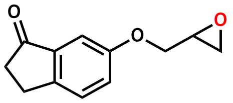

1I

6-hydroxy-1-indanone (1,185.3 mg, 8.000 mmol) was reacted for 25 hr according to **General Procedure 3**. Purification by silica gel column chromatography (40-60% ethyl acetate in hexane) afforded **1I** (379.5 mg, 24%) as a white solid. ^{1}H NMR (600 MHz, $CDCl_3$): δ 7.38 (d, *J* = 8.4 Hz, 1H), 7.23 (dd, *J* = 8.4, 2.6 Hz, 1H), 7.17 (d, *J* = 2.5 Hz, 1H), 4.30 (dd, *J* = 11.0, 2.8 Hz, 1H), 3.93 (dd, *J* = 11.0, 6.0 Hz, 1H), 3.39-3.35 (m, 1H), 3.09-3.04 (m, 1H), 2.91 (t, *J* = 4.5 Hz, 1H), 2.76 (dd, *J* = 4.9, 2.7 Hz, 1H), 2.73-2.69 (m, 1H); ^{13}C NMR (151 MHz, $CDCl_3$): δ 207.1, 158.3, 148.6, 138.3, 127.7, 124.6, 105.8, 69.2, 50.1, 44.7, 37.1, 25.3; MS (ESI) calculated for $C_{12}H_{13}O_3$ $[M+H]^+$: 205.0865, observed 205.0855.

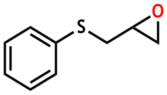

1L

Thiophenol (830 µL, 8.083 mmol) was reacted for 27 hr according to **General Procedure 3**. Purification by silica gel column chromatography (10% ethyl acetate in hexane) afforded **1L** (879.6 mg, 66%) as a pale yellow oil. ^{1}H NMR (600 MHz, $CDCl_3$) δ 7.45–7.41 (m, 2H), 7.30 (t, *J* = 7.6 Hz, 2H), 7.23 (t, *J* = 7.4 Hz, 1H), 3.20-3.15 (m, 2H), 2.94 (dd, *J* = 15.3, 7.3 Hz, 1H), 2.78 (t, *J* = 4.3 Hz, 1H), 2.52 (dd, *J* = 4.8, 2.4 Hz, 1H); ^{13}C NMR (151 MHz, $CDCl_3$) δ 135.3, 130.4, 129.1, 126.8, 51.0, 47.4, 36.7. The spectra are consistent with those reported in literature.[S3]

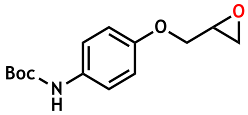

1N

4-N-tert-butoxycarbonylaminophenol (1.676 g, 8.009 mmol) was reacted for 40 hr according to **General Procedure 3**. Purification by silica gel column chromatography (20-40% ethyl acetate in hexane) afforded **1N** (1.943 g, 91%) as a white powder. ^{1}H NMR (600 MHz, $CDCl_3$) δ 7.28-7.24 (m, 2H), 6.85 (d, *J* = 8.9 Hz, 2H), 6.37 (bs, 1H), 4.18 (dd, *J* = 11.0, 3.1 Hz, 1H), 3.92 (dd, *J* = 11.0, 5.6 Hz, 1H), 3.35-3.31 (m, 1H), 2.89 (t, *J* = 4.5 Hz, 1H), 2.74 (dd, *J* = 4.8, 2.6 Hz, 1H), 1.50 (s, 9H); ^{13}C NMR (151 MHz, $CDCl_3$) δ 154.5, 153.1, 132.0, 120.4, 115.1, 69.1, 50.2, 44.7, 28.4. The spectra are consistent with those reported in literature.[S5]

Synthesis of 1-(oxiran-2-ylmethyl)-1H-indole (1K)

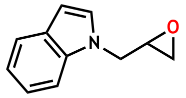

1K

Indole (703.1 mg, 6.002 mmol) was reacted for 24 hours according to **General Procedure 3.** Purification by silica gel column chromatography (15% ethyl acetate in hexane) afforded **1K** (638.8 mg, 61%) as a yellow oil. ^{1}H NMR (600 MHz, $CDCl_3$) δ 7.67 (d, J = 7.9 Hz 1H), 7.42 (d, J = 8.3 Hz, 1H), 7.26 (t, J = 8.2 Hz, 1H), 7.17-7.14 (m, 2H), 6.56 (d, J = 3.2 Hz, 1H), 4.46 (d, J = 15.3 Hz, 1H), 4.23 (d, J = 15.3 Hz, 1H), 3.33-3.30 (m, 1H), 2.84 (d, J = 4.8 Hz, 1H), 2.49 (dd, J = 4.8, 2.6 Hz, 1H); ^{13}C NMR (151 MHz, $CDCl_3$) δ 136.3, 128.6, 128.2, 121.8, 121.0, 119.6, 109.3, 101.9, 50.9, 47.8, 45.3. The spectra are consistent with those reported in literature.[S3]

Synthesis of tert-butyl(2-methoxy-4-(oxiran-2-ylmethyl)phenoxy)dimethylsilane (1M)

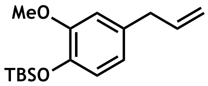

S1

In a 100-mL round bottom flask equipped with magnetic stir bar, eugenol (1.55 mL, 10.006 mmol) and 1H-imidazole (1.362 g, 20.006 mmol) were dissolved in DCM (20 mL, anhydrous) under N_2 (Schlenk technique). Then, TBSCl (2.261 g, 15.001 mmol) was slowly added as a solution in DCM (5 mL, anhydrous). The reaction was stirred for 3 hr, then diluted with DCM and quenched with H_2O. The layers were separated, and the aqueous layer was washed with DCM (3 x 20 mL). The combined organic layers were dried over Na_2SO_4, filtered, and concentrated. Purification by silica gel column chromatography (8% ethyl acetate in hexane) afforded **S1** (2.523 g, 91%) as a colorless oil. ^{1}H NMR (600 MHz, $CDCl_3$): δ 6.77 (d, *J* = 8.0 Hz, 1H), 6.67 (d, *J* = 2.0 Hz, 1H), 6.63 (dd, *J* = 8.0, 2.0 Hz, 1H), 5.96 (ddt, *J* = 16.8, 10.1, 6.6 Hz, 1H), 5.09–5.03 (m, 2H), 3.79 (s, 3H), 3.32 (d, *J* = 6.7 Hz, 2H), 0.99 (s, 9H), 0.14 (s, 6H); ^{13}C NMR (151 MHz, $CDCl_3$): δ 150.8, 143.3, 137.8, 133.5, 120.7, 120.7, 115.5, 112.6, 55.5, 39.9, 25.8, 18.5, -4.6. The spectra are consistent with those found in literature.[S6]

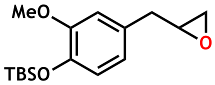

1M

S1 (2.508 g, 9.005 mmol) was reacted for 16 hr according to **General Procedure 2**. Purification by silica gel column chromatography (5-10% ethyl acetate in hexane) afforded **1M** (1.304 g, 49%) as a yellow oil. ^{1}H NMR (600 MHz, $CDCl_3$): δ 6.78 (d, *J* = 8.0 Hz, 1H), 6.74 (d, *J* = 1.9 Hz, 1H), 6.69 (dd, *J* = 8.0, 2.0 Hz, 1H), 3.80 (s, 3H), 3.16-3.12 (m, 1H), 2.85-2.75 (m, 3H), 2.54 (dd, *J* = 5.0, 2.7 Hz, 1H), 0.99 (s, 9H), 0.15 (s, 6H); ^{13}C NMR (151 MHz, $CDCl_3$): δ 150.9, 143.8, 130.6, 121.1, 120.8, 113.0, 55.5, 52.7, 46.9, 38.4, 25.8, 18.5, -4.6; MS (ESI) calculated for $C_{16}H_{27}O_3Si$ $[M+H]^+$: 295.1729, observed 295.1725.

Synthesis of 2-methyl-3-(phenoxymethyl)oxirane (1R)

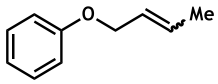

S2

In a 100-mL round-bottom flask equipped with magnetic stir bar, phenol (0.995 g, 10.568 mmol, 1.0 equiv) was dissolved in acetone (12 mL). To this mixture, K_2CO_3 (2.937 g, 21.252 mmol, 2.0 equiv) and crotyl bromide (2.0 mL, 16.522 mmol, 1.6 equiv) were added sequentially. The mixture was stirred at 56 °C (w/ reflux condenser) for 20 hr. Upon completion, the reaction was cooled to room temperature and concentrated. The resulting residue was re-suspended in Et_2O (30 mL) and washed with 1M NaOH and H_2O. The organic layer was then dried over Na_2SO_4, filtered, and concentrated. Purification by silica gel column chromatography (10% ethyl ether in hexane) afforded **S2** (1.263 g, 81%, 4:1 *E*:*Z* isomers) as a colorless oil.

Spectroscopic data for **S2, *E-isomer***: ^{1}H NMR (600 MHz, $CDCl_3$): δ 7.31-7.26 (m, 2H), 6.97-6.90 (m, 3H), 5.90-5.83 (m, 1H), 5.79-5.69 (m, 1H), 4.46 (d, *J* = 6.1 Hz, 2H),1.77 (d, *J* = 6.4 Hz, 3H); ^{13}C NMR (151 MHz, $CDCl_3$): δ 158.8, 130.7, 129.6, 126.3, 120.8, 114.8, 68.7, 18.0. The spectra are consistent with those found in literature.[S7]

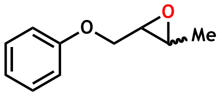

1R

S2 (1.003 g, 6.767 mmol) was reacted for 48 hr according to **General Procedure 2**. Purification by silica gel column chromatography (20% ethyl acetate in hexane) afforded **1R** (0.764 g, 69%, 4:1 *dr*) as colorless oil. Spectroscopic data for the ***major diastereomer***: ^{1}H NMR (600 MHz, $CDCl_3$): δ 7.32-7.27 (m, 2H), 7.00-6.90 (m, 3H), 4.15 (dd, *J* = 10.9, 3.6 Hz, 1H), 4.00 (dd, *J* = 11.0, 5.3 Hz, 1H), 3.09-3.07 (m, 1H), 3.07-3.03 (m, 1H), 1.39 (d, *J* = 5.2 Hz, 3H); ^{13}C NMR (151 MHz, $CDCl_3$): δ 158.6, 129.6, 121.3, 114.7, 68.3, 57.2, 52.8, 17.5; MS (ESI) calculated for $C_{10}H_{12}O_2Na$ $[M+Na]^+$: 187.0735, observed 187.0723.

Cobaloxime-photocatalyzed Epoxide Isomerization

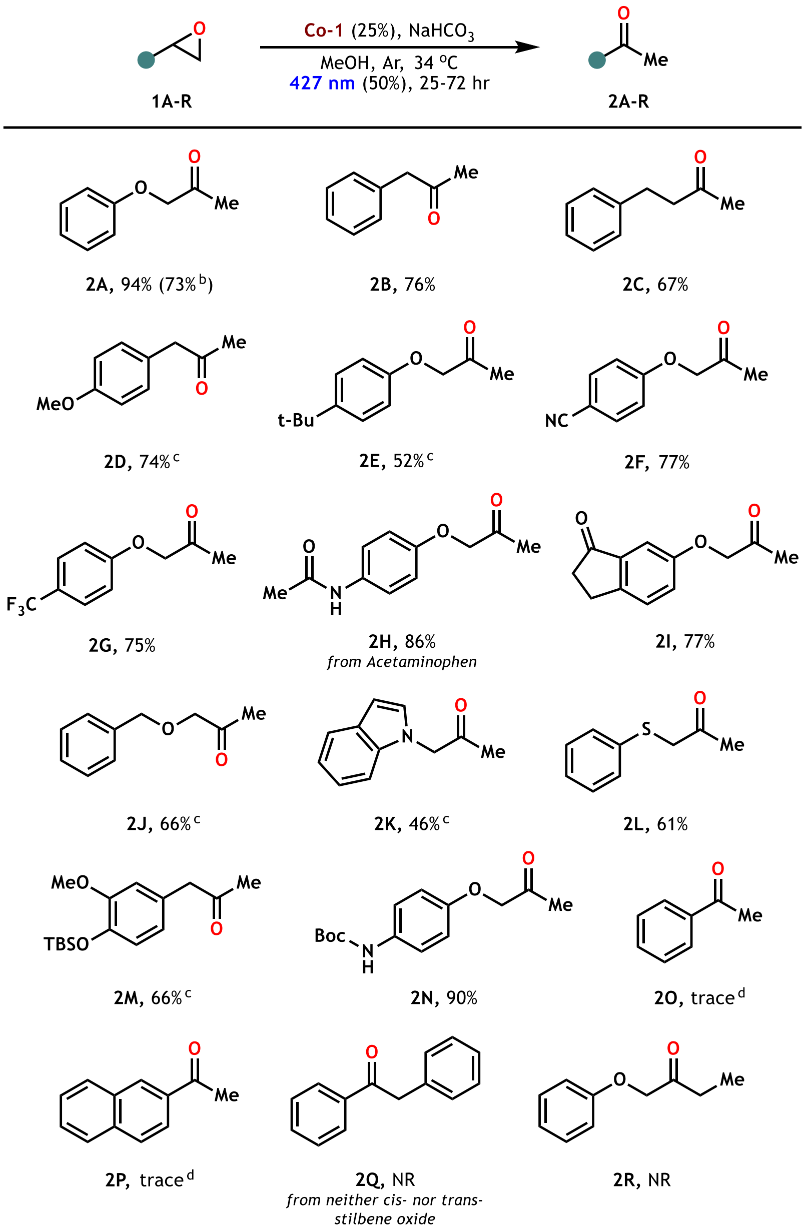

Figure S2. Cobaloxime-photocatalyzed epoxide isomerization

[a] *Reaction conditions:* epoxide (**1A-R**, 1.0 equiv), **Co-1** (25 mol %), $NaHCO_3$ (1.0 equiv), MeOH (degassed with Argon, 0.2 M), 427 nm irradiation (Kessil®, 50% intensity). Isolated yield.

[b] 1-mmol scale.

[c] Low conversion under standard conditions. Increased loading of **Co-1** (35 mol %).

[d] Less than 5%. Yield determined by ^{1}H NMR using 3,4,5-trichloropyridine as an internal standard.

General Procedure 4

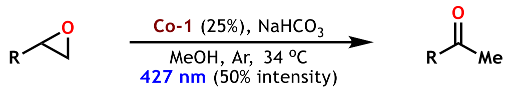

1 2

To an 8-mL septa-capped, borosilicate glass vial equipped with magnetic stir bar was added **Co-1** (20.6 mg, 0.05 mmol, 25 mol %) and $NaHCO_3$ (16.8 mg, 0.2 mmol, 1.0 equiv). The vial was then purged with Argon (balloon) for 3 min. Epoxide (**1**, 0.2 mmol, 1.0 equiv) and MeOH (degassed with Argon, 1 mL) were also added. Finally, the septum was sealed with grease and covered with tape. The reaction was stirred and irradiated with 427-nm LED (set to 50% intensity) for 25-72 hours. The light source (Kessil® PR160L-427 nm) was positioned approximately 2 cm from the vial (see **Figure S1**), unfiltered, and the apparatus was enclosed in an aluminum foil "igloo" during irradiation. Upon completion, the reaction mixture was purified by Preparative TLC to afford the ketone products (**2**) shown below.

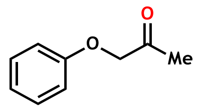

2A

Glycidyl phenyl ether (**1A,** 27 μL, 0.200 mmol) was reacted for 25 hr according to **General Procedure 4**. Purification by Preparative TLC (20% ethyl ether in hexane) afforded **2A** (28.3 mg, 94%) as a colorless oil. ^{1}H NMR (600 MHz, $CDCl_3$): δ 7.33-7.28 (m, 2H), 7.02-6.98 (m, 1H), 6.89 (d, J = 8.8 Hz, 2H), 4.54 (s, 2H), 2.28 (s, 3H); ^{13}C NMR (151 MHz, $CDCl_3$): δ 206.1, 157.8, 129.8, 121.9, 114.6, 73.1, 26.8. The spectra are consistent with those found in literature.[S8]

At larger scale (1-mmol), glycidyl phenyl ether (**1A,** 136 μL, 1.005 mmol) was reacted for 40 hr according to **General Procedure 4**. Upon completion, the reaction mixture was concentrated to generate the crude product. Purification by silica gel column chromatography (30% ethyl acetate in hexane) afforded **2A** (110.0 mg, 73%) as a colorless oil. The resulting spectra matched those of our 0.2-mmol reaction above as well as those found in literature.[S8]

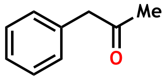

2B

(2,3-epoxypropyl)benzene (**1B,** 26 μL, 0.198 mmol) was reacted for 25 hr according to **General Procedure 4**. Purification by Preparative TLC (20% ethyl ether in hexane) afforded **2B** (20.2 mg, 76%) as a colorless oil. ^{1}H NMR (600 MHz, $CDCl_3$): δ 7.36-7.32 (m, 2H), 7.29-7.26 (m, 1H), 7.22-7.19 (m, 2H), 3.70 (s, 2H), 2.16 (s, 3H); ^{13}C NMR (151 MHz, $CDCl_3$): δ 206.6, 134.4, 129.5, 128.9, 127.2, 51.2, 29.4. The spectra are consistent with those found in literature.[S8]

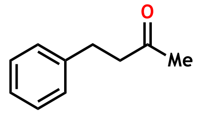

2C

Epoxide **1C** (29 μL, 0.201 mmol) was reacted for 25 hr according to **General Procedure 4**. Purification by Preparative TLC (20% ethyl ether in hexane) afforded **2C** (19.8 mg, 67%) as a colorless oil. ^{1}H NMR (600 MHz, $CDCl_3$): δ 7.28 (t, J = 7.6 Hz, 2H), 7.22-7.17 (m, 3H), 2.90 (t, J = 7.5 Hz, 2H), 2.77 (t, J = 7.7 Hz, 2H), 2.15 (s, 3H); ^{13}C NMR (151 MHz, $CDCl_3$): δ 208.1, 141.1, 128.6, 128.4, 126.2, 45.3, 30.2, 29.9. The spectra are consistent with those found in literature.[S8]

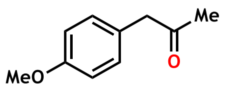

2D

Epoxide **1D** (32.6 mg, 0.199 mmol) was reacted for 61 hr according to **General Procedure 4,** with increased loading of **Co-1** (35%). Purification by Preparative TLC (30% ethyl acetate in hexane) afforded **2D** (24.1 mg, 74%) as a pale yellow oil. ^{1}H NMR (600 MHz, $CDCl_3$): δ 7.12 (d, *J* = 8.6 Hz, 2H), 6.87 (d, *J* = 8.7 Hz, 2H), 3.80 (s, 3H), 3.63 (s, 2H), 2.14 (s, 3H); ^{13}C NMR (151 MHz, $CDCl_3$): δ 206.9, 158.7, 130.4, 126.3, 114.2, 55.3, 50.2, 29.2. The spectra are consistent with those found in literature.[S9]

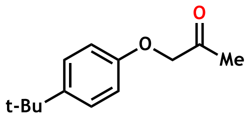

2E

Epoxide **1E** (41.0 mg, 0.199 mmol) was reacted for 52 hr according to **General Procedure 4**, with increased loading of **Co-1** (35%). Purification by Preparative TLC (10% acetic acid, 20% ethyl ether in hexane) afforded **2E** (21.3 mg, 52%) as a yellow oil. ^{1}H NMR (600 MHz, $CDCl_3$): δ 7.32 (d, *J* = 8.8 Hz, 2H), 6.82 (d, *J* = 8.8 Hz, 2H), 4.52 (s, 2H), 2.28 (s, 3H), 1.30 (s, 9H); ^{13}C NMR (151 MHz, $CDCl_3$): δ 206.4, 155.6, 144.6, 126.6, 114.1, 73.4, 34.3, 31.6, 26.8; The spectra are consistent with those found in literature.[S10]

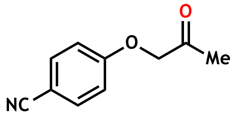

2F

Epoxide **1F** (35.1 mg, 0.200 mmol) was reacted for 25 hr according to **General Procedure 4**. Purification by Preparative TLC (50% ethyl acetate in hexane) afforded **2F** (27.0 mg, 77%) as a pale yellow oil. ^{1}H NMR (600 MHz, $CDCl_3$): δ 7.60 (d, *J* = 9.0 Hz, 2H), 6.93 (d, *J* = 8.9 Hz, 2H), 4.62 (s, 2H), 2.28 (s, 3H); ^{13}C NMR (151 MHz, $CDCl_3$): δ 203.6, 160.9, 134.3, 118.9, 115.4, 105.3, 72.8, 26.7; MS (ESI) calculated for $C_{10}H_{10}NO_2$ $[M+H]^+$: 176.0712, observed 176.0702.

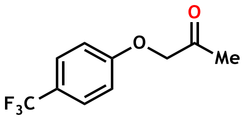

2G

Epoxide **1G** (45.5 mg, 0.209 mmol) was reacted for 28 hr according to **General Procedure 4**. Purification by Preparative TLC (30% ethyl acetate in hexane) afforded **2G** (34.2 mg, 75%) as a pale yellow oil. ^{1}H NMR (600 MHz, $CDCl_3$): δ 7.56 (d, *J* = 9.1 Hz, 2H), 6.95 (d, *J* = 8.3 Hz, 2H), 4.60 (s, 2H), 2.29 (s, 3H); ^{13}C NMR (151 MHz, $CDCl_3$): δ 204.5, 160.2, 127.3 (q_{C-F}, *J* = 3.9 Hz), 124.3 (q_{C-F}, *J* = 270.8 Hz), 124.1 (q_{C-F}, *J* = 33.0 Hz), 114.7, 73.0, 26.7; ^{19}F NMR (594 MHz, $CDCl_3$): δ -61.7; MS (ESI) calculated for $C_{10}H_{19}F_3O_2Na[M+Na]^+$: 241.0452, observed 241.0455.

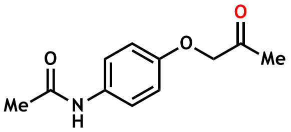

2H

from Acetaminophen

Epoxide **1H** (41.4 mg, 0.199 mmol) was reacted for 26 hr according to **General Procedure 4**. Purification by Preparative TLC (90% ethyl acetate in hexane) afforded **2H** (35.5 mg, 86%) as an off-white solid. ^{1}H NMR (600 MHz, $CDCl_3$): δ 7.41 (d, *J* = 8.8 Hz, 2H), 7.15 (bs, 1H), 6.84 (d, *J* = 8.9 Hz, 2H), 4.52 (s, 2H), 2.27 (s, 3H), 2.16 (s, 3H); ^{13}C NMR (151 MHz, $CDCl_3$): δ 205.9, 168.3, 154.6, 132.1, 122.0, 115.0, 73.5, 26.8, 24.6; MS (ESI) calculated for $C_{11}H_{14}NO_3$ $[M+H]^+$: 208.0974, observed 208.0970.

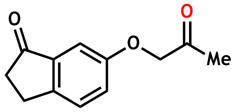

2I

Epoxide **1I** (40.9 mg, 0.200 mmol) was reacted for 25 hr according to **General Procedure 4**. Purification by Preparative TLC (70% ethyl acetate in hexane) afforded **2I** (31.5 mg, 77%) as a pale yellow oil. ^{1}H NMR (600 MHz, $CDCl_3$): δ 7.41 (d, *J* = 8.3 Hz, 1H), 7.26 (dd, *J* = 8.6, 2.8 Hz, 1H), 7.09 (d, *J* = 2.5 Hz, 1H), 4.62 (s, 2H), 3.10-3.06 (m, 2H), 2.74-2.70 (m, 2H), 2.28 (s, 3H); ^{13}C NMR (151 MHz, $CDCl_3$): δ 206.8, 204.3, 157.5, 149.0, 138.4, 127.9, 124.3, 105.9, 73.1, 37.1, 26.7, 25.3; MS (ESI) calculated for $C_{12}H_{13}O_3$ $[M+H]^+$: 205.0865, observed 205.0856.

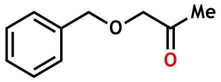

2J

Benzyl glycidyl ether (**1J,** 30.5 μL, 0.200 mmol) was reacted for 72 hr according to **General Procedure 4,** with increased loading of **Co-1** (35%). Purification by Preparative TLC (25% ethyl ether in hexane) afforded **2J** (21.7 mg, 66%) as a colorless oil. ^{1}H NMR (600 MHz, $CDCl_3$): δ 7.39-7.29 (m, 5H), 4.60 (s, 2H), 4.06 (s, 2H), 2.17 (s, 3H); ^{13}C NMR (151 MHz, $CDCl_3$): δ 206.8, 137.3, 128.7, 128.2, 128.0, 75.4, 73.4, 26.6. The spectra are consistent with those found in literature.[S10]

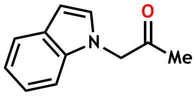

2K

Epoxide **1K** (34.9 mg, 0.201 mmol) was reacted for 59 hr according to **General Procedure 4,** with increased loading of **Co-1** (35%). Purification by Preparative TLC (40% ethyl acetate in hexane) afforded an inseparable mixture of **2K** (16.1 mg, 46%) with **1K** (0.8 mg, 2%) as a yellow oil. ^{1}H NMR (600 MHz, $CDCl_3$): δ 7.67 (d, *J* = 7.9 Hz, 1H), 7.25-7.23 (m, 1H), 7.19-7.14 (m, 2H), 7.07 (d, *J* = 3.2 Hz, 1H), 6.61 (dd, *J* = 3.1, 0.6 Hz, 1H), 4.81 (s, 2H), 2.02 (s, 3H); ^{13}C NMR (151 MHz, $CDCl_3$): δ 204.5, 136.4, 128.2, 122.4, 121.3, 120.1, 108.8, 102.9, 56.1, 26.7. The spectra are consistent with those found in literature.[S11]

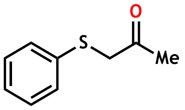

2L

Epoxide **1L** (33.3 mg, 0.200 mmol) was reacted for 40 hr according to **General Procedure 4**. Purification by Preparative TLC (12% ethyl acetate in hexane) afforded **2L** (20.3 mg, 61%) as a pale-yellow liquid. ^{1}H NMR (600 MHz, $CDCl_3$) δ 7.36-7.32 (m, 2H), 7.29 (t, *J* = 7.7 Hz, 2H), 7.24-7.20 (m, 1H), 3.67 (s, 2H), 2.28 (s, 3H); ^{13}C NMR (151 MHz, $CDCl_3$) δ 203.6, 134.7, 129.5, 129.2, 126.9, 44.7, 28.0. The spectra are consistent with those reported in literature.[S12]

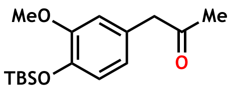

2M

Epoxide **1M** (58.7 mg, 0.199 mmol) was reacted for 42 hr according to **General Procedure 4**, with increased loading of **Co-1** (35%). Purification by Preparative TLC (15% ethyl acetate in hexane) afforded **2M** (39.0 mg, 66%) as a yellowish oil. ^{1}H NMR (600 MHz, $CDCl_3$) δ 6.80 (d, *J* = 7.8 Hz, 1H), 6.69–6.64 (m, 2H), 3.79 (s, 3H), 3.60 (s, 2H), 2.12 (s, 3H), 0.99 (s, 9H), 0.15 (s, 6H); ^{13}C NMR (151 MHz, $CDCl_3$) δ 207.1, 151.1, 144.2, 127.7, 121.8, 121.1, 113.1, 55.5, 50.9, 29.0, 25.7, 18.5, -4.6; MS (ESI) calculated for $C_{16}H_{27}O_3SiNa$ $[M+H]^+$: 317.1549, observed 317.1545.

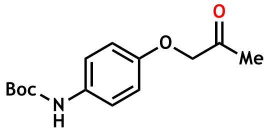

2N

Epoxide **1N** (53.0 mg, 0.200 mmol) was reacted for 40 hr according to **General Procedure 4**. Purification by Preparative TLC (30% ethyl acetate in hexane) afforded **2M** (47.6 mg, 90%) as a white powder. ^{1}H NMR (600 MHz, $CDCl_3$) δ 7.28 (d, *J* = 7.9 Hz, 2H), 6.84-6.80 (m, 2H), 6.38 (s, 1H), 4.50 (s, 2H), 2.26 (s, 3H), 1.50 (s, 9H); ^{13}C NMR (151 MHz, $CDCl_3$) δ 206.0, 153.7, 153.0, 132.5, 120.5, 115.0, 80.4, 73.5, 28.4, 26.6; MS (ESI) calculated for $C_{14}H_{19}NO_4Na$ $[M+Na]^+$: 288.1212, observed 288.1208.

Mechanistic Investigations

TEMPO Radical Trap Experiment

Table S2. Effect of TEMPO radical trap on cobaloxime-photocatalyzed epoxide isomerization

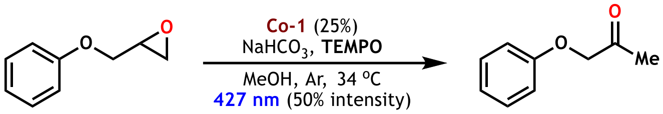

1A **2A**

Entry	TEMPO (equiv)	Conversion (1A, %)	Yield (2A, %)
1	none	96	88
2	1.1	18	none

Reaction conditions: **Co-1** (0.04 mmol, 25 mol %) and $NaHCO_3$ (0.2 mmol, 1.0 equiv) were added to an 8-mL septa-capped vial equipped with a magnetic stir bar. The vial was sparged with Argon for 5 minutes before **1A** (0.2 mmol, 1.0 equiv) was added and the mixture was dissolved in Argon-degassed MeOH (0.2 M). The septum was sealed with grease and covered with tape, and the reaction was stirred and irradiated with 427-nm LED (Kessil®, 50% intensity) for 22 h. Yields determined by ^1H NMR using 1,3,5-trimethoxybenzene as an internal standard.

Radical Clock Experiment

Table S3. Cobaloxime-photocatalyzed isomerization of radical clock substrate **1S**

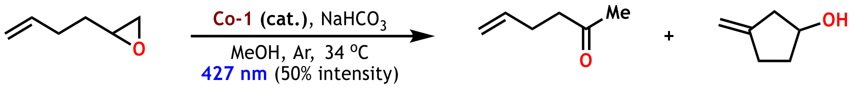

1S **2S** **4S**

Entry	TEMPO (mol %)	Conversion (1S, %)	Yield (2S, %)	Yield (4S, %)
1	25	64	23	5
2	35	66	26	12

Reaction conditions: **Co-1** (cat.) and $NaHCO_3$ (0.2 mmol, 1.0 equiv) were added to an 8-mL septa-capped vial equipped with a magnetic stir bar. The vial was sparged with Argon for 5 minutes before **1S** (0.2 mmol, 1.0 equiv) was added and the mixture was dissolved in Argon-degassed MeOH (0.2 M). The septum was sealed with grease and covered with tape, and the reaction was stirred and irradiated with 427-nm LED (Kessil®, 50% intensity) for 22 h. Yields determined by ^1H NMR using 3,4,5-trichloropyridine as an internal standard.

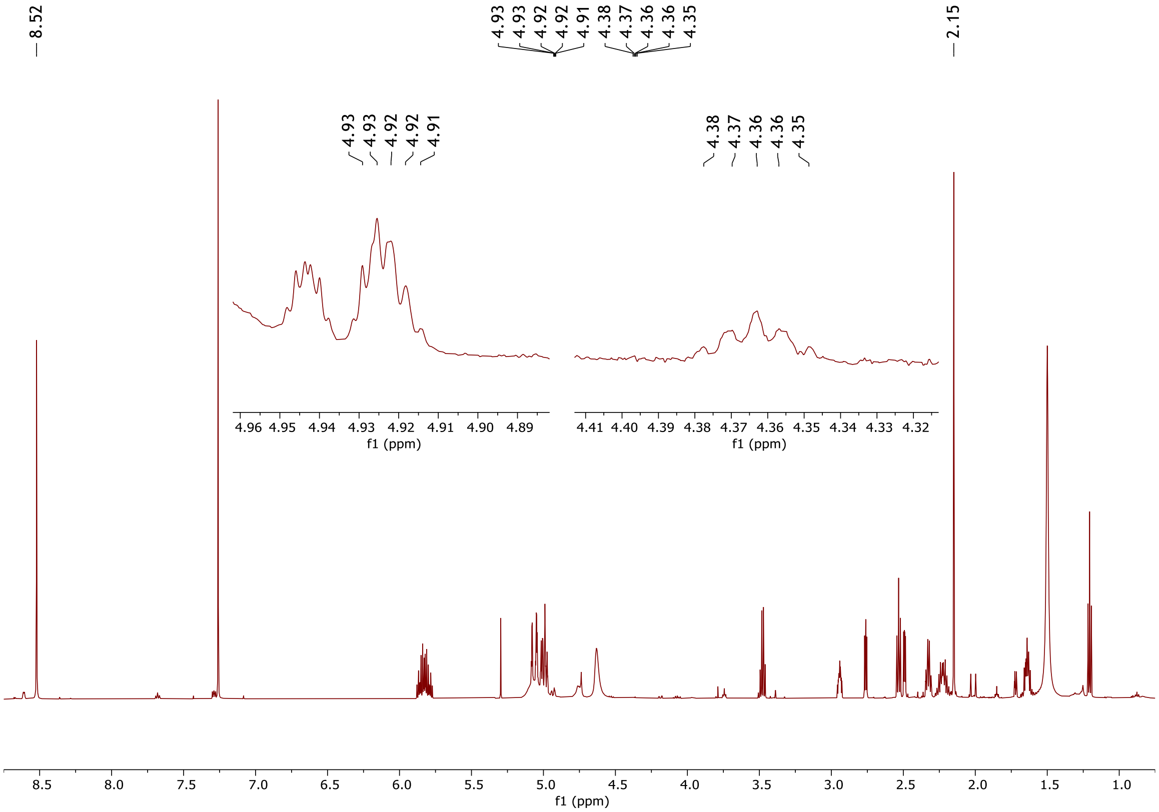

Figure S3. Crude ^{1}H NMR (600 MHz, $CDCl_3$) of **Table S3,** entry 2 reaction products, with **4S** characteristic peaks emphasized.

Synthesis of Radical Clock Dopant (4S, 3-methylenecyclopentan-1-ol)

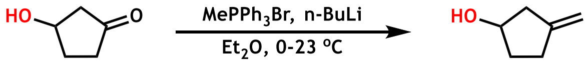

S3 4S

In a 100-mL two-necked round bottom flask equipped with metal stir bar, methyltriphenylphosphonium bromide (1.965 g, 5.501 mmol) was dissolved in ethyl ether (20 mL, anhydrous) and brought to 0 °C (ice bath). While on ice, n-BuLi (2.5 M in hexane, 2.2 mL, 5.5 mmol) was slowly added via syringe and the resulting reddish solution was allowed to stir for 30 min. Then, still at 0 °C, **S3** was added to the mixture as a solution in ethyl ether (3 mL, anhydrous). Upon addition, the reaction was allowed to warm to room temperature and stirred overnight. Upon completion (monitored by TLC), the reaction was quenched with slow addition of H_2O (20 mL). The organic layer was separated and washed with H_2O (2x) and brine, then dried over Na_2SO_4, filtered, and concentrated. **4S** was isolated by silica gel column chromatography (40-60% ethyl ether in pentane). Due to its high volatility, complete purification of **4S** was very challenging. Consequently, a solution of **4S** in $CDCl_3$ was produced (*also containing ethyl ether, acetone, and pentane as impurities*) for use as an NMR dopant to better display formation of **4S** in the reaction of radical clock substrate **1S.**

^{1}H NMR (600 MHz, $CDCl_3$): δ 4.92 (p, J = 2.1 Hz, 2H), 4.37 (p, J = 3.9 Hz, 1H), 2.58-2.47 (m, 2H), 2.35-2.27 (m, 2H), 1.94-1.86 (m, 1H), 1.77-1.72 (m, 1H). The spectrum is consistent with that found in literature.[S13]

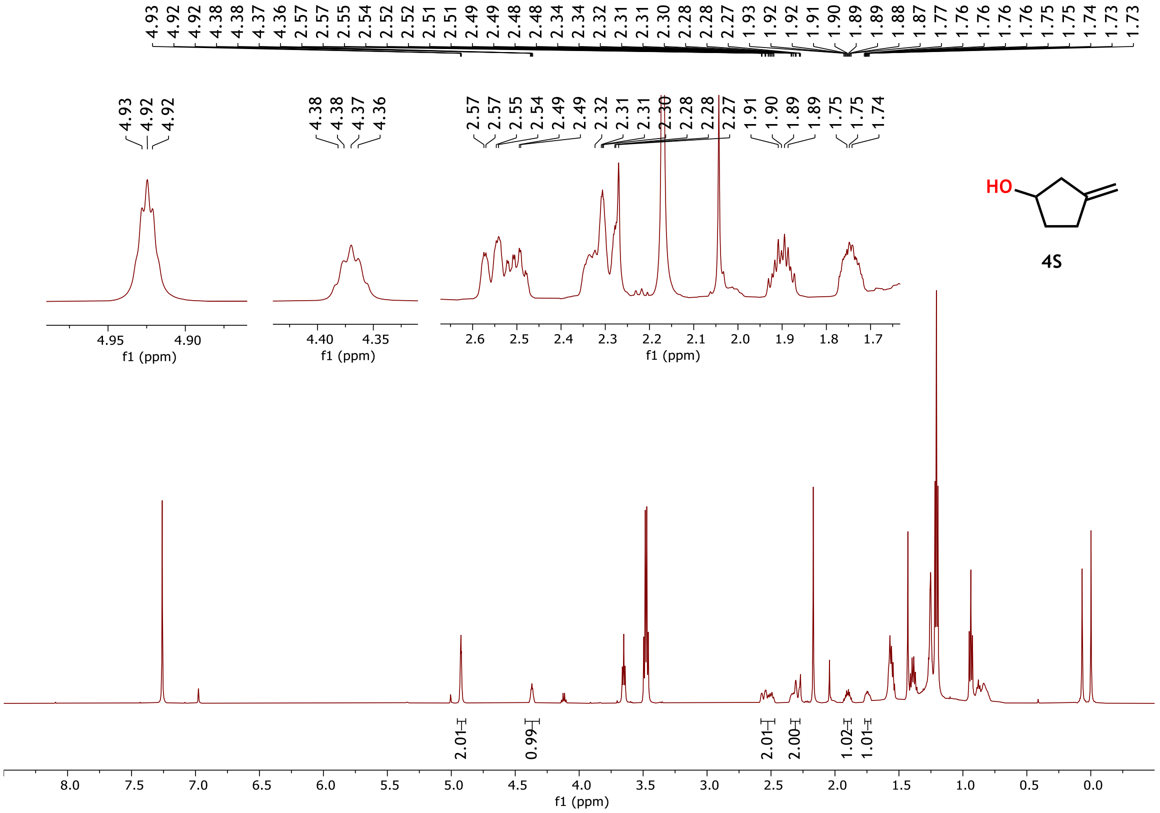

Figure S4. ^{1}H NMR (600 MHz, $CDCl_3$) of Radical Clock Dopant (containing **4S**, Et_2O, pentane, acetone).

This solution of **4S** was used to sequentially dope the crude radical clock reaction sample (see **Figure S3**). As shown below, the peaks we suspected belonged to **4S** (pentet at 4.92 ppm, pentet at 4.36 ppm) indeed grow in intensity with each addition of dopant solution (see **Figure S5**).

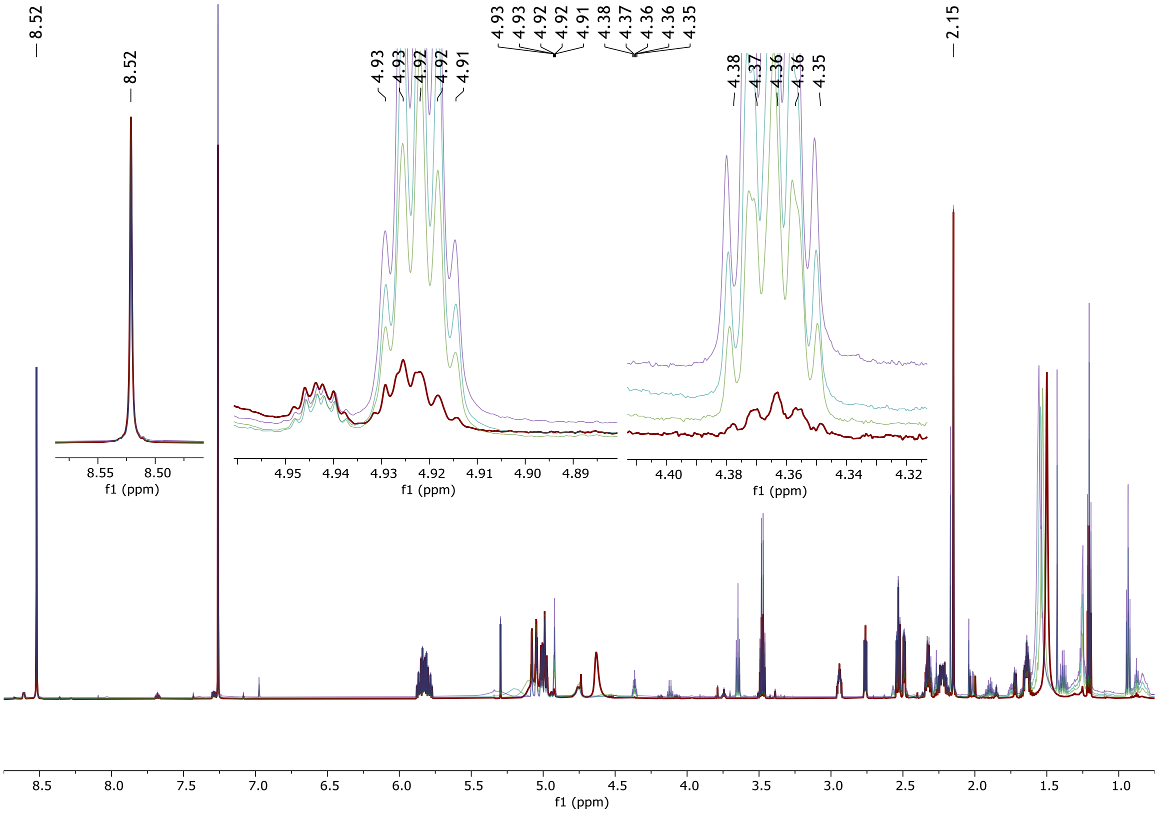

Figure S5. Superimposed crude ^1H NMR (600 MHz, $CDCl_3$) spectra of epoxide **1S** reaction products, before and after sequential doping with **4S**.

Note: Superimposed spectra in **Figure S5** are normalized to the intensity of 3,4,5-trichloropyridine internal standard peak at 8.52 ppm.

Proposed Mechanism

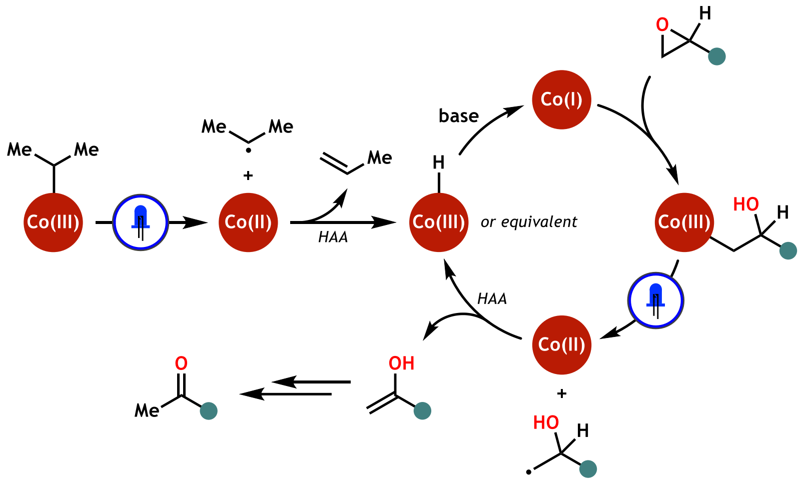

Figure S8. Mechanistic proposal for cobaloxime-photocatalyzed epoxide isomerization

References

S1. Weiss, M. E.; Kreis, L. M.; Lauber, A.; Carreira, E. M. Cobalt-Catalyzed Coupling of Alkyl Iodides with Alkenes: Deprotonation of Hydridocobalt Enables Turnover. *Angew. Chem. Int. Ed.* **2011**, *50* (47), 11125–11128. https://doi.org/10.1002/anie.201105235.

S2. Gupta, B. D.; Qanungo, K. A Study of Cis Influence in Alkyl Cobaloximes. *J. Organomet. Chem.* **1997**, *543* (1–2), 125–134. https://doi.org/10.1016/S0022-328X(97)00210-6.

S3. Funk, B. E.; Pauze, M.; Lu, Y.-C.; Moser, A. J.; Wolf, G.; West, J. G. Vitamin B12 and Hydrogen Atom Transfer Cooperative Catalysis as a Hydride Nucleophile Mimic in Epoxide Ring Opening. *Cell Rep. Phys. Sci.* **2023**, *4* (4), 101372. https://doi.org/10.1016/j.xcrp.2023.101372.

S4. Genç, S.; Gülcemal, S.; Günnaz, S.; Çetinkaya, B.; Xiao, J.; Gülcemal, D. Regioselective Synthesis of Alcohols by Catalytic Transfer Hydrogenation of Epoxides. *J. Org. Chem.* **2025**, *90* (38), 13508–13519. https://doi.org/10.1021/acs.joc.5c01342.

S5. Cherfaoui, B.; Guo, T.; Sun, H.-P.; Cheng, W.-L.; Liu, F.; Jiang, F.; Xu, X.-L.; You, Q.-D. Synthesis and Evaluation of 4-(2-Hydroxypropyl)Piperazin-1-Yl) Derivatives as Hsp90 Inhibitors. *Bioorg. Med. Chem.* **2016**, *24* (11), 2423–2432. https://doi.org/10.1016/j.bmc.2016.03.049.

S6. Schultz, D. C.; Rathnayake, U.; Duncan, R. A.; Richardson, A. E.; Bender, A. M. Synthesis of the Dimeric Diarylheptanoids Alpinidinoid C and Officinine B Enabled by Blue-Light-Mediated Triple-Minisci-Type Alkylation. *Org. Lett.* **2024**, *26* (42), 9028–9033. https://doi.org/10.1021/acs.orglett.4c03227.

S7. Ning, X.-S.; Wang, M.-M.; Yao, C.-Z.; Chen, X.-M.; Kang, Y.-B. *Tert* -Butyl Nitrite: Organic Redox Cocatalyst for Aerobic Aldehyde-Selective Wacker–Tsuji Oxidation. *Org. Lett.* **2016**, *18* (11), 2700–2703. https://doi.org/10.1021/acs.orglett.6b01165.

S8. Zhang, S.; Zhang, J.; Zou, H. Pd-Catalyzed TBHP-Mediated Selective Wacker-Type Oxidation and Oxo-Acyloxylation of Olefins Using a 2-(1 *H* -Indazol-1-Yl)Quinoline Ligand. *Org. Lett.* **2023**, *25* (11), 1850–1855. https://doi.org/10.1021/acs.orglett.3c00326.

S9. Purohit, V. C.; Allwein, S. P.; Bakale, R. P. Catalytic Oxidative 1,2-Shift in 1,1′-Disubstituted Olefins Using Arene(Iodo)Sulfonic Acid as the Precatalyst and Oxone as the Oxidant. *Org. Lett.* **2013**, *15* (7), 1650–1653. https://doi.org/10.1021/ol400432x.

S10. Chaudhari, D. A.; Fernandes, R. A. Hypervalent Iodine as a Terminal Oxidant in Wacker-Type Oxidation of Terminal Olefins to Methyl Ketones. *J. Org. Chem.* **2016**, *81* (5), 2113–2121. https://doi.org/10.1021/acs.joc.6b00137.

S11. Zhao, L.; Yan, Z.-H.; Tang, S.; Wei, Z.-L.; Liao, W.-W. Brønsted Acid-Promoted Cyclodimerization of Indolyl Ketones: Construction of Indole Fused-Oxabicyclo[3.3.1]Nonane and -Cyclooctatetraene Ring Systems. *Org. Lett.* **2021**, *23* (1), 166–171. https://doi.org/10.1021/acs.orglett.0c03895.

S12. de Souza, A. A. N.; Bartolomeu, A. de A.; Brocksom, T. J.; Noël, T.; de Oliveira, K. T. Direct Synthesis of α-Sulfenylated Ketones under Electrochemical Conditions. *J. Org. Chem.* **2022**, *87* (9), 5856–5865. https://doi.org/10.1021/acs.joc.2c00147.

S13. Prina Cerai, G.; Morandi, B. Atom-Economical Cobalt-Catalysed Regioselective Coupling of Epoxides and Aziridines with Alkenes. *Chem. Comm.* **2016**, *52* (63), 9769–9772. https://doi.org/10.1039/C6CC04410G.

^{1}H. ^{13}C, and ^{19}F NMR Spectra

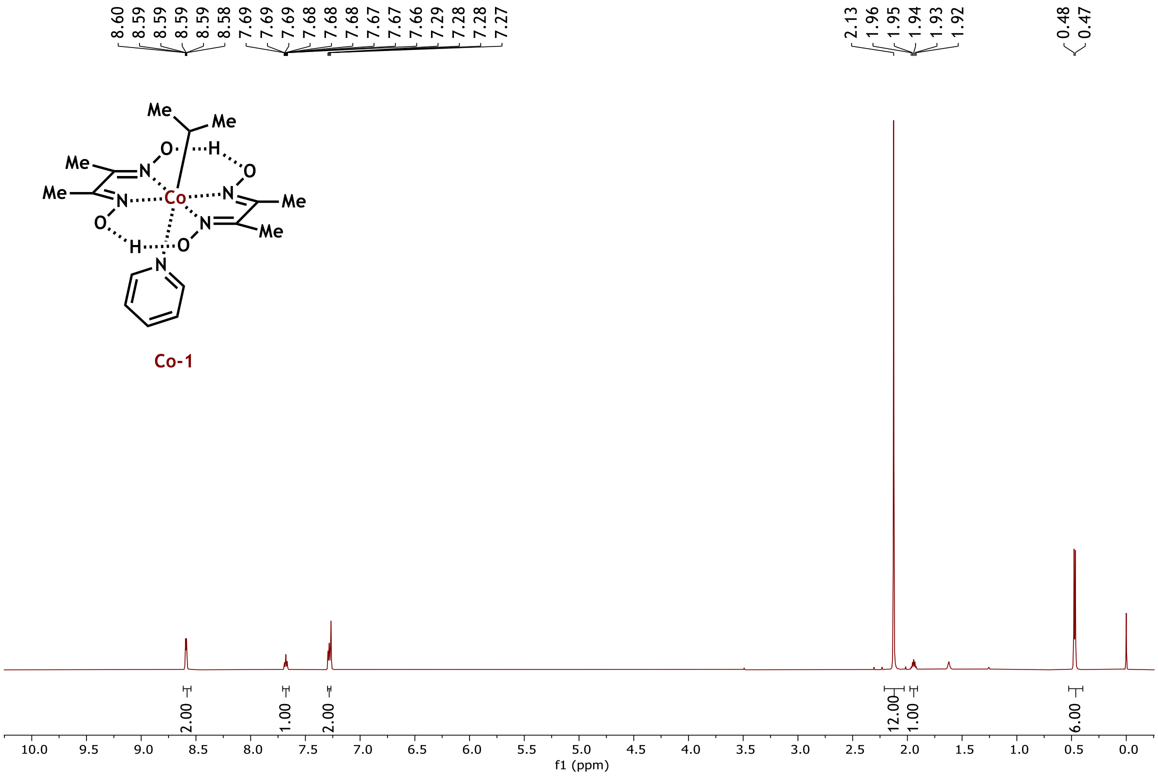

Figure S9. ^{1}H NMR (600 MHz, $CDCl_3$) of [Co(dmgH)$_2$(pyr)*i*-Pr] (**Co-1**)

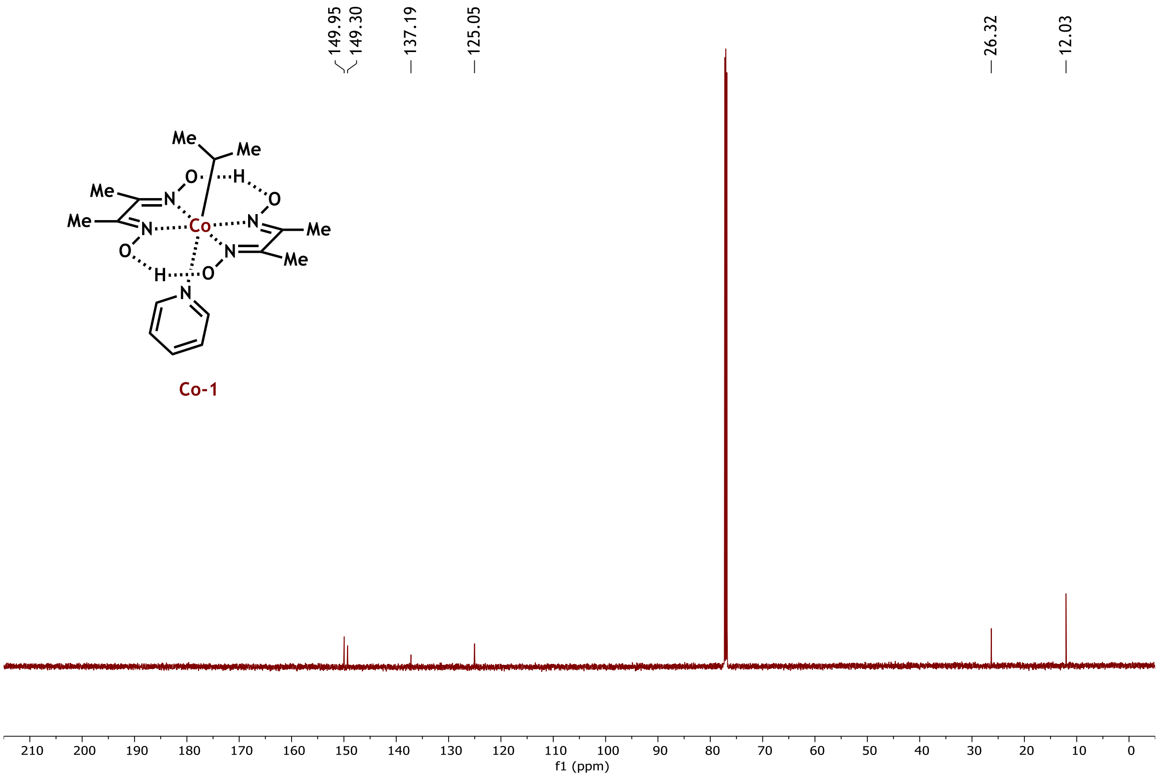

Figure S10. ^{13}C NMR (151 MHz, $CDCl_3$) of [Co(dmgH)$_2$(pyr)*i*-Pr] (**Co-1**)

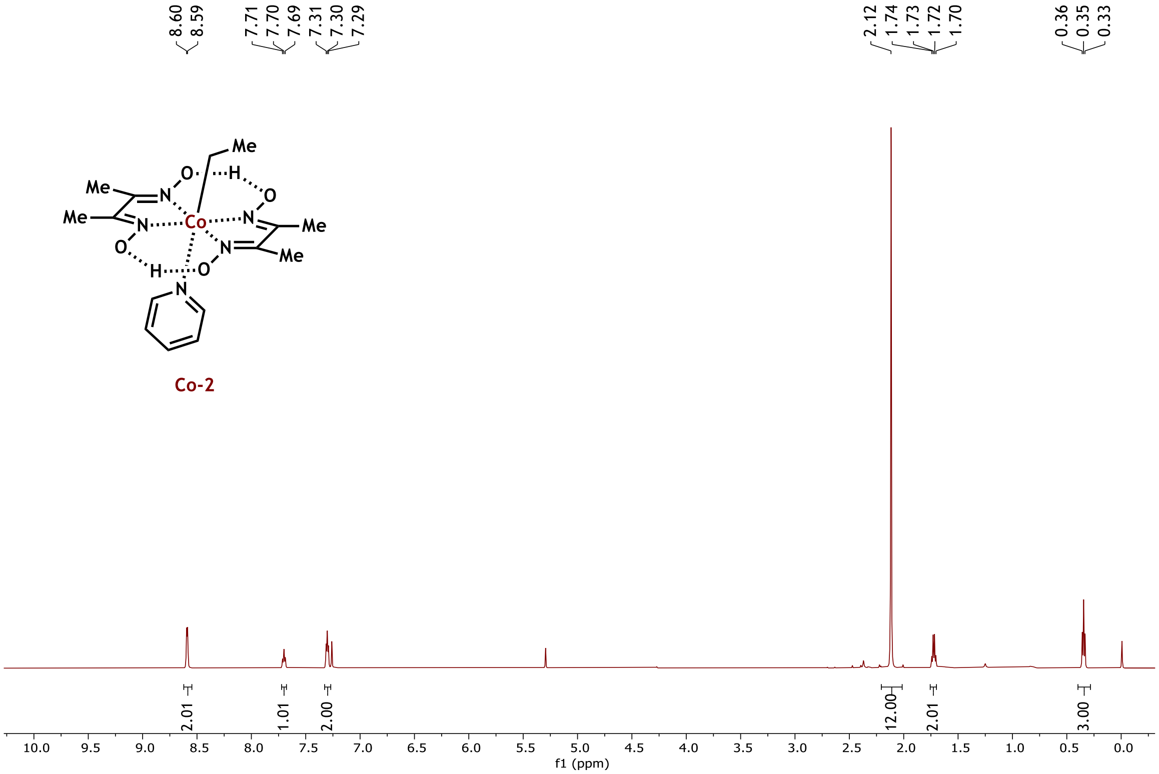

Figure S11. ^{1}H NMR (600 MHz, $CDCl_3$) of [Co(dmgH)$_2$(pyr)Et] (**Co-2**)

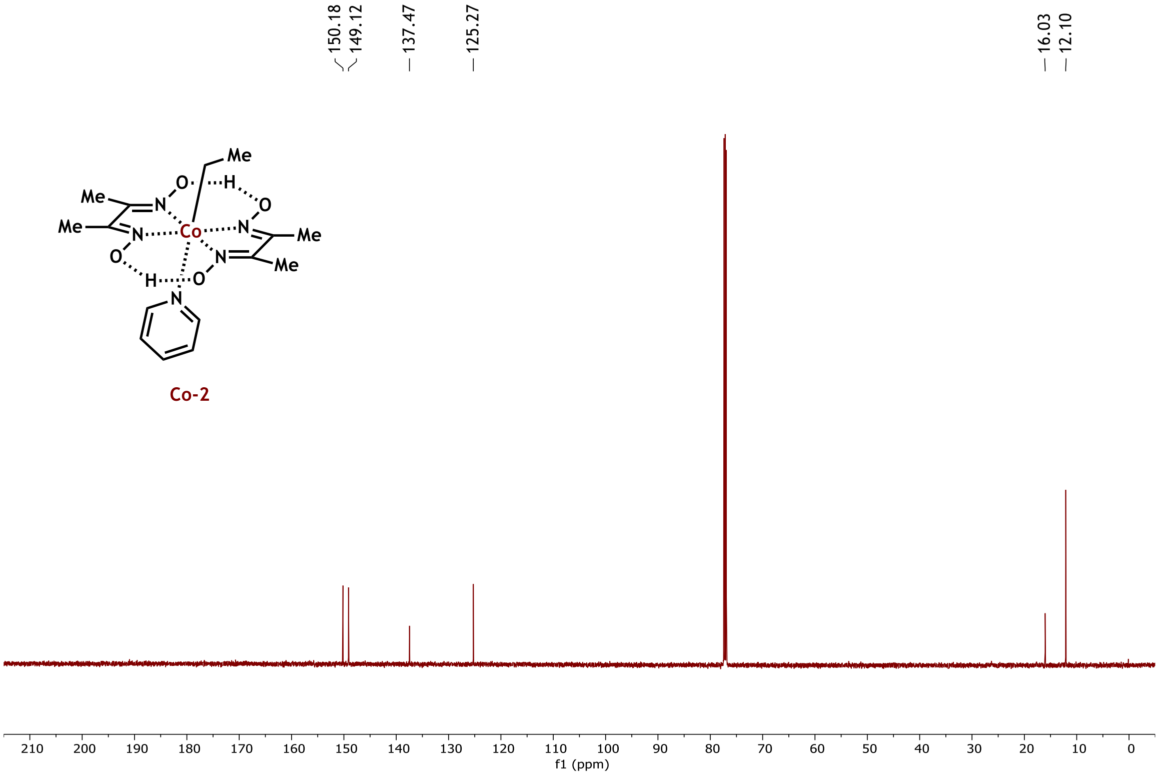

Figure S12. ^{13}C NMR (151 MHz, $CDCl_3$) of [Co(dmgH)$_2$(pyr)Et] (**Co-2**)

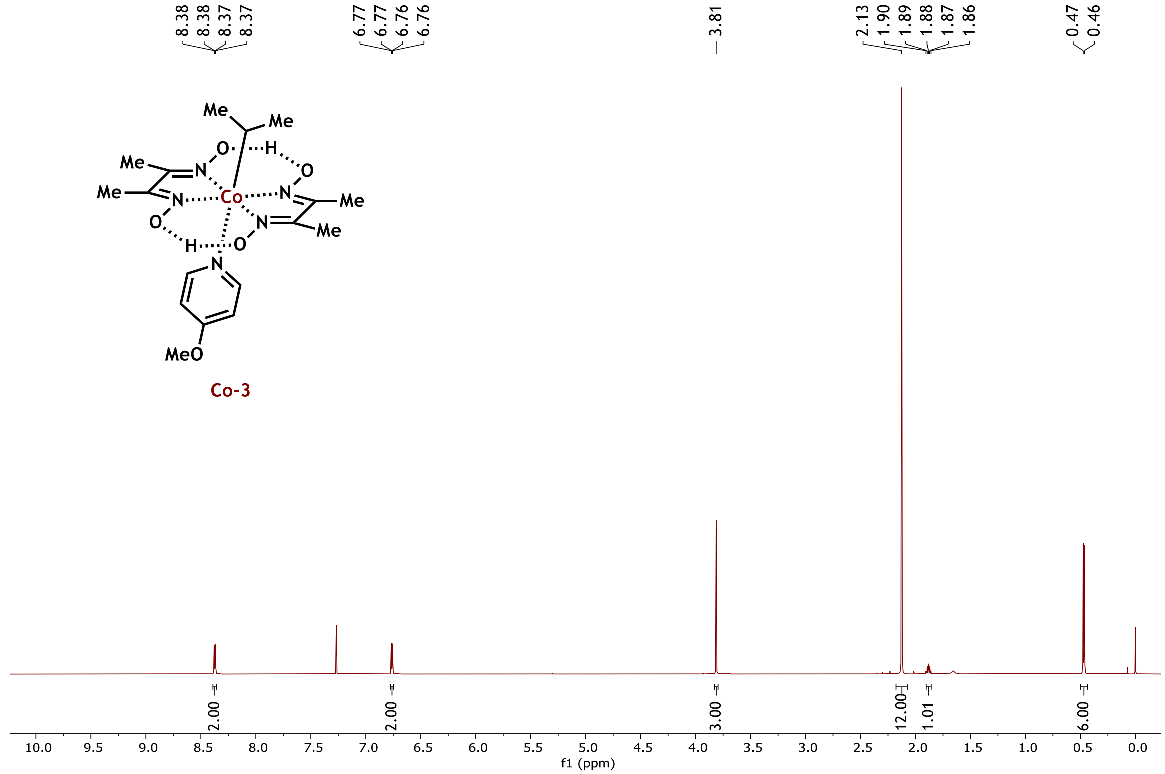

Figure S13. ^{1}H NMR (600 MHz, $CDCl_3$) of [Co(dmgH)$_2$(*p*-methoxypyridine)*i*-Pr] (**Co-3**)

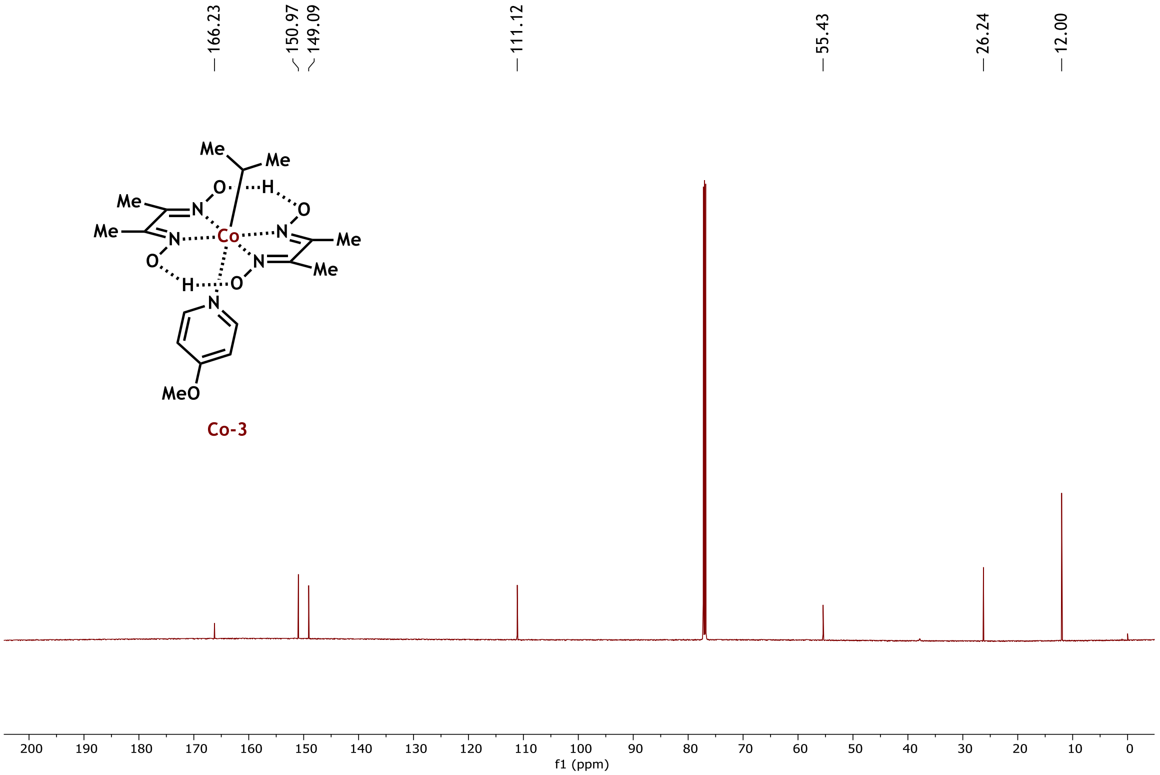

Figure S14. ^{13}C NMR (151 MHz, $CDCl_3$) of [Co(dmgH)$_2$(*p*-methoxypyridine)*i*-Pr] (**Co-3**)

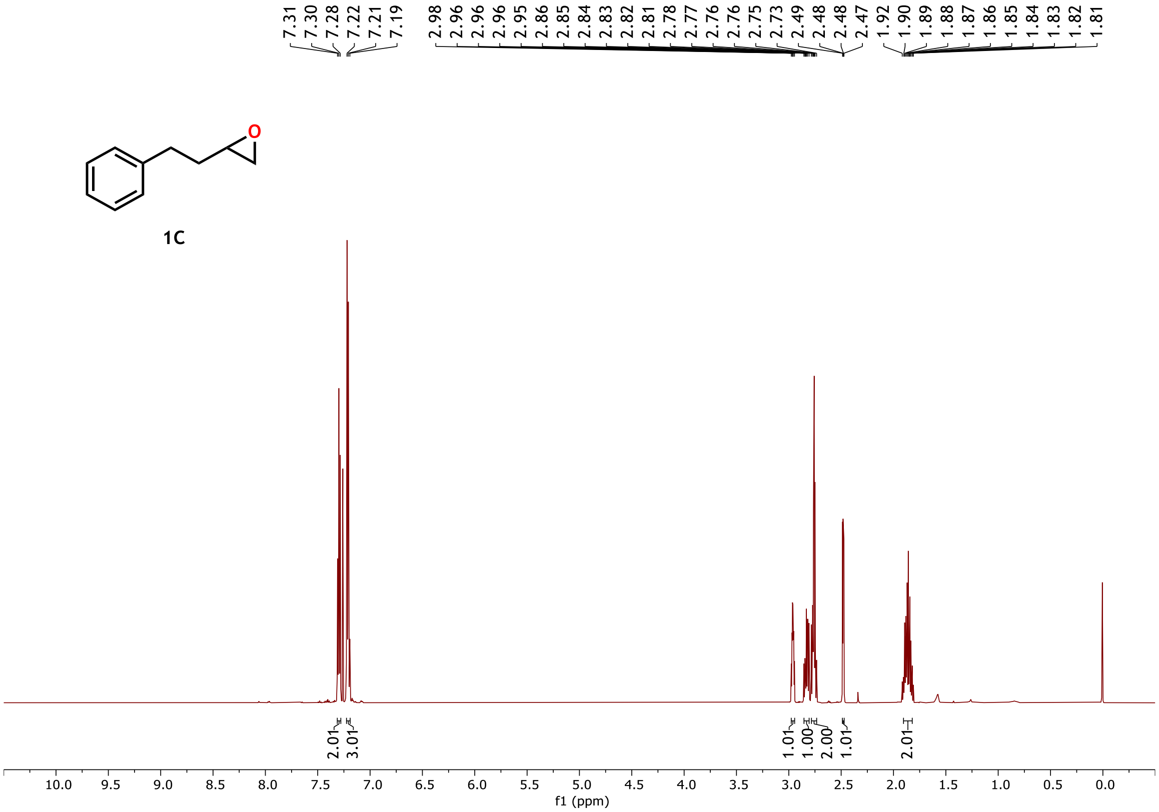

Figure S15. ^{1}H NMR (600 MHz, $CDCl_3$) of 2-phenethyloxirane (**1C**)

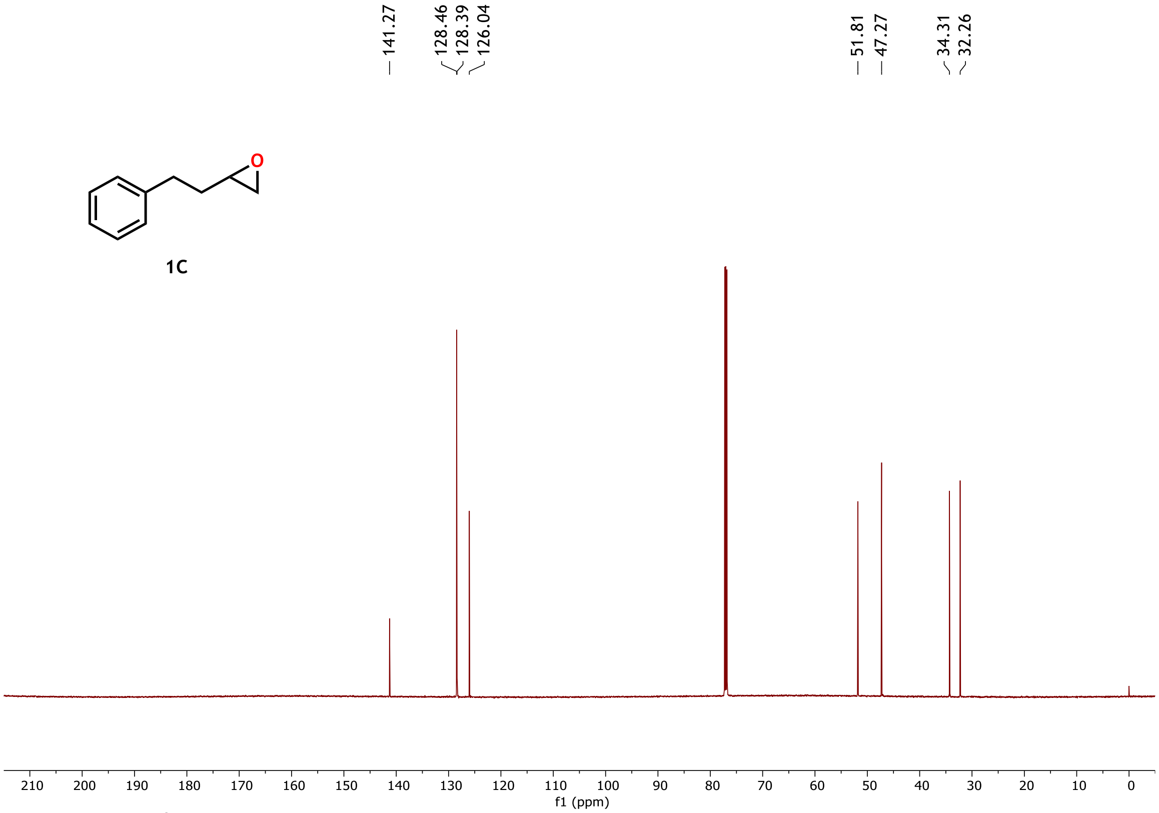

Figure S16. ^{13}C NMR (151 MHz, $CDCl_3$) of 2-phenethyloxirane (**1C**)

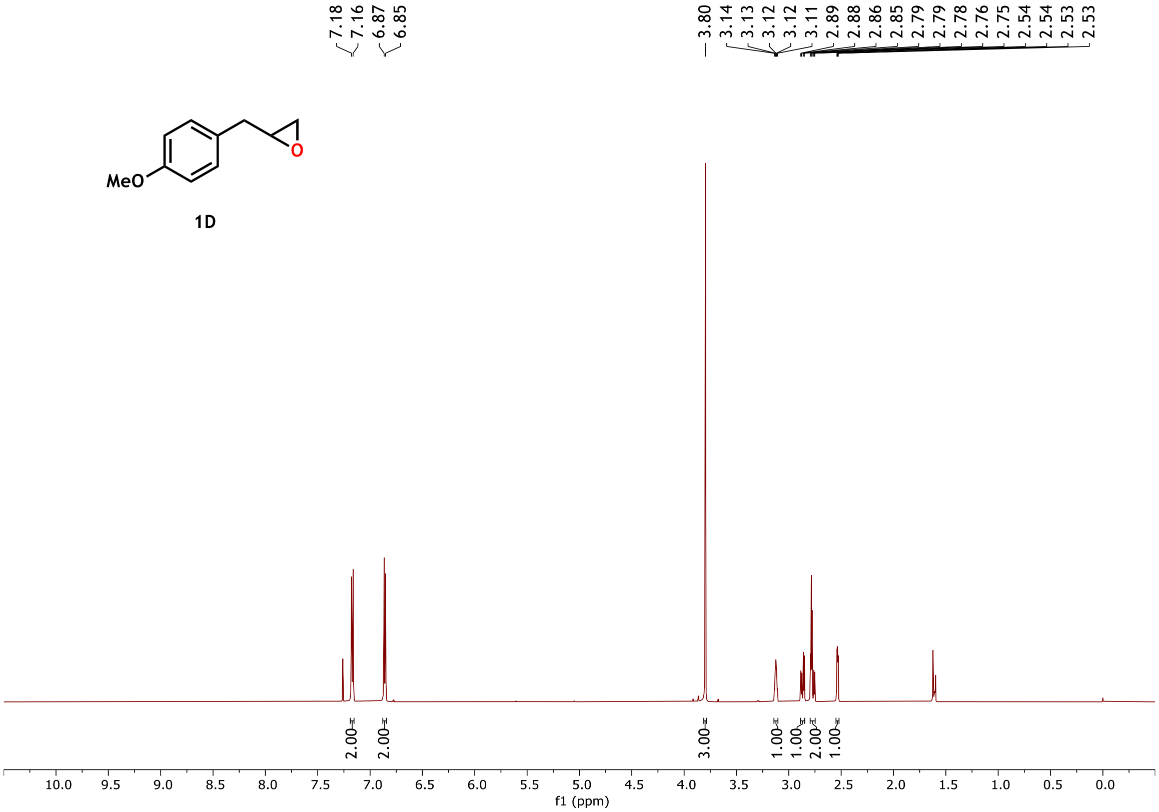

Figure S17. ^{1}H NMR (600 MHz, $CDCl_3$) of 2-(4-methoxybenzyl)oxirane (**1D**)

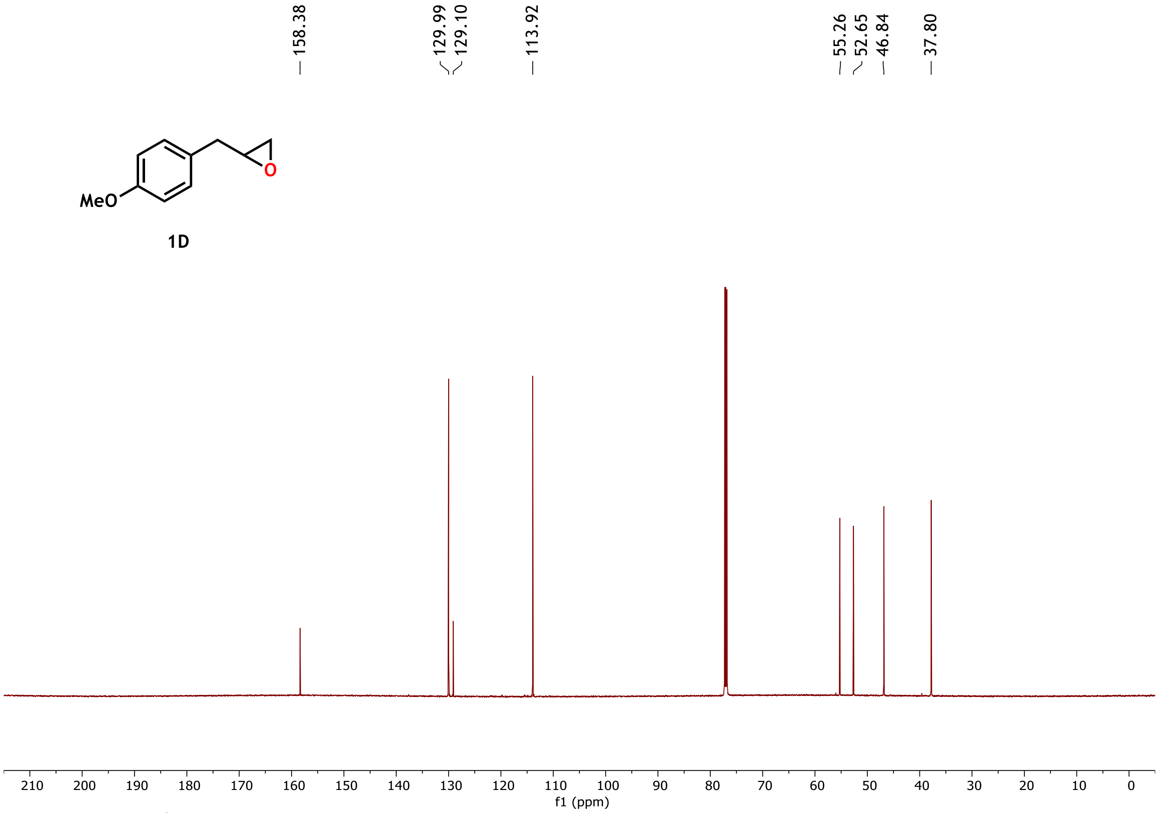

Figure S18. ^{13}C NMR (151 MHz, $CDCl_3$) of 2-(4-methoxybenzyl)oxirane (**1D**)

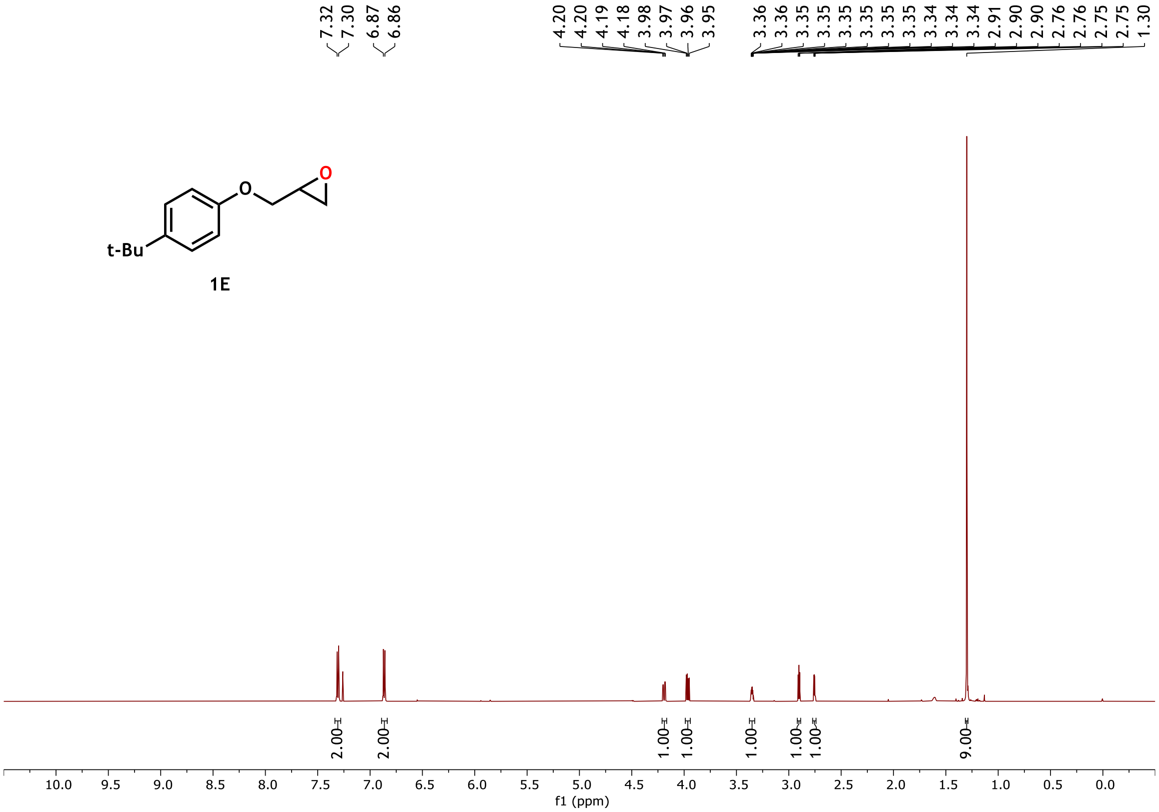

Figure S19. ^{1}H NMR (600 MHz, $CDCl_3$) of 2-((4-(tert-butyl)phenoxy)methyl)oxirane (**1E**)

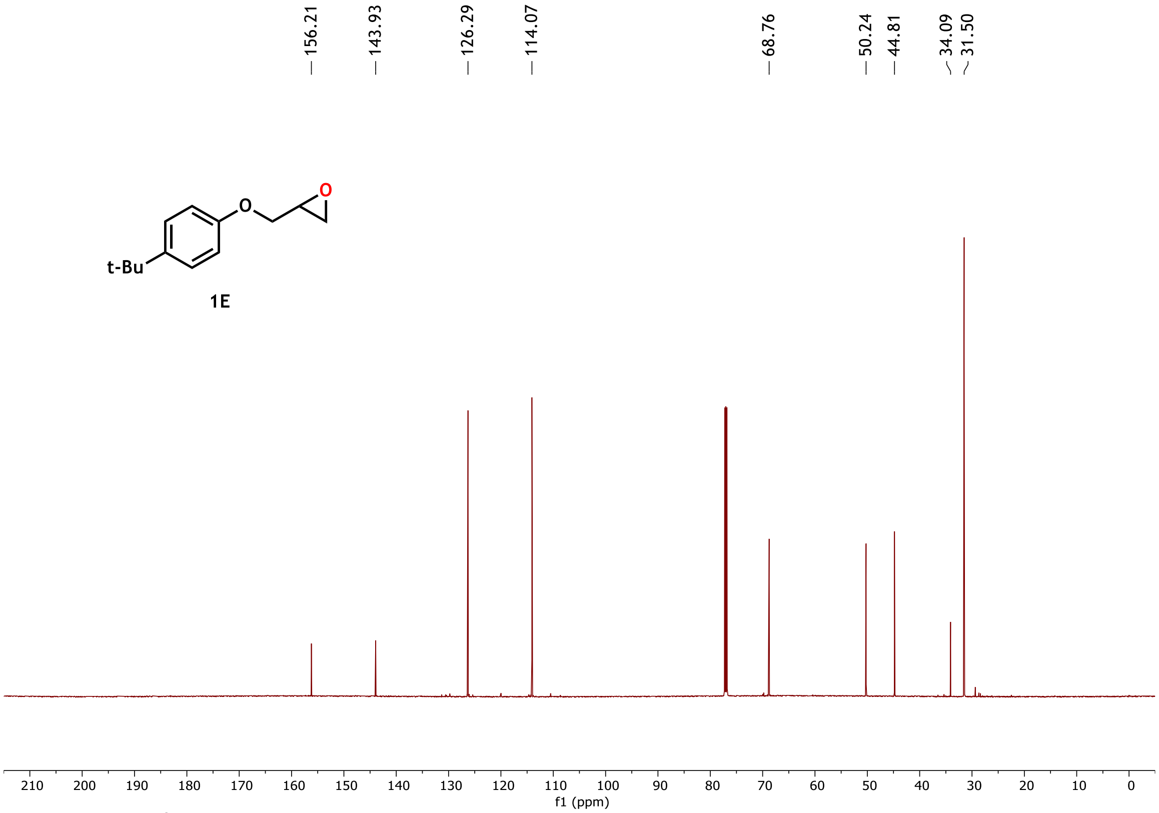

Figure S20. ^{13}C NMR (151 MHz, $CDCl_3$) of 2-((4-(tert-butyl)phenoxy)methyl)oxirane (**1E**)

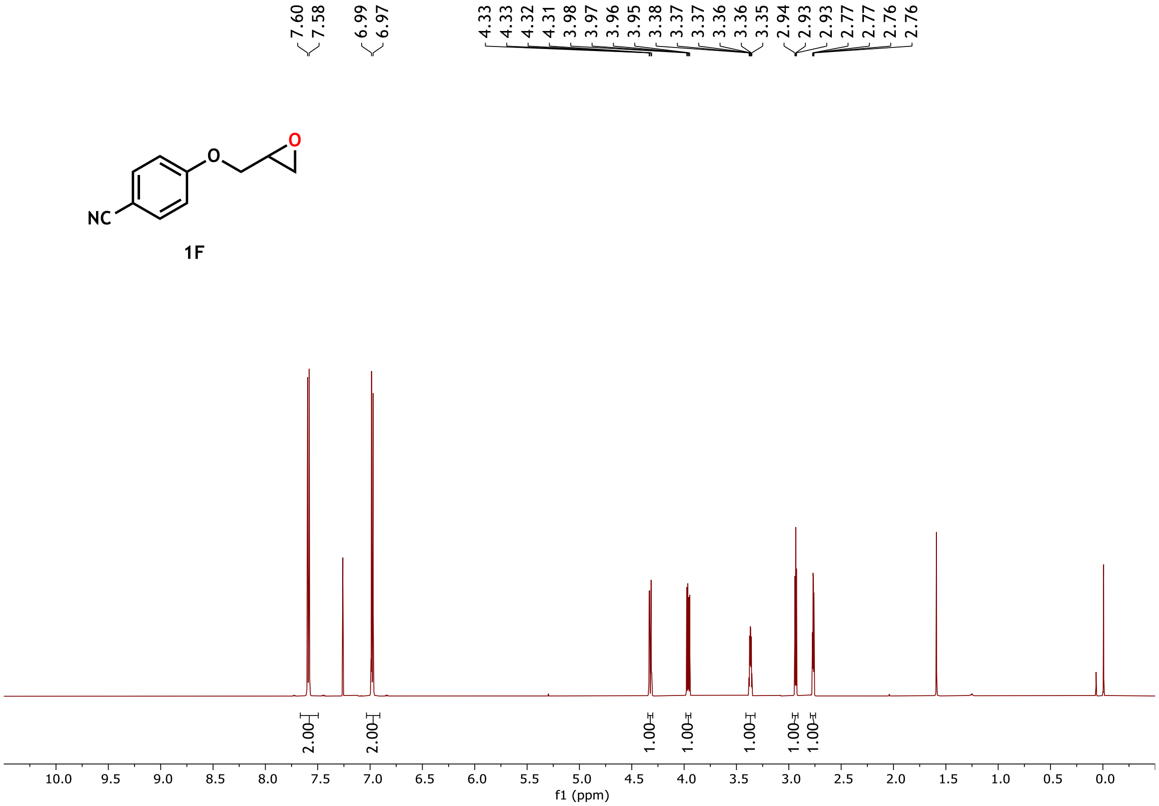

Figure S21. ^{1}H NMR (600 MHz, $CDCl_3$) of 4-(oxiran-2-ylmethoxy)benzonitrile (**1F**)

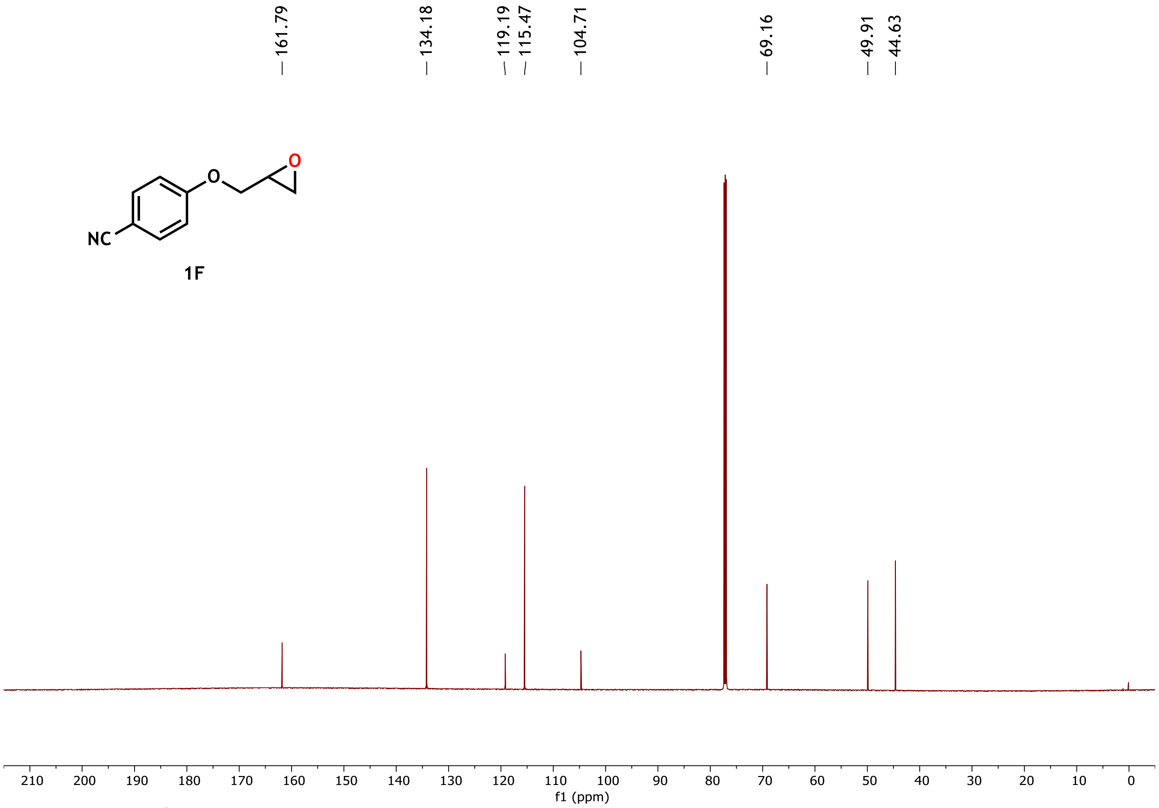

Figure S22. ^{13}C NMR (151 MHz, $CDCl_3$) of 4-(oxiran-2-ylmethoxy)benzonitrile (**1F**)

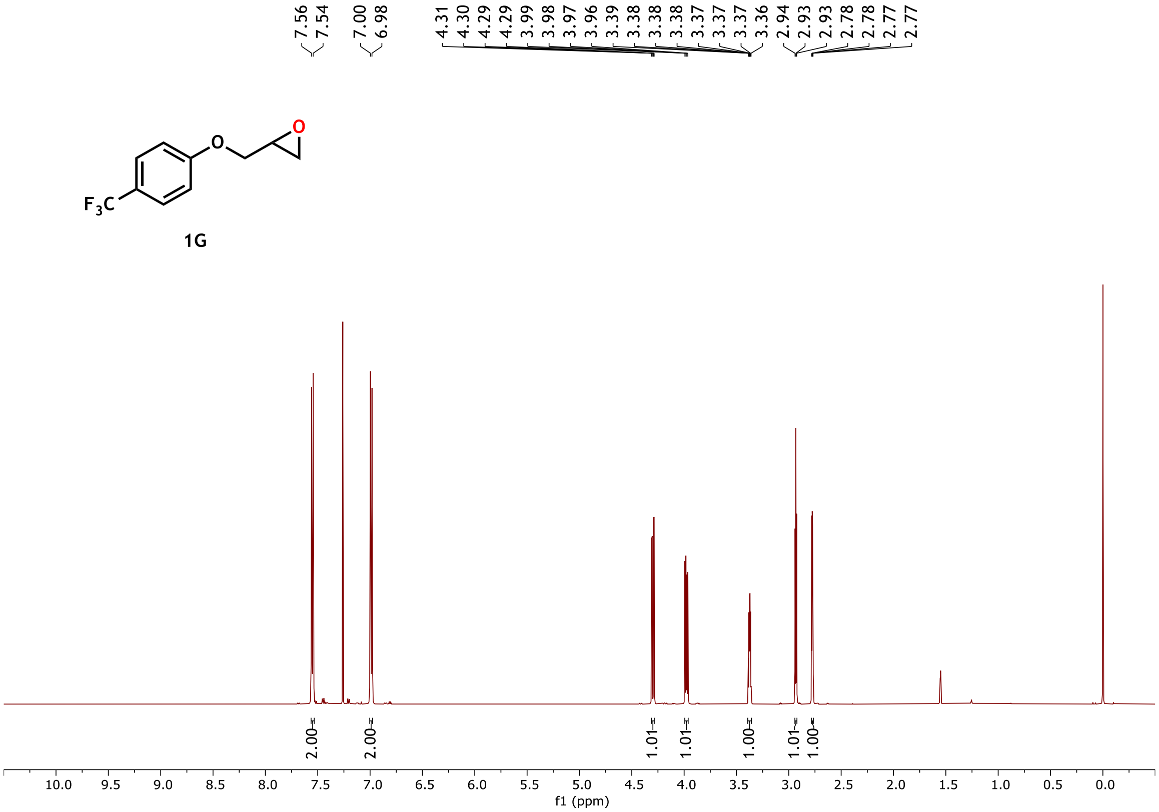

Figure S23. ^{1}H NMR (600 MHz, $CDCl_3$) of 2-((4-(trifluoromethyl)phenoxy)methyl)oxirane (**1G**)

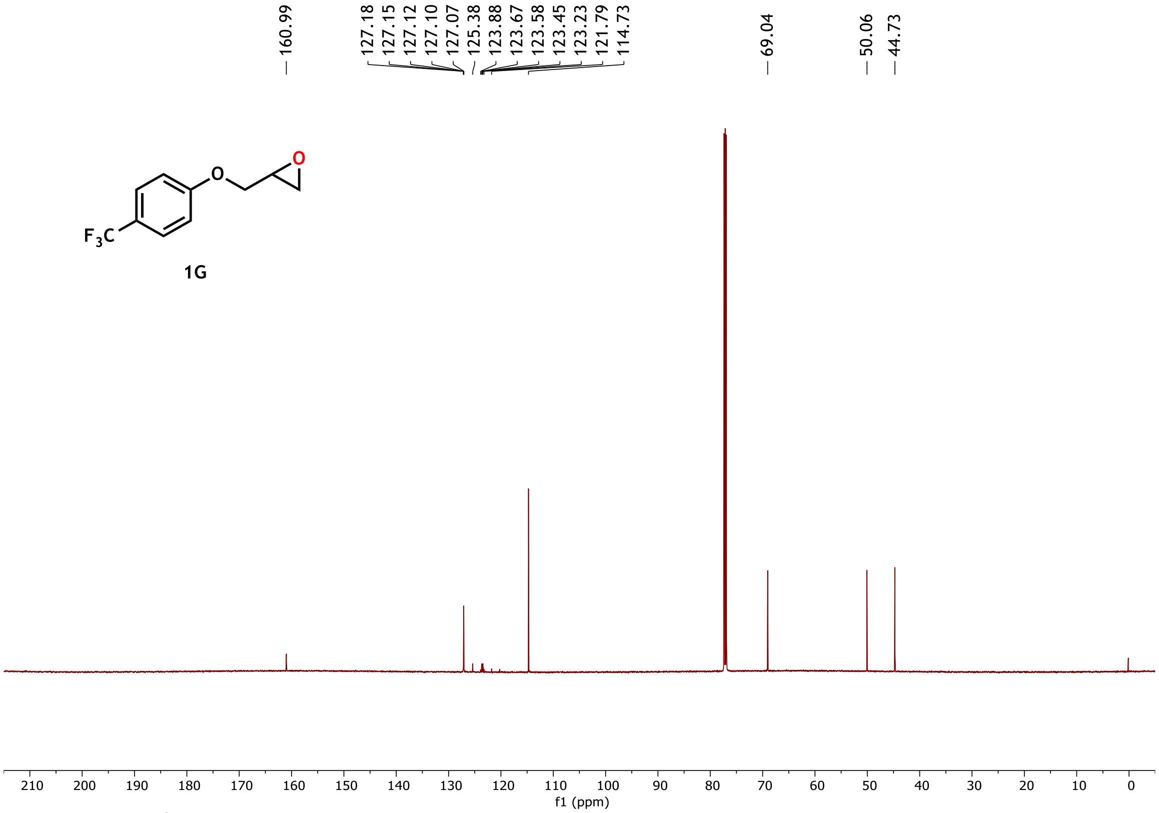

Figure S24. ^{13}C NMR (151 MHz, $CDCl_3$) of 2-((4-(trifluoromethyl)phenoxy)methyl)oxirane (**1G**)

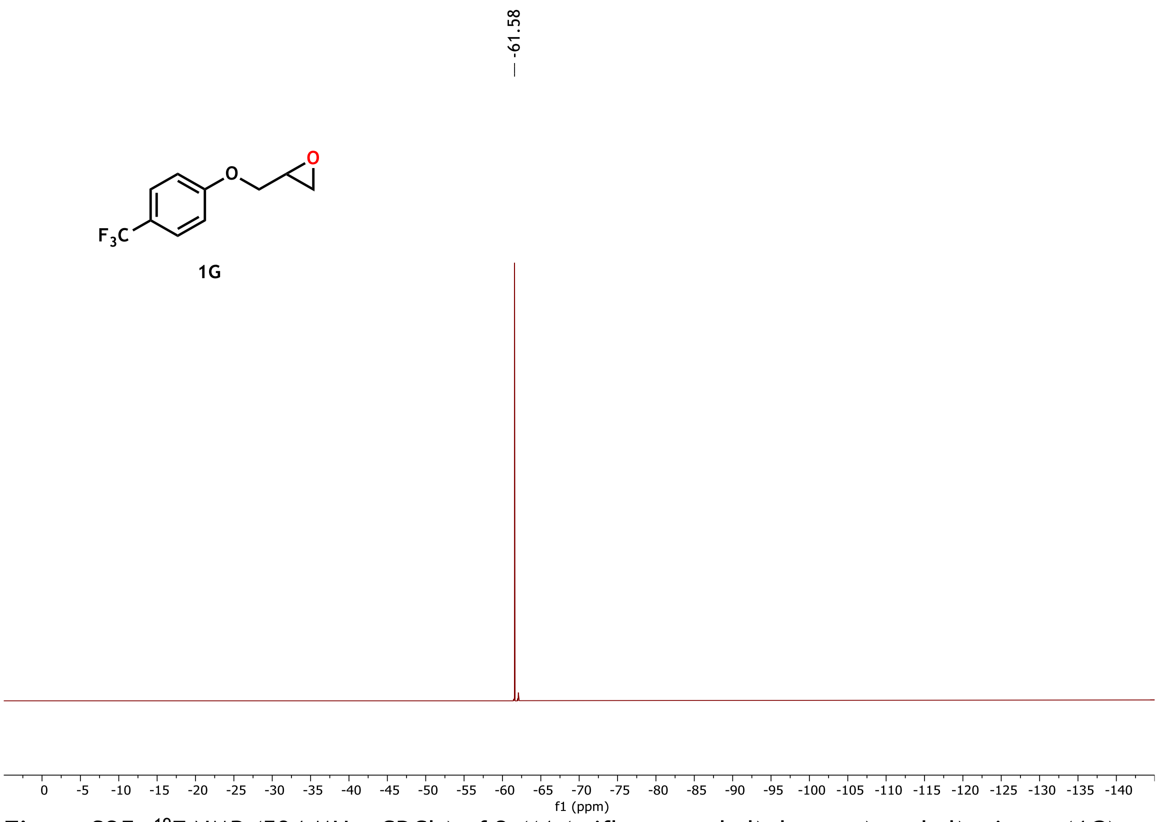

Figure S25. ^{19}F NMR (594 MHz, $CDCl_3$) of 2-((4-(trifluoromethyl)phenoxy)methyl)oxirane (**1G**)

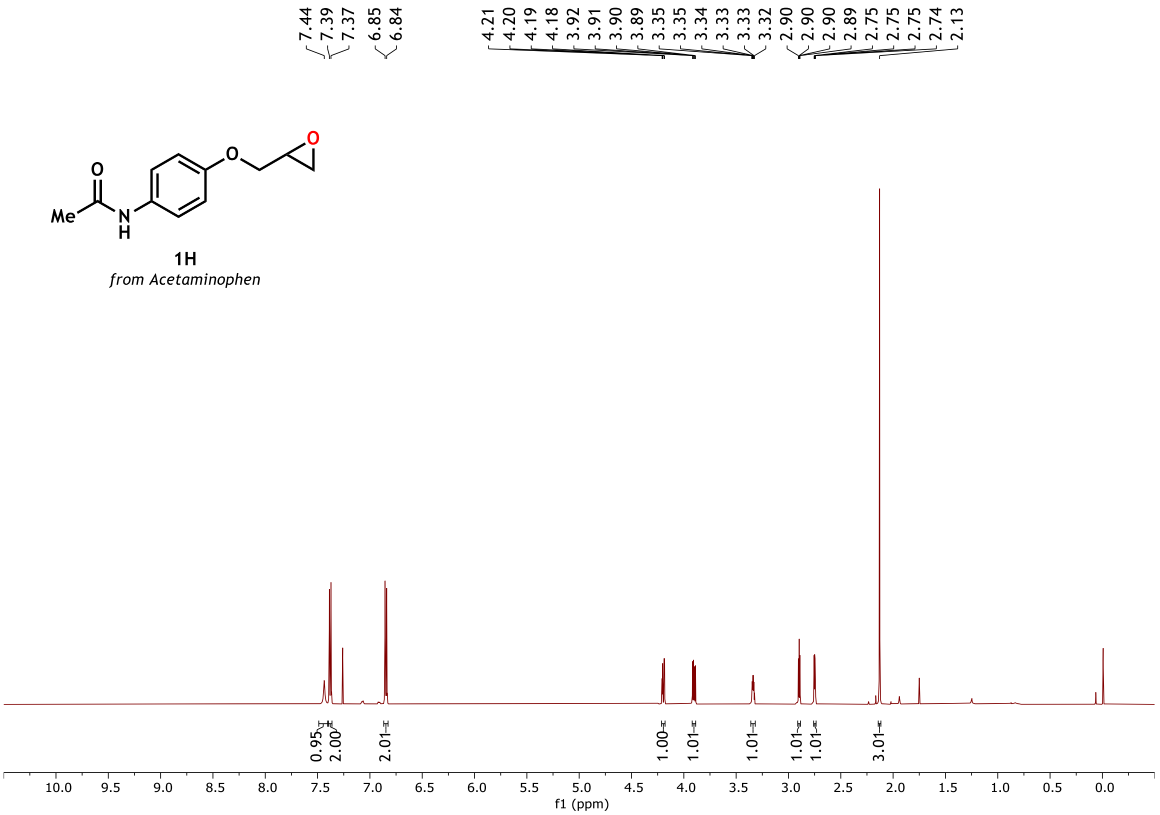

Figure S26. ^{1}H NMR (600 MHz, $CDCl_3$) of N-(4-(oxiran-2-ylmethoxy)phenyl)acetamide (**1H**)

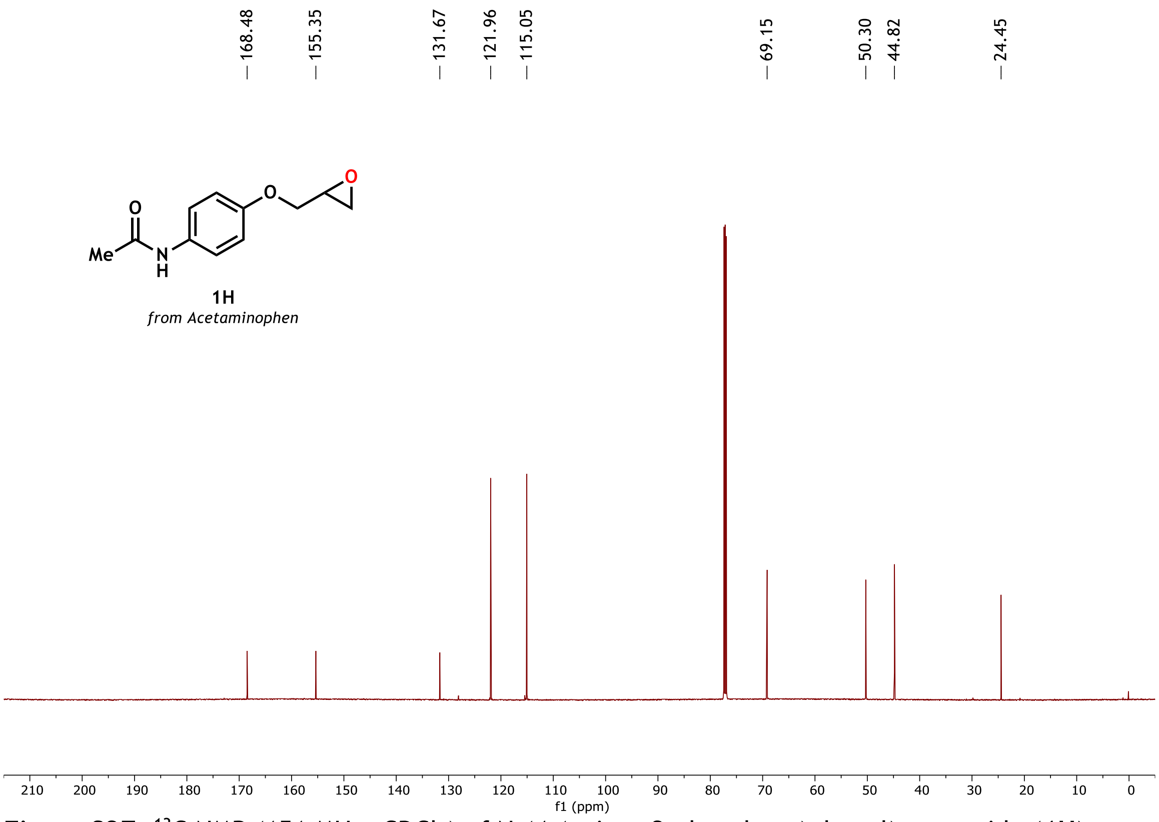

Figure S27. ^{13}C NMR (151 MHz, $CDCl_3$) of N-(4-(oxiran-2-ylmethoxy)phenyl)acetamide (**1H**)

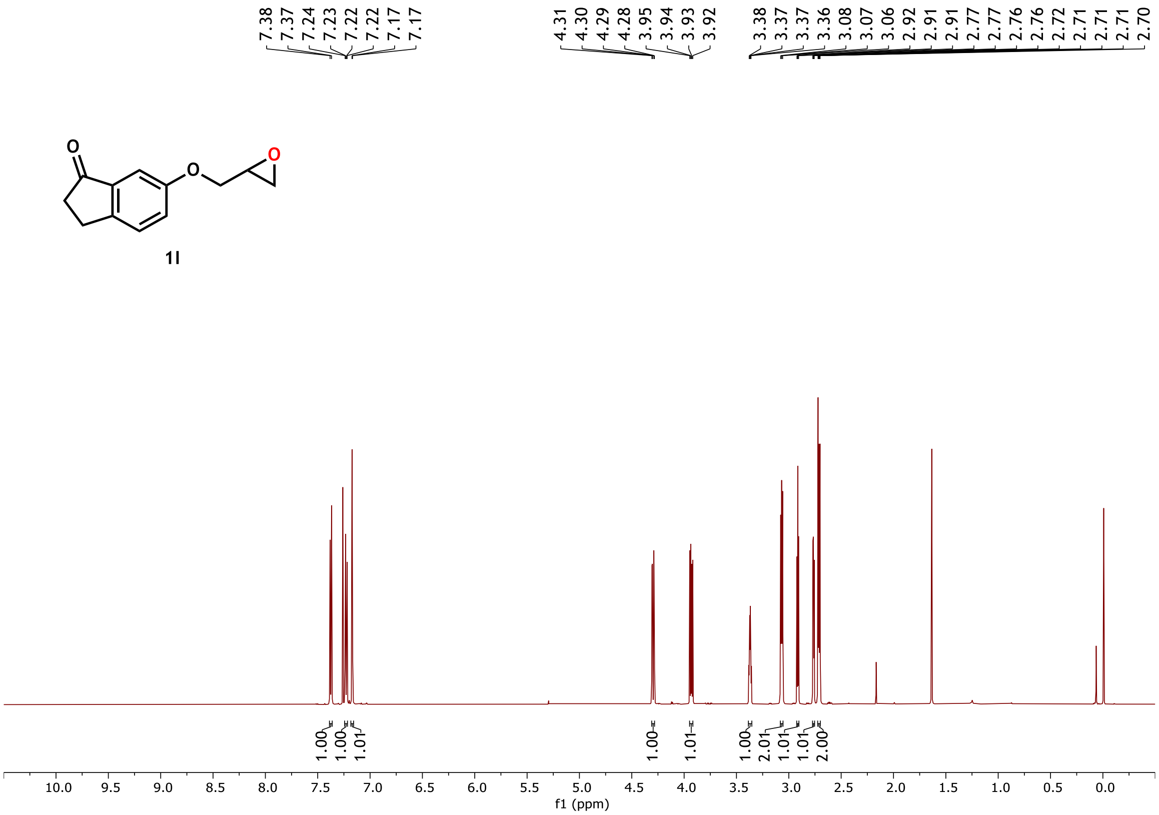

Figure S28. ^{1}H NMR (600 MHz, $CDCl_3$) of 6-(oxiran-2-ylmethoxy)-2,3-dihydro-1H-inden-1-one (**1I**)

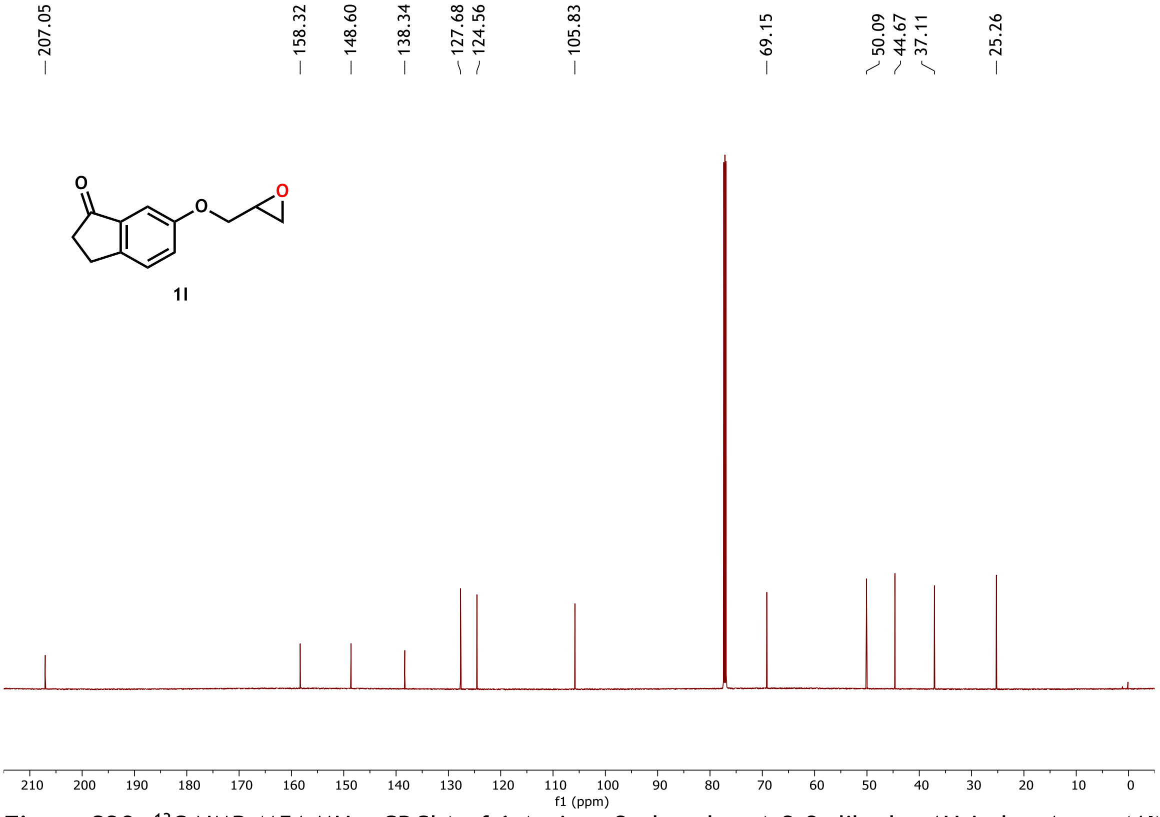

Figure S29. ^{13}C NMR (151 MHz, $CDCl_3$) of 6-(oxiran-2-ylmethoxy)-2,3-dihydro-1H-inden-1-one (**1l**)

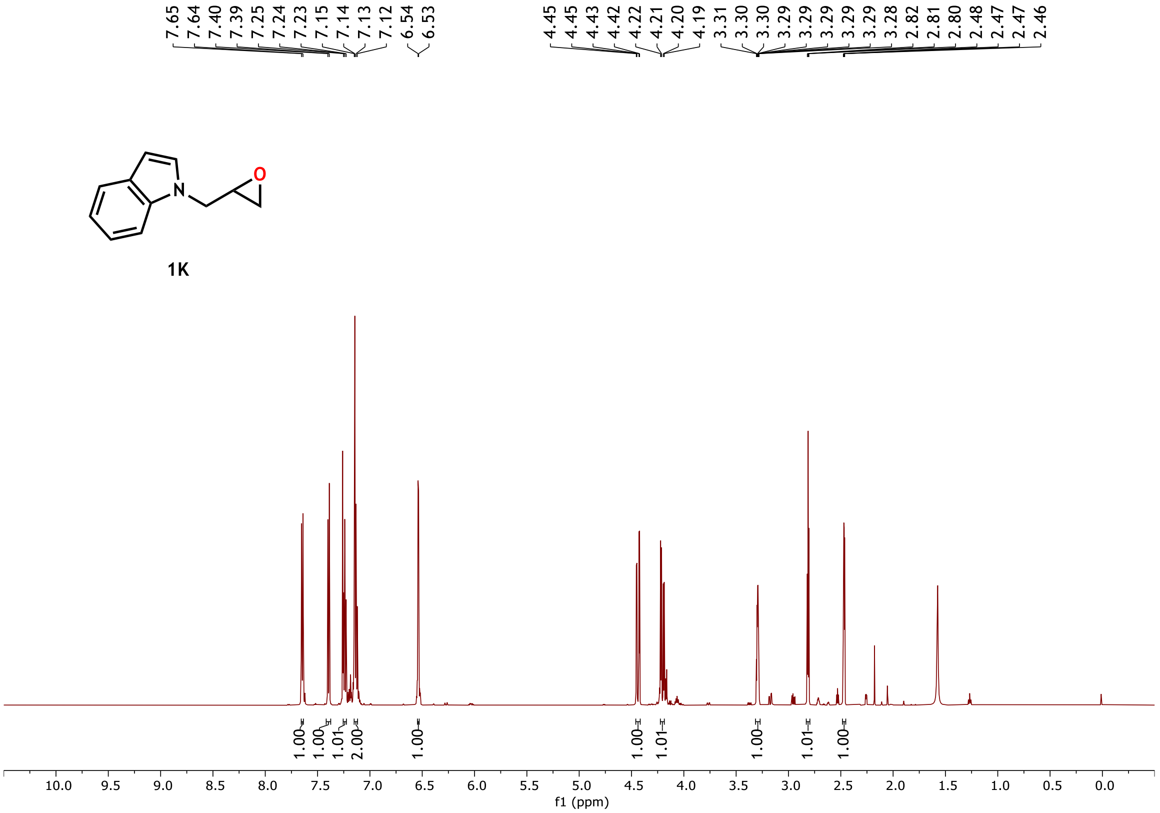

Figure S30. ^{1}H NMR (600 MHz, $CDCl_3$) of 1-(oxiran-2-ylmethyl)-1H-indole (**1K**)

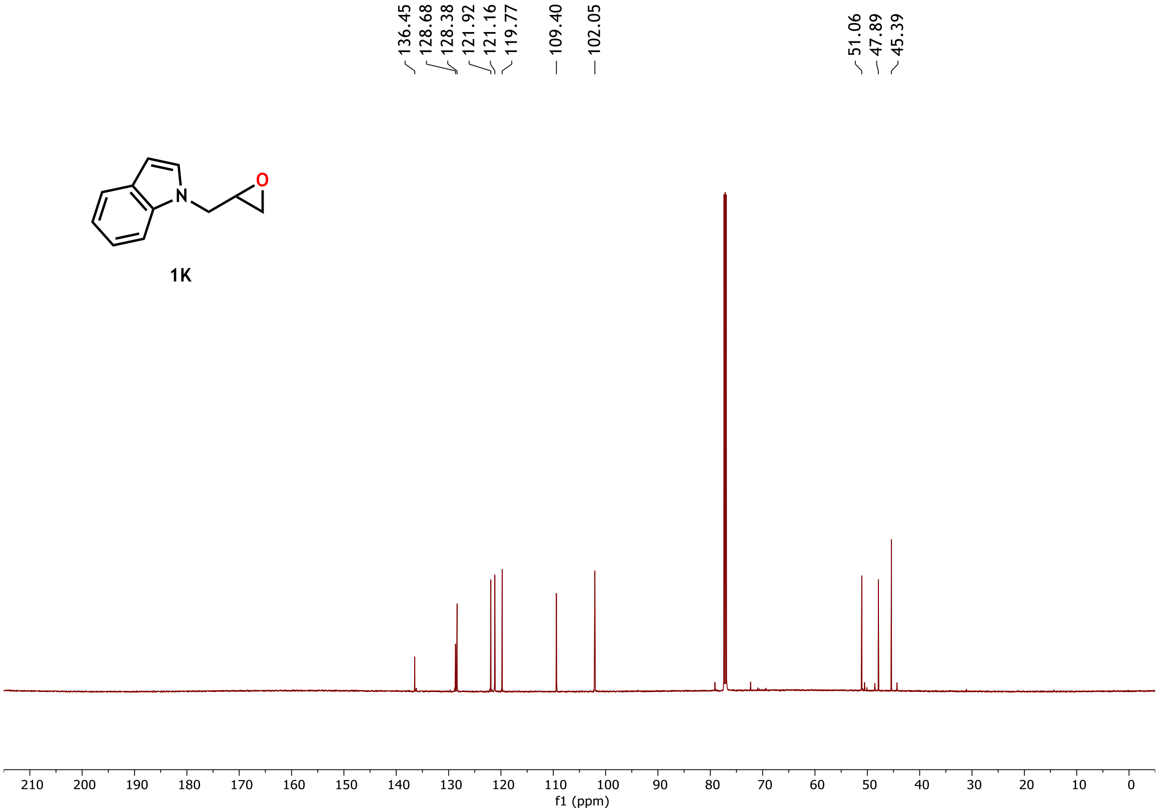

Figure S31. ^{13}C NMR (151 MHz, $CDCl_3$) of 1-(oxiran-2-ylmethyl)-1H-indole (**1K**)

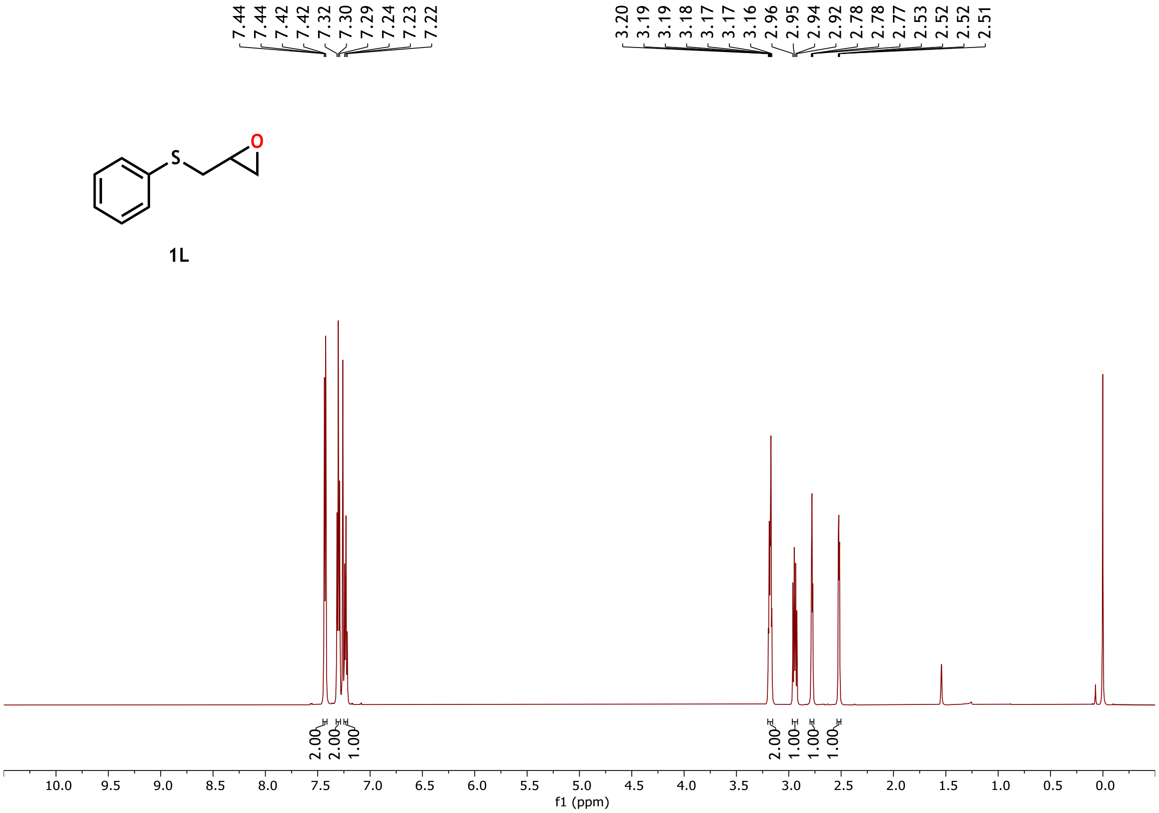

Figure S32. ^{1}H NMR (600 MHz, $CDCl_3$) of 2-((phenylthio)methyl)oxirane (**1L**)

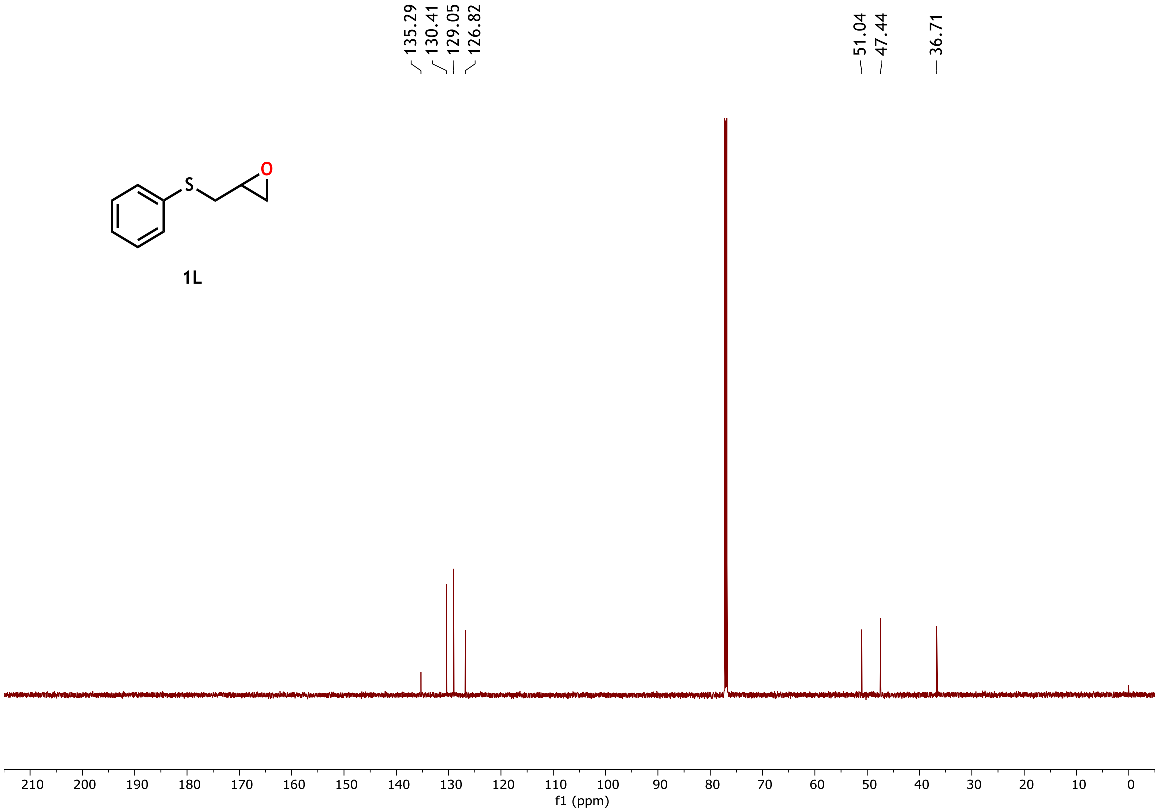

Figure S33. ^{13}C NMR (151 MHz, $CDCl_3$) of 2-((phenylthio)methyl)oxirane (**1L**)

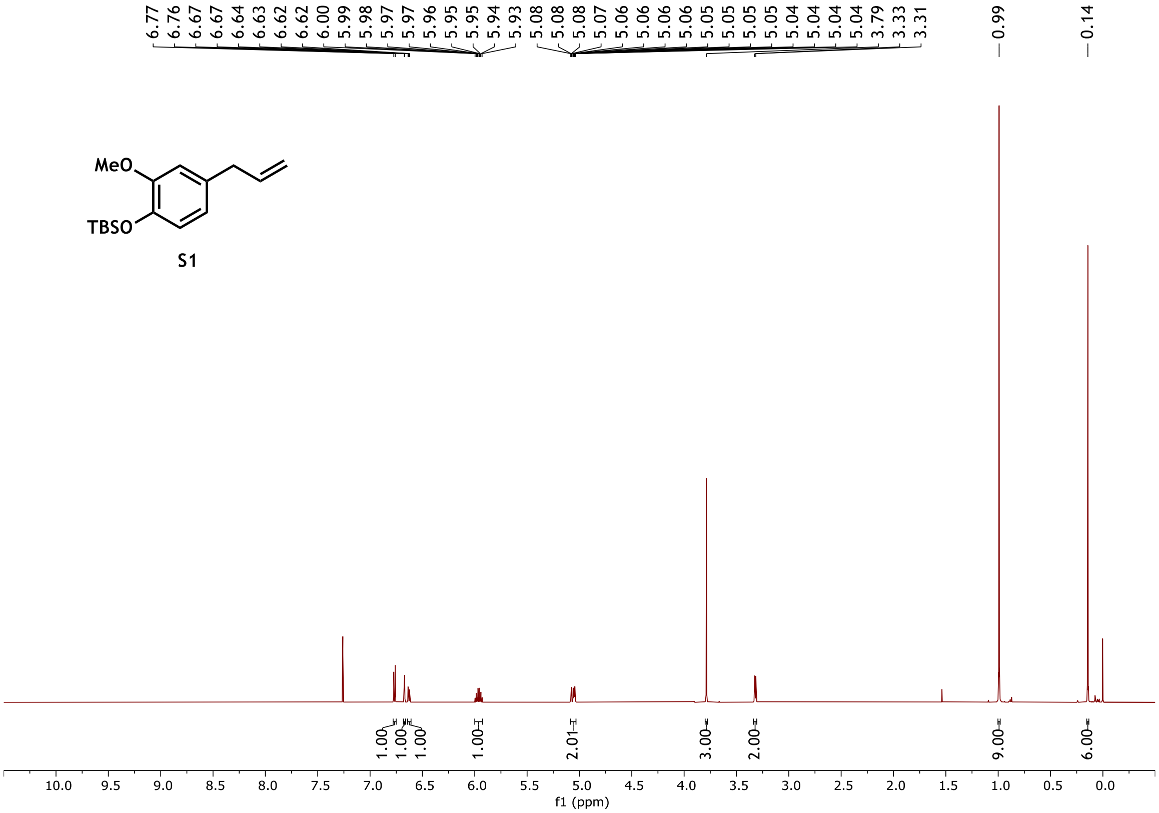

Figure S34. ^{1}H NMR (600 MHz, $CDCl_3$) of (4-allyl-2-methoxyphenoxy)(tert-butyl)dimethylsilane (**S1**)

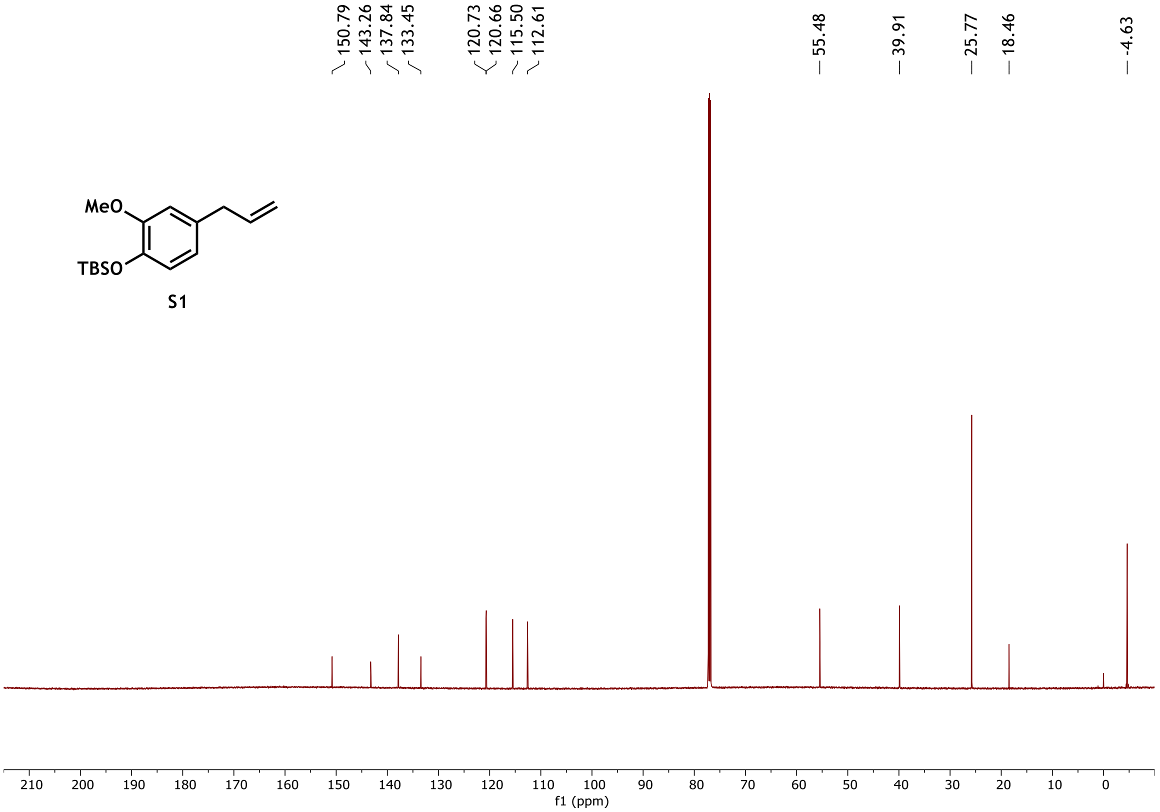

Figure S35. ^{13}C NMR (151 MHz, $CDCl_3$) of (4-allyl-2-methoxyphenoxy)(tert-butyl)dimethylsilane (**S1**)

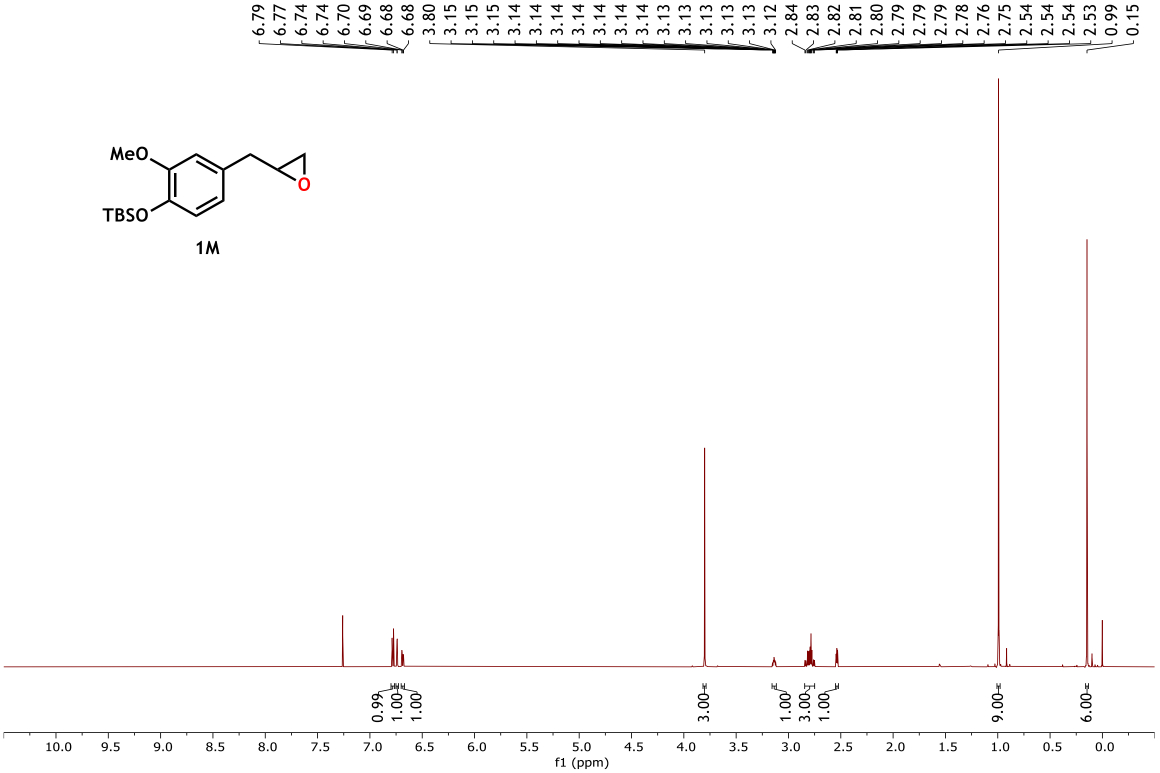

Figure S36. ^{1}H NMR (600 MHz, $CDCl_3$) of tert-butyl(2-methoxy-4-(oxiran-2-ylmethyl)phenoxy)-dimethylsilane (**1M**)

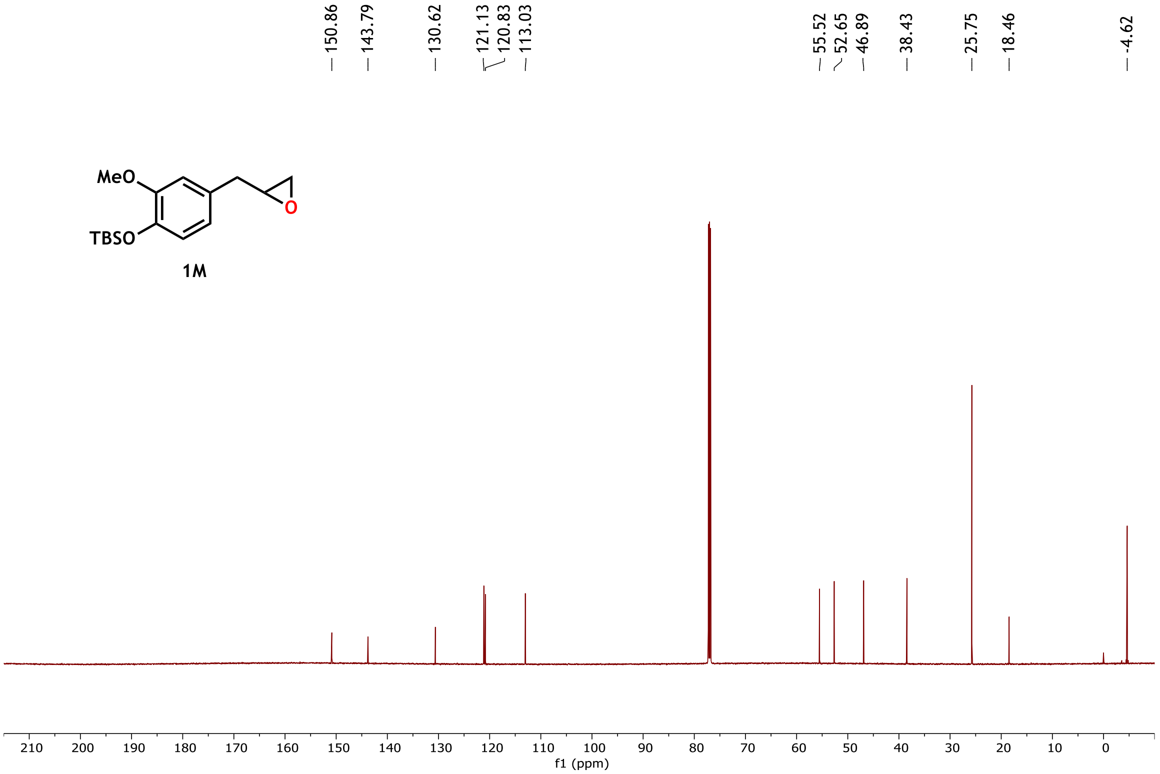

Figure S37. ^{13}C NMR (151 MHz, $CDCl_3$) of tert-butyl(2-methoxy-4-(oxiran-2-ylmethyl)phenoxy)-dimethylsilane (**1M**)

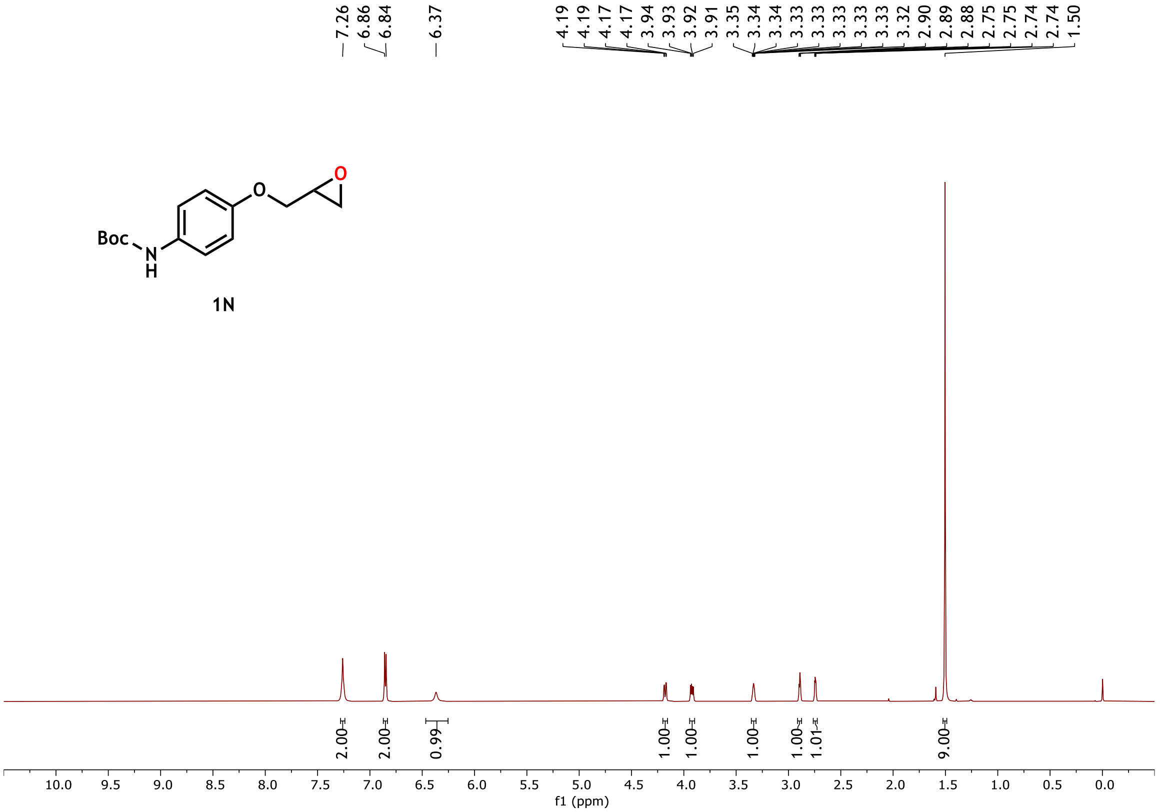

Figure S38. ^{1}H NMR (600 MHz, $CDCl_3$) of tert-butyl (4-(oxiran-2-ylmethoxy)phenyl)carbamate (**1N**)

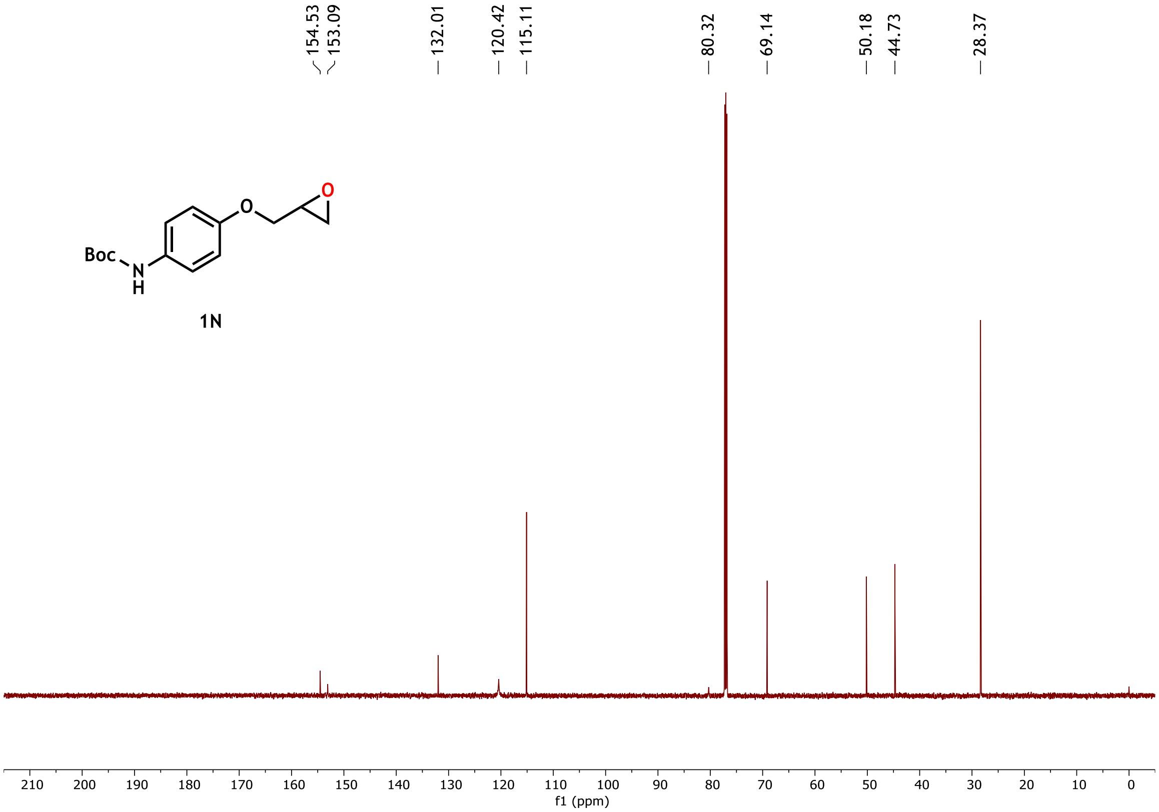

Figure S39. ^{13}C NMR (151 MHz, $CDCl_3$) of tert-butyl (4-(oxiran-2-ylmethoxy)phenyl)carbamate (**1N**)

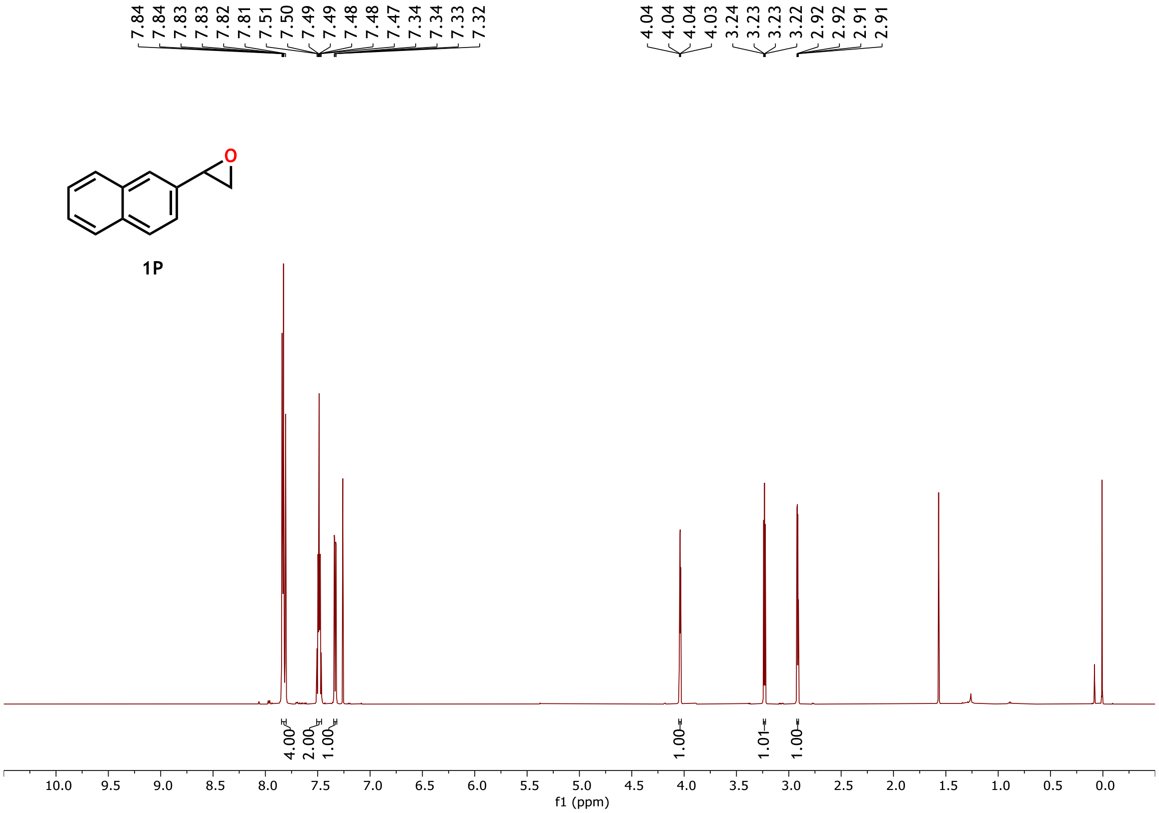

Figure S40. ^{1}H NMR (600 MHz, $CDCl_3$) of 2-(naphthalen-2-yl)oxirane (**1P**)

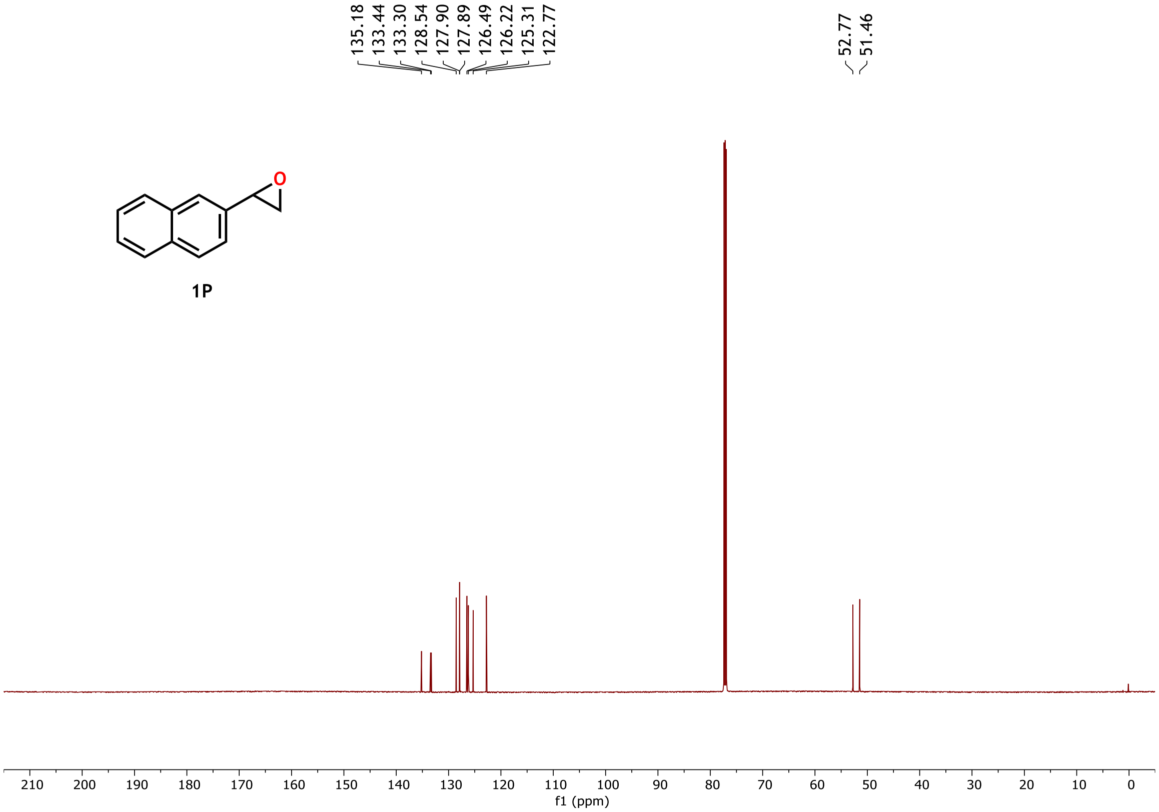

Figure S41. ^{13}C NMR (151 MHz, $CDCl_3$) of 2-(naphthalen-2-yl)oxirane (**1P**)

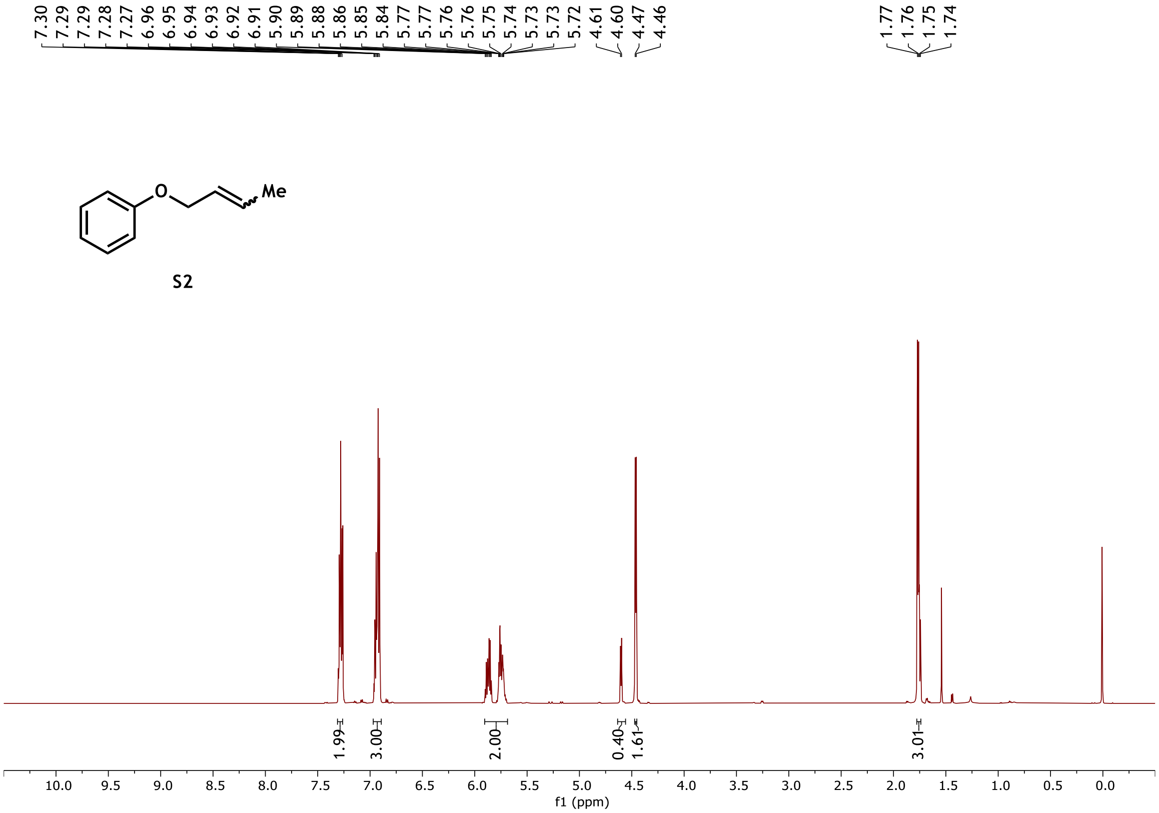

Figure S42. ^{1}H NMR (600 MHz, $CDCl_3$) of (but-2-en-1-yloxy)benzene (**S2**)

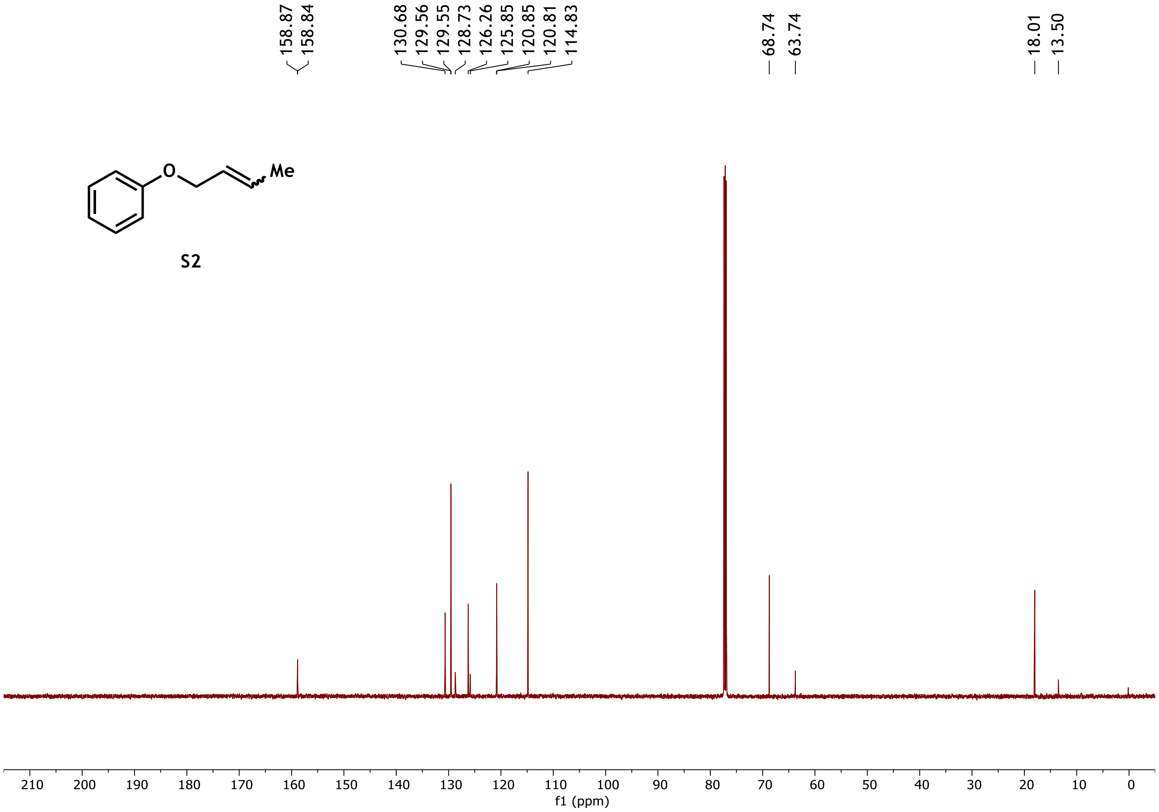

Figure S43. ^{13}C NMR (151 MHz, $CDCl_3$) of (but-2-en-1-yloxy)benzene (**S2**)

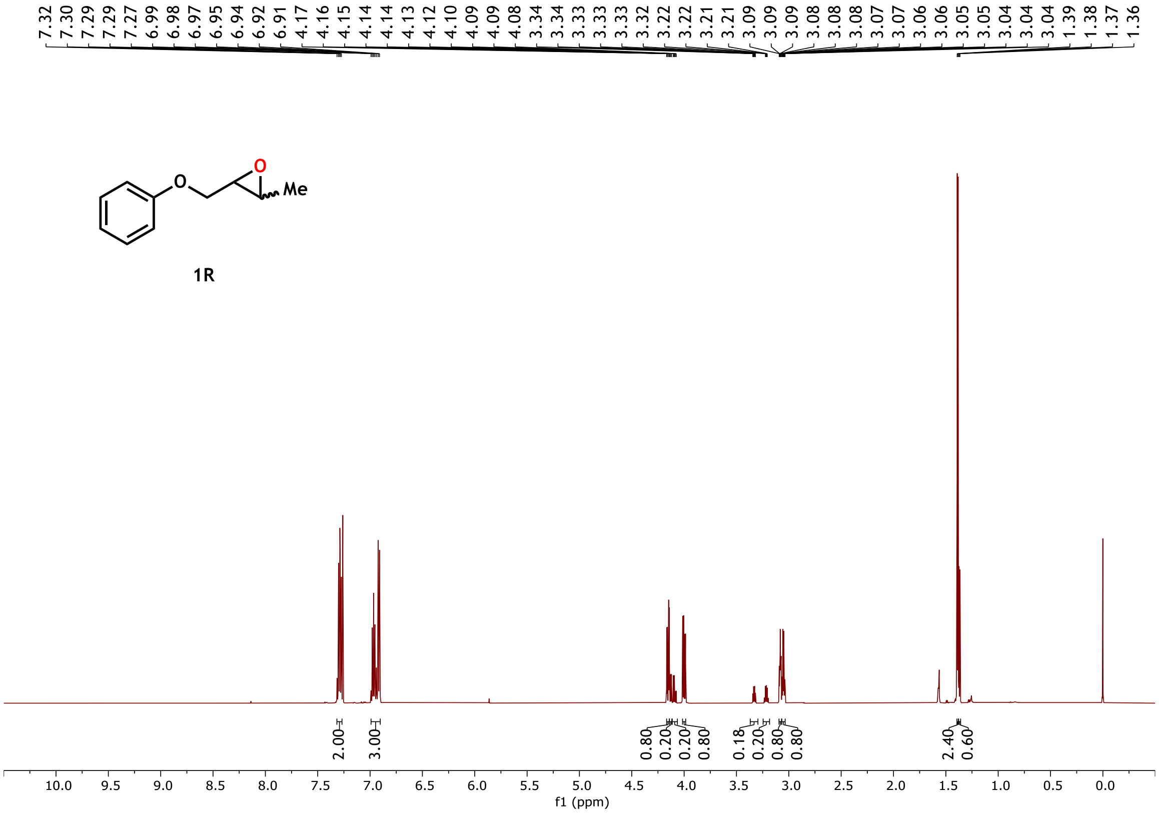

Figure S44. ^{1}H NMR (600 MHz, $CDCl_3$) of 2-methyl-3-(phenoxymethyl)oxirane (**1R**)

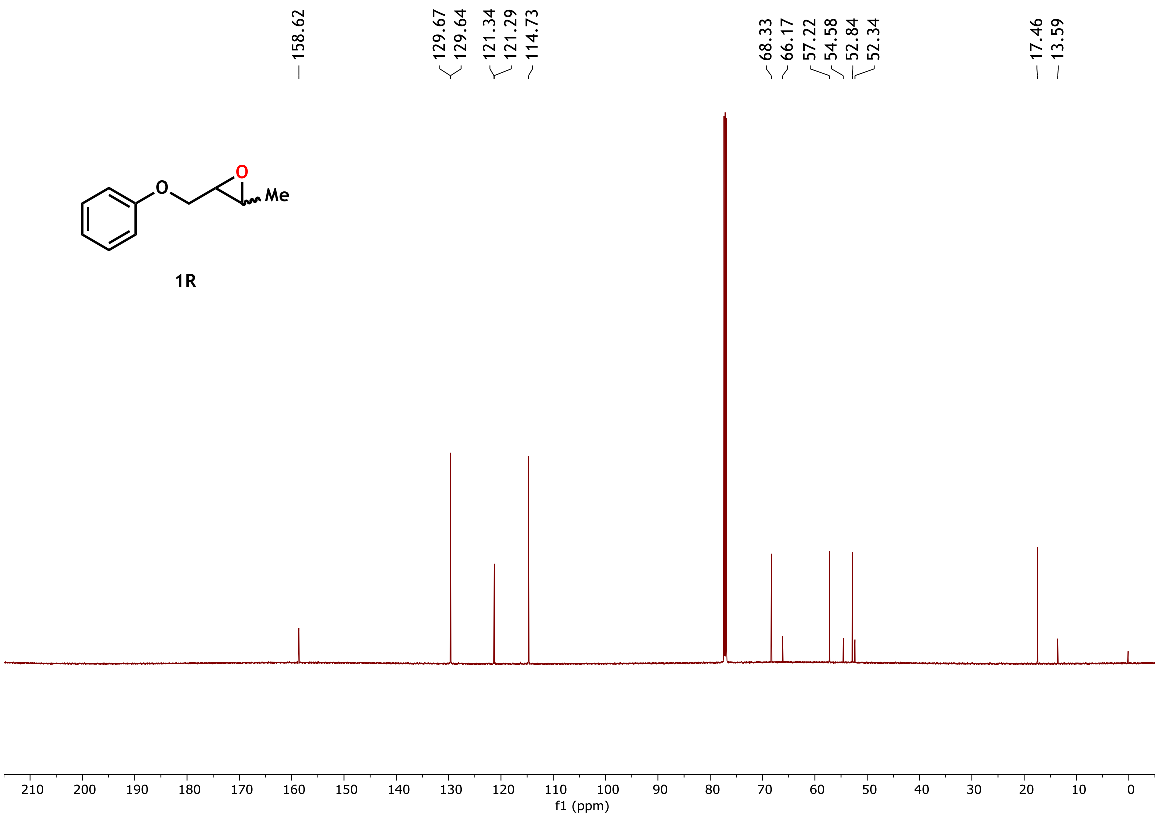

Figure S45. ^{13}C NMR (151 MHz, $CDCl_3$) of 2-methyl-3-(phenoxymethyl)oxirane (**1R**)

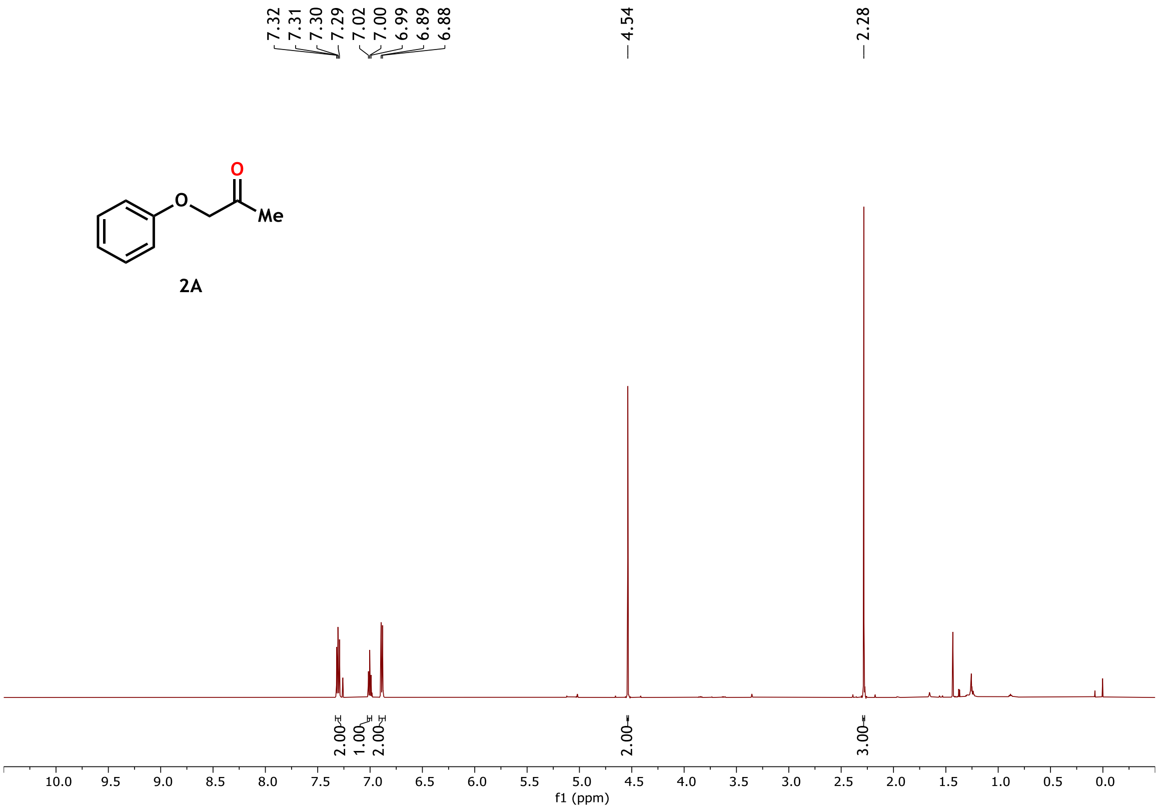

Figure S46. ^{1}H NMR (600 MHz, $CDCl_3$) of 1-phenoxypropan-2-one (**2A**)

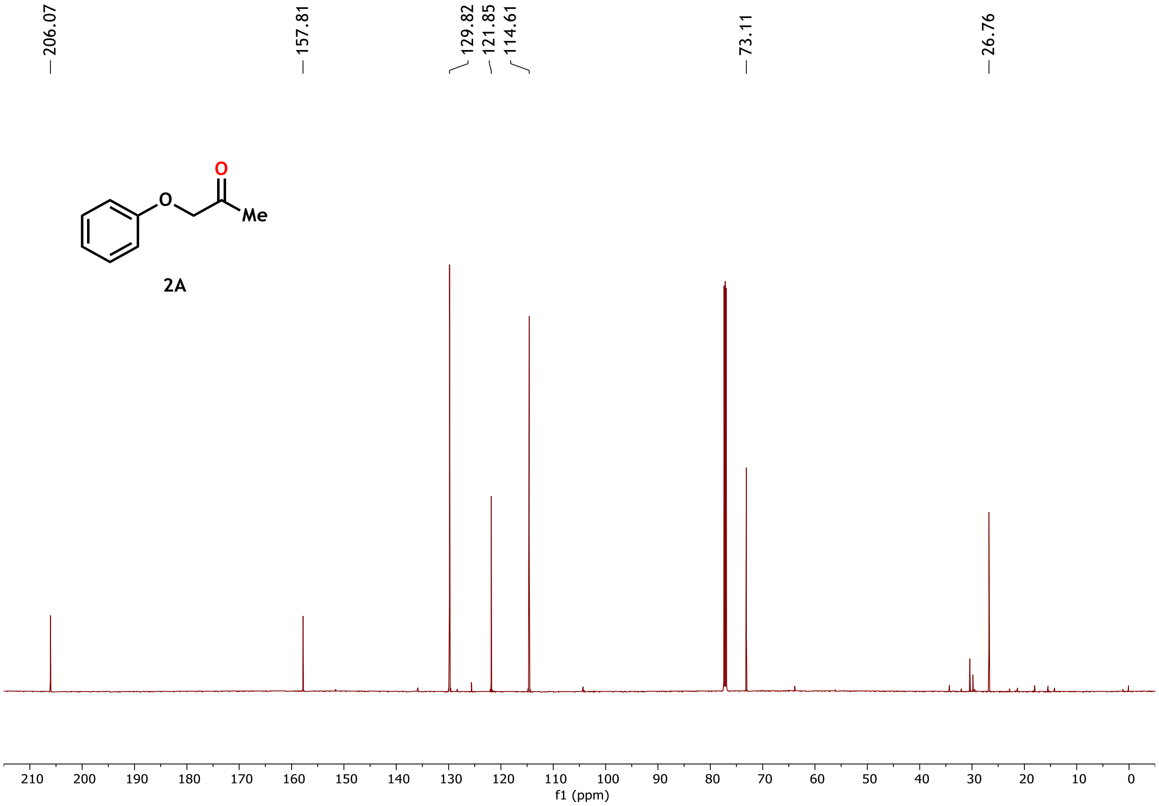

Figure S47. ^{13}C NMR (151 MHz, $CDCl_3$) of 1-phenoxypropan-2-one (**2A**)

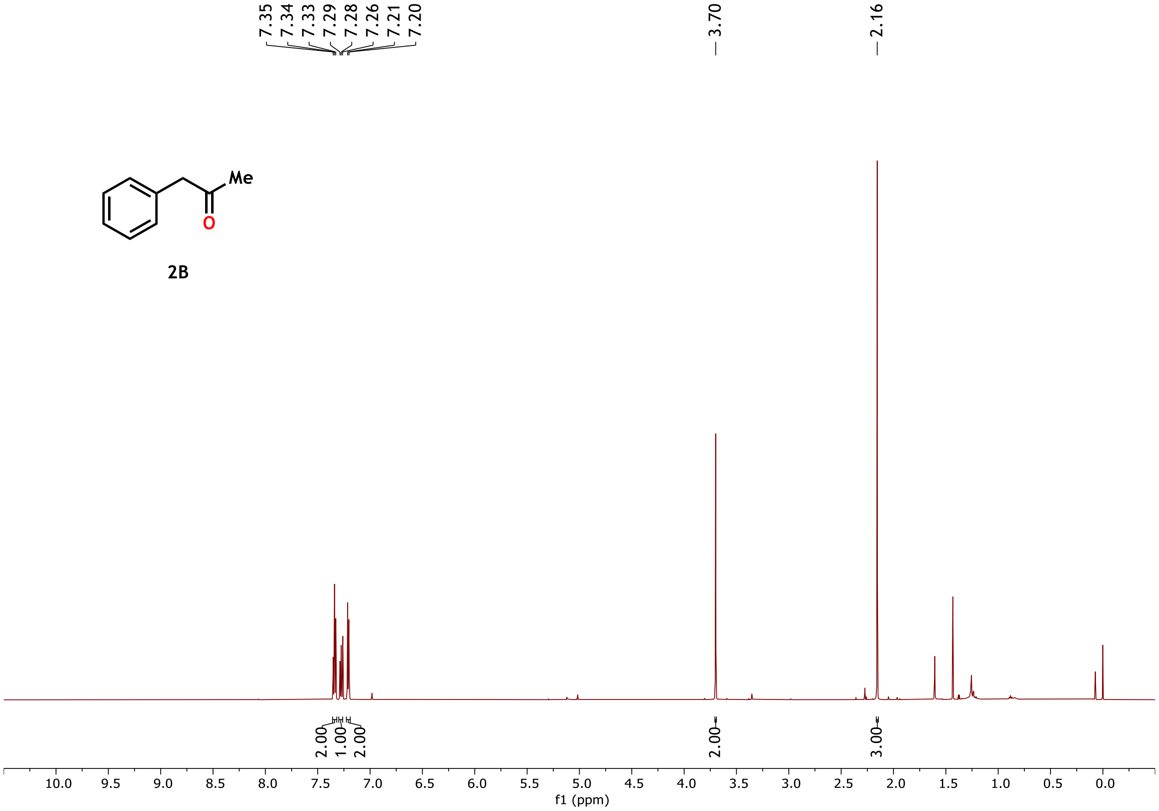

Figure S48. ^{1}H NMR (600 MHz, $CDCl_3$) of 1-phenylpropan-2-one (**2B**)

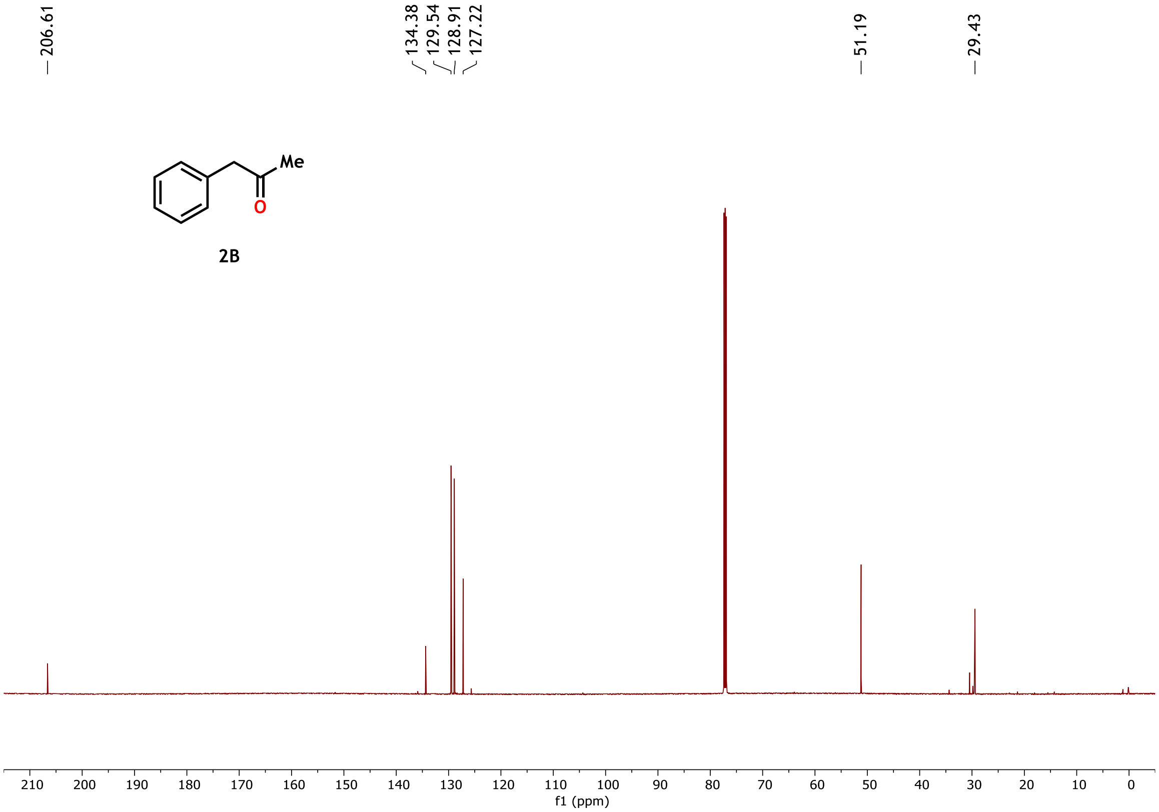

Figure S49. ^{13}C NMR (151 MHz, $CDCl_3$) of 1-phenylpropan-2-one (**2B**)

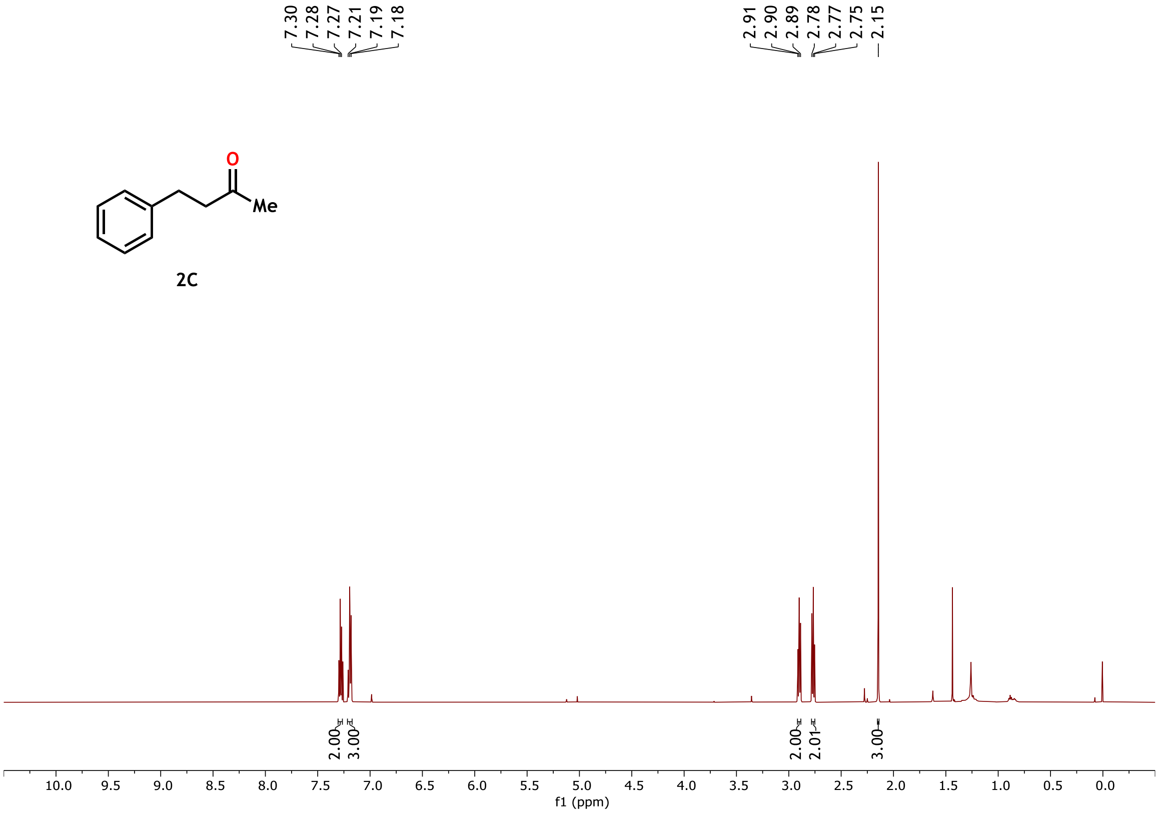

Figure S50. ^{1}H NMR (600 MHz, $CDCl_3$) of 4-phenylbutan-2-one (**2C**)

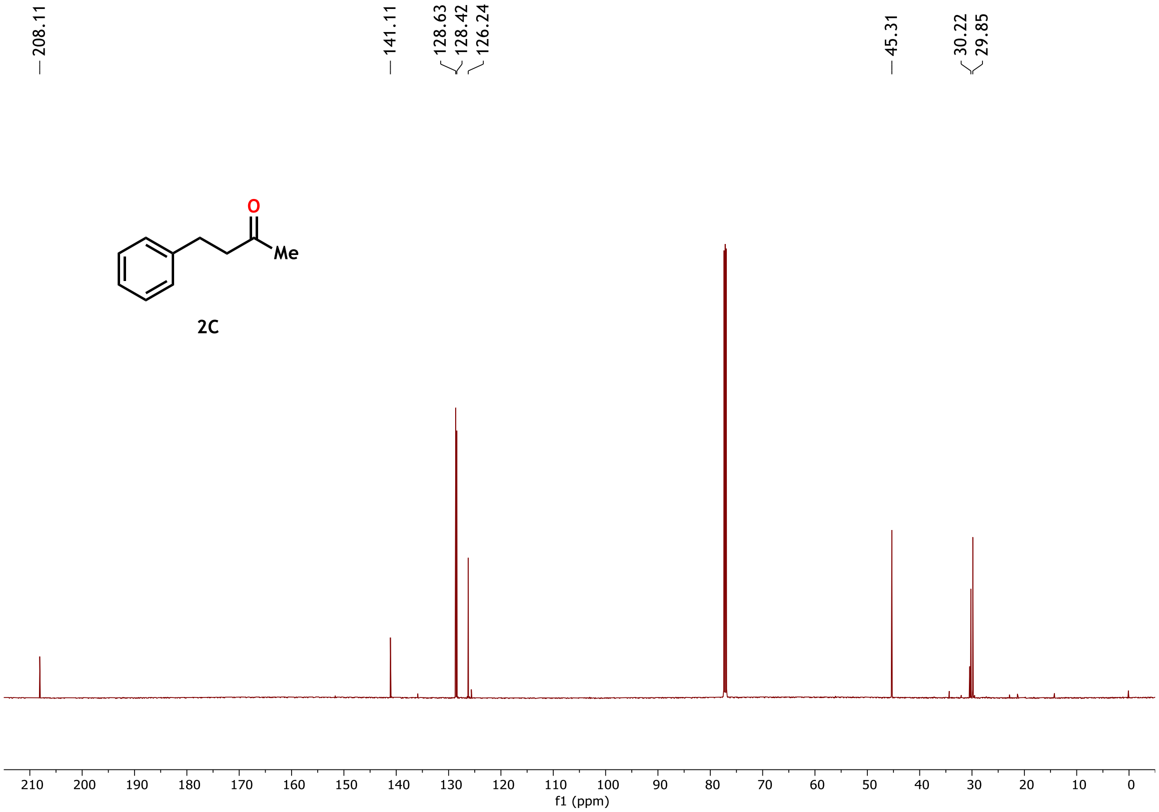

Figure S51. ^{13}C NMR (151 MHz, $CDCl_3$) of 4-phenylbutan-2-one (**2C**)

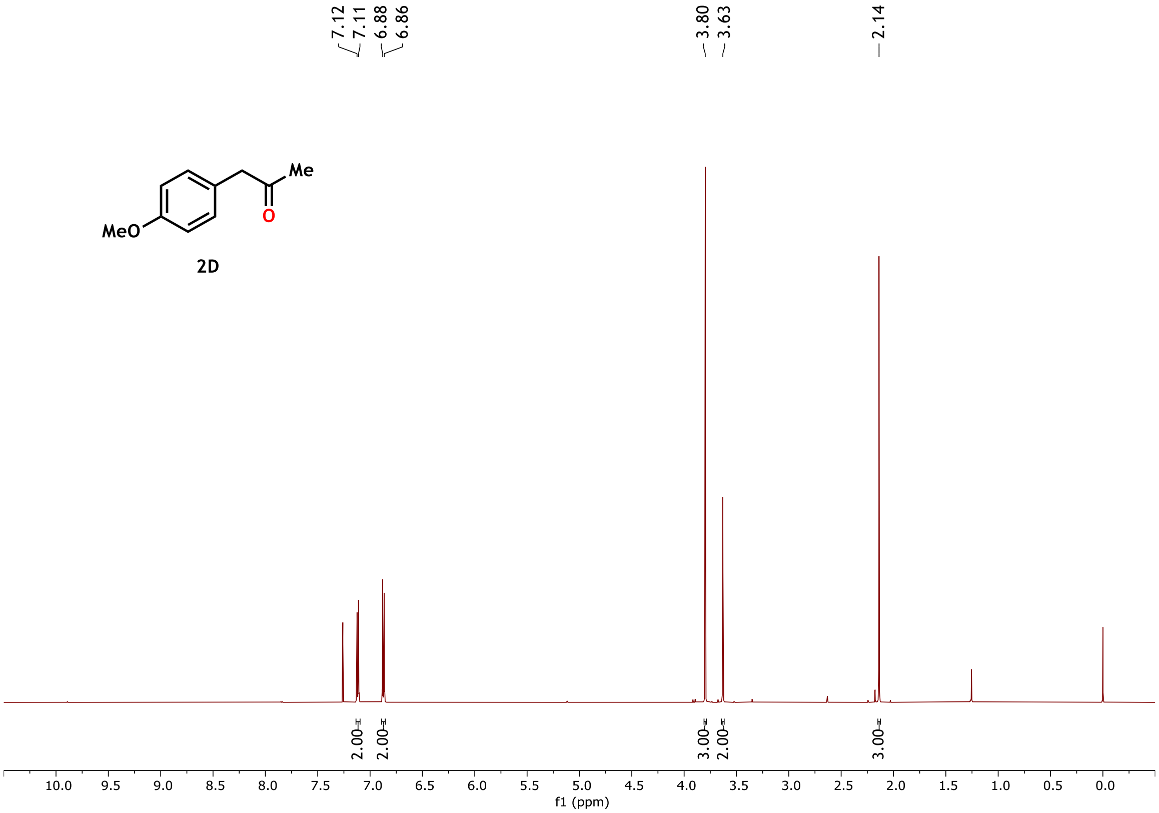

Figure S52. ^{1}H NMR (600 MHz, $CDCl_3$) of 1-(4-methoxyphenyl)propan-2-one (**2D**)

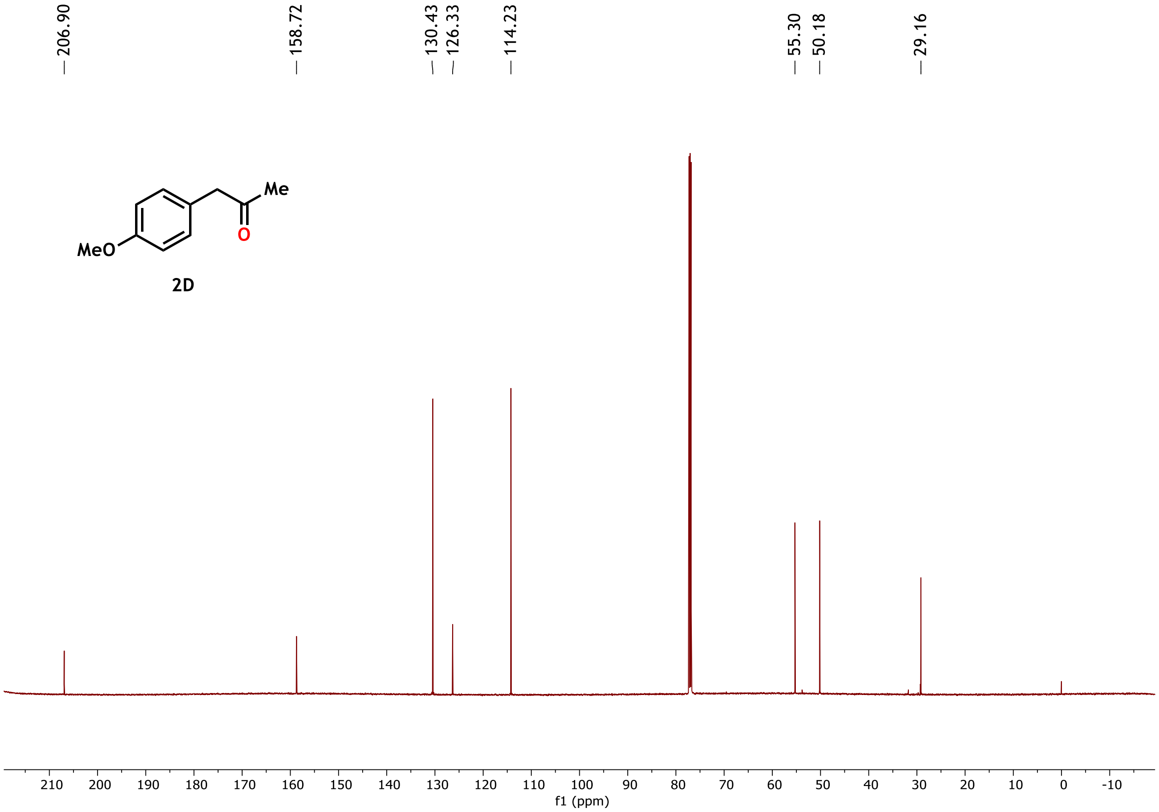

Figure S53. ^{13}C NMR (151 MHz, $CDCl_3$) of 1-(4-methoxyphenyl)propan-2-one (**2D**)

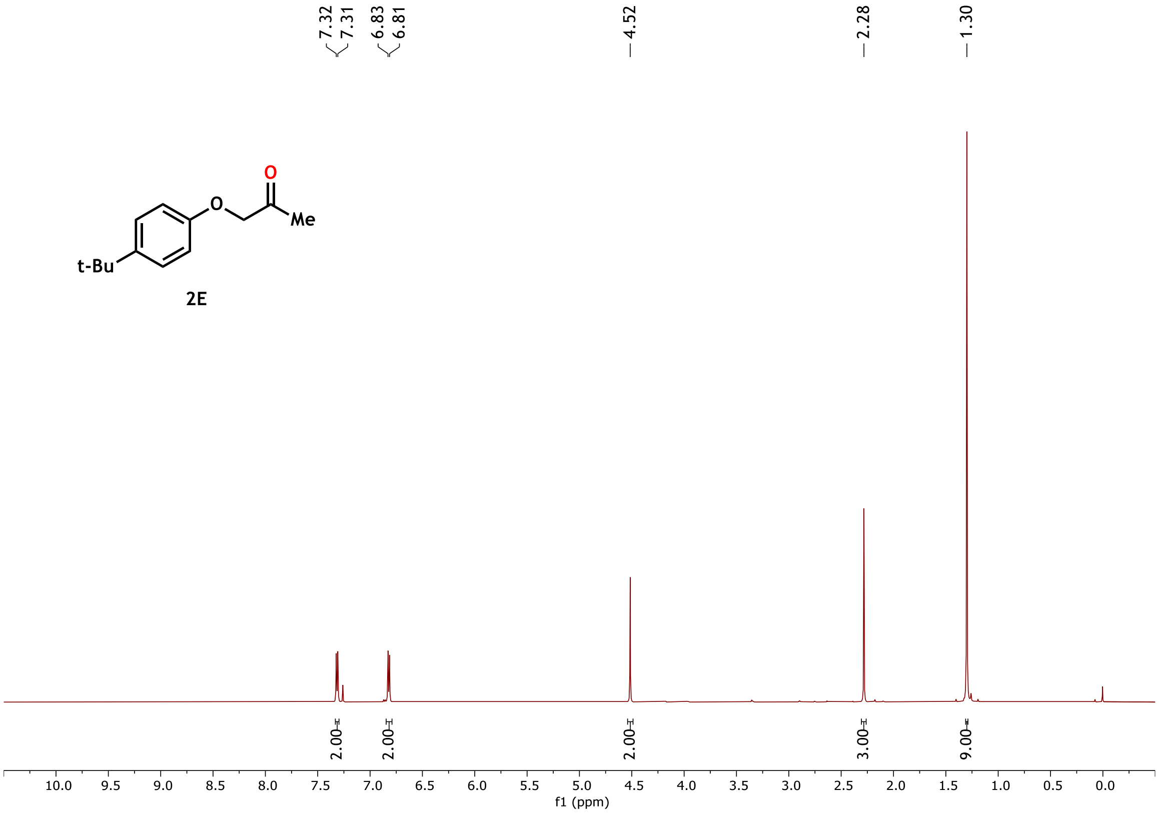

Figure S54. ^{1}H NMR (600 MHz, $CDCl_3$) of 1-(4-(tert-butyl)phenoxy)propan-2-one (**2E**)

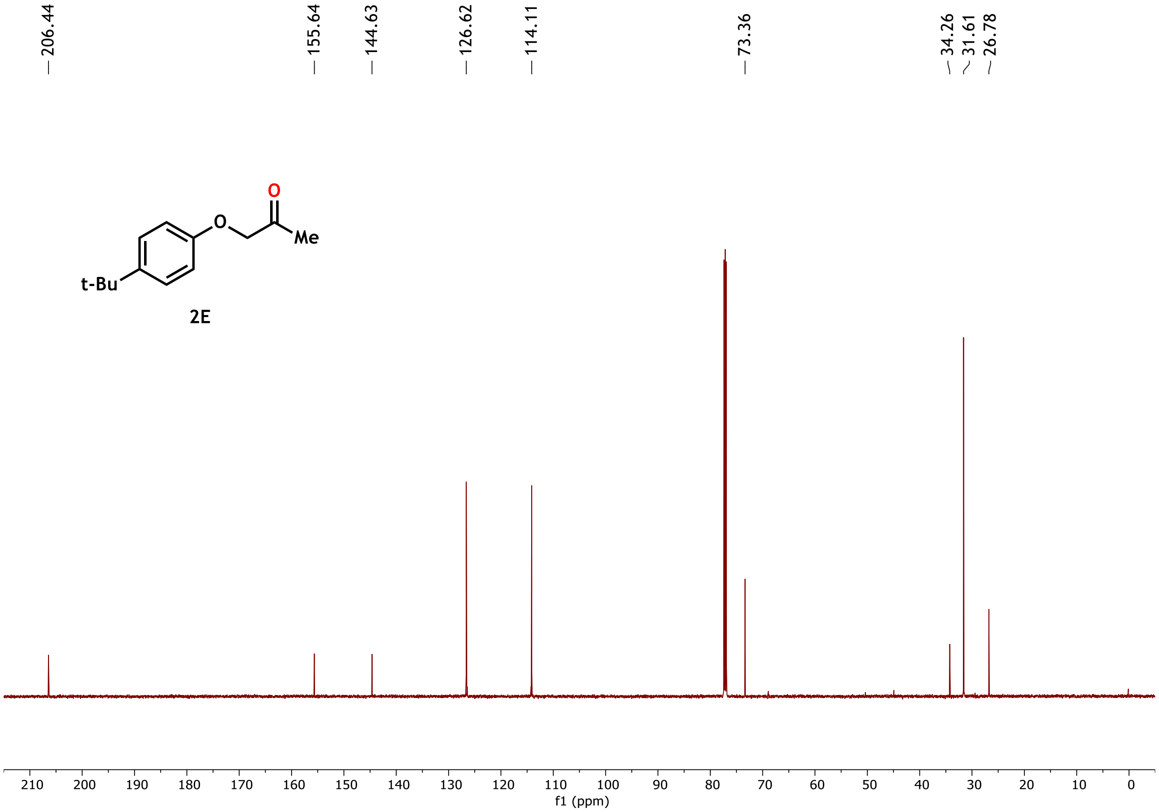

Figure S55. ^{13}C NMR (151 MHz, $CDCl_3$) of 1-(4-(tert-butyl)phenoxy)propan-2-one (**2E**)

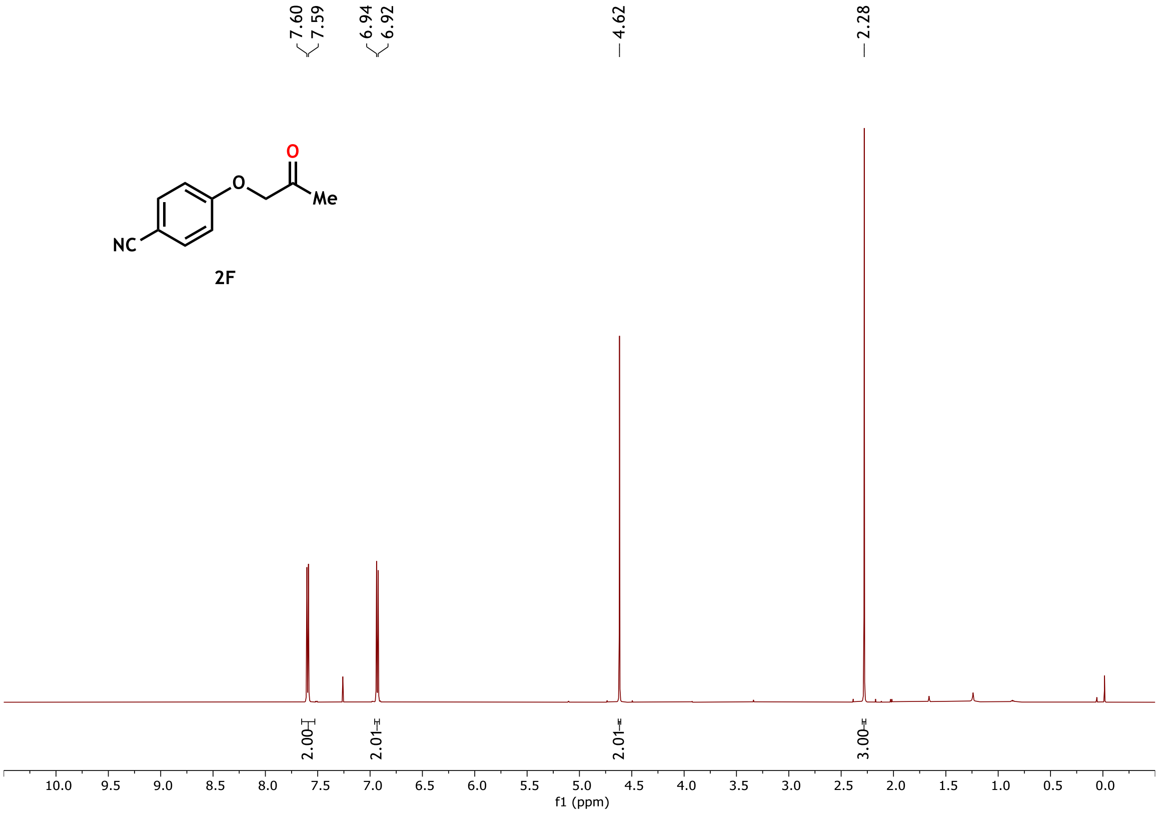

Figure S56. ^{1}H NMR (600 MHz, $CDCl_3$) of 4-(2-oxopropoxy)benzonitrile (**2F**)

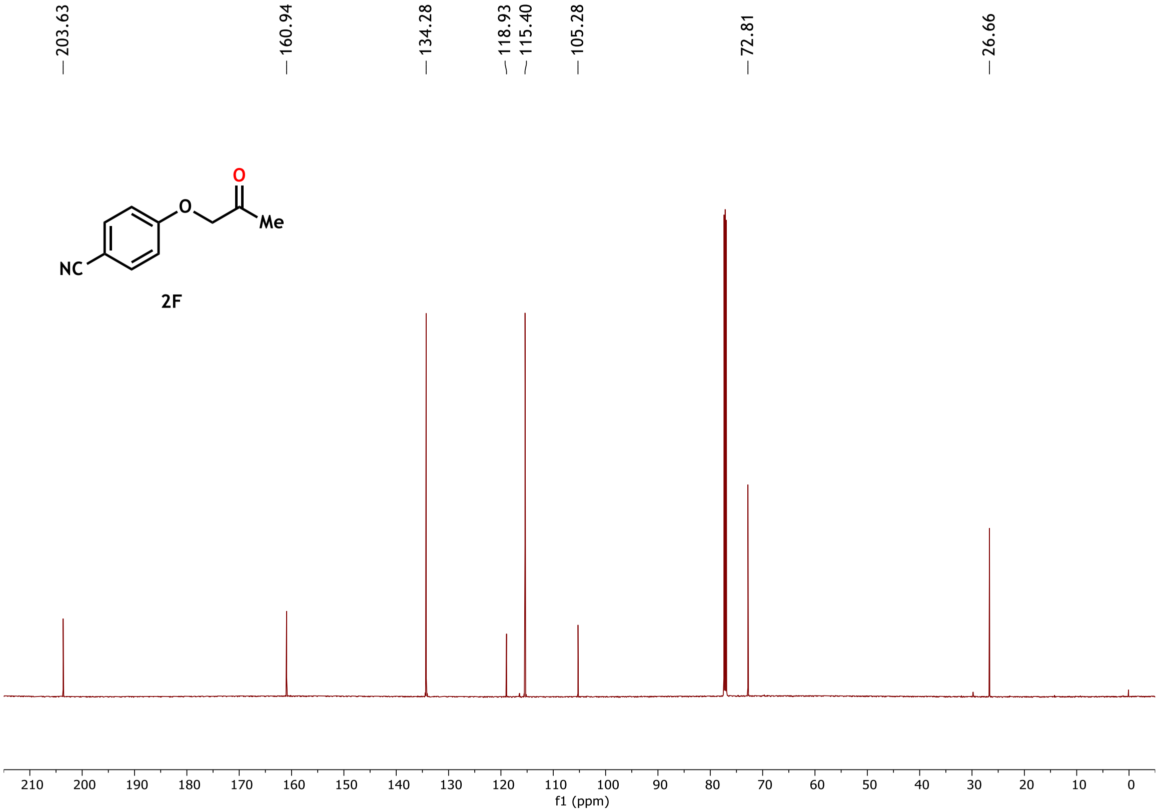

Figure S57. ^{13}C NMR (151 MHz, $CDCl_3$) of 4-(2-oxopropoxy)benzonitrile (**2F**)

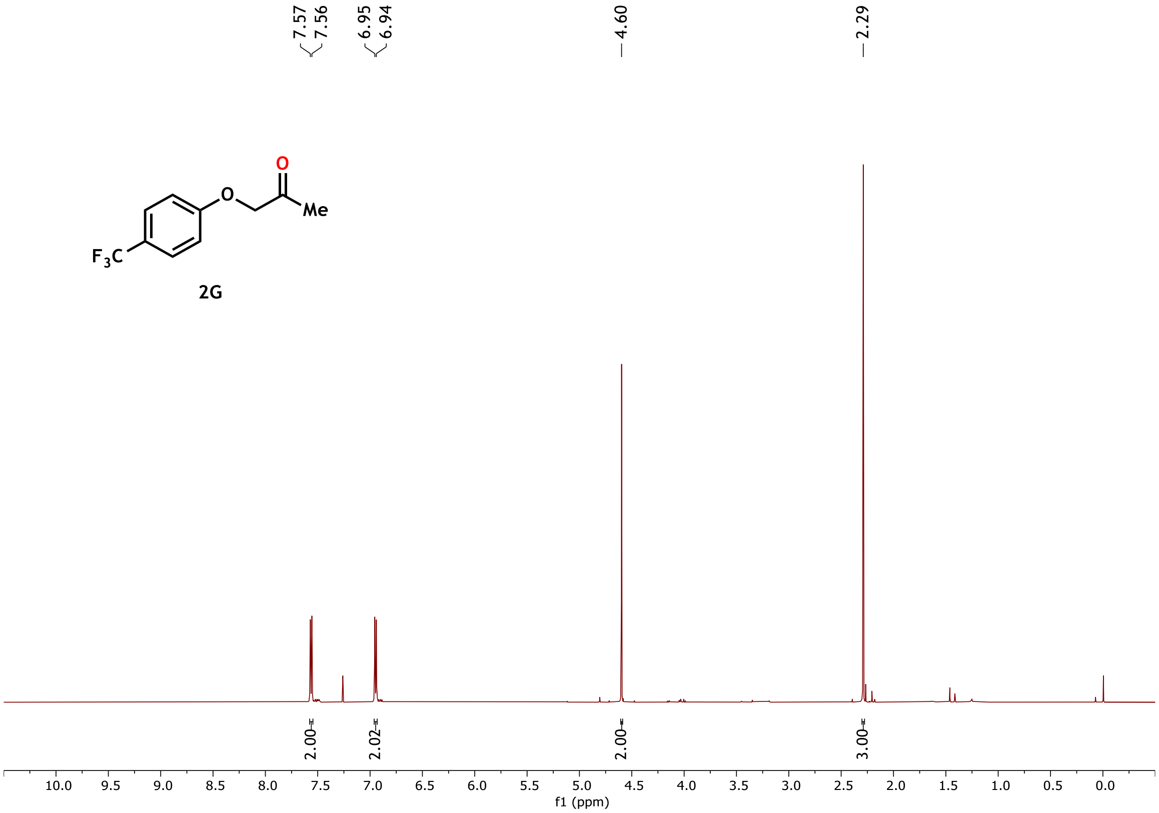

Figure S58. ^{1}H NMR (600 MHz, $CDCl_3$) of 1-(4-(trifluoromethyl)phenoxy)propan-2-one (**2G**)

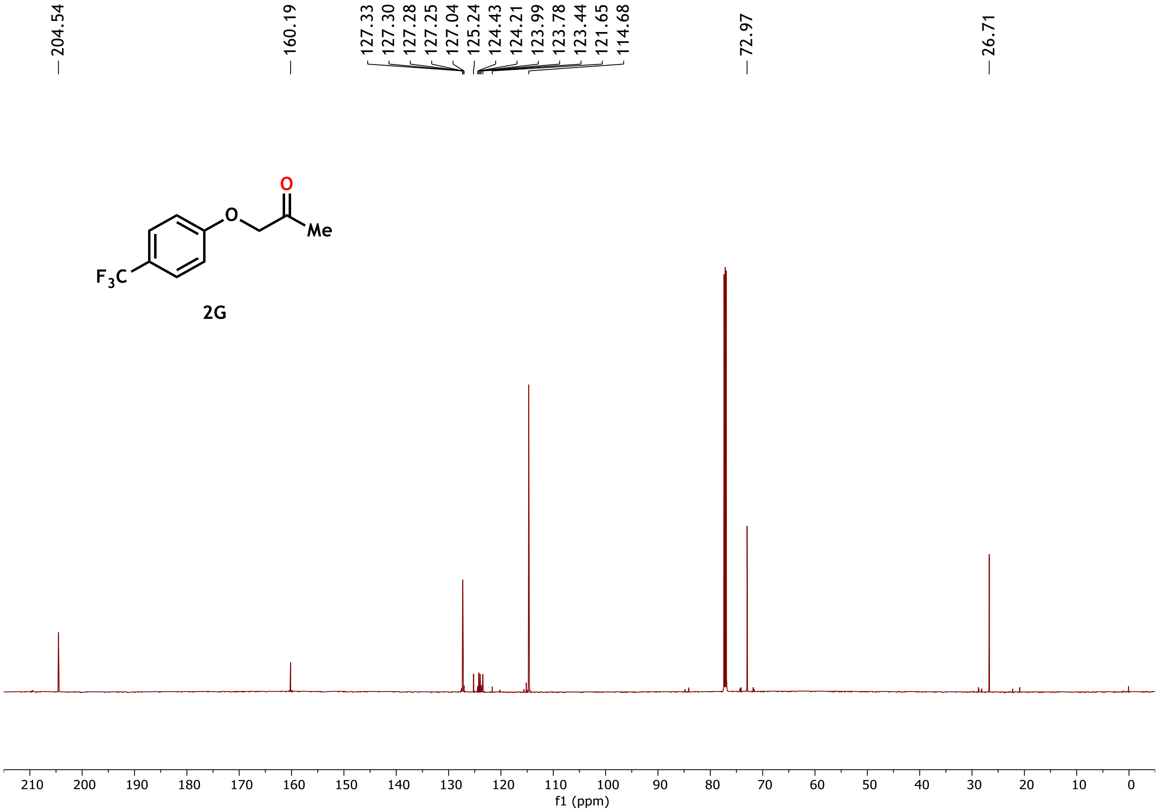

Figure S59. ^{13}C NMR (151 MHz, $CDCl_3$) of 1-(4-(trifluoromethyl)phenoxy)propan-2-one (**2G**)

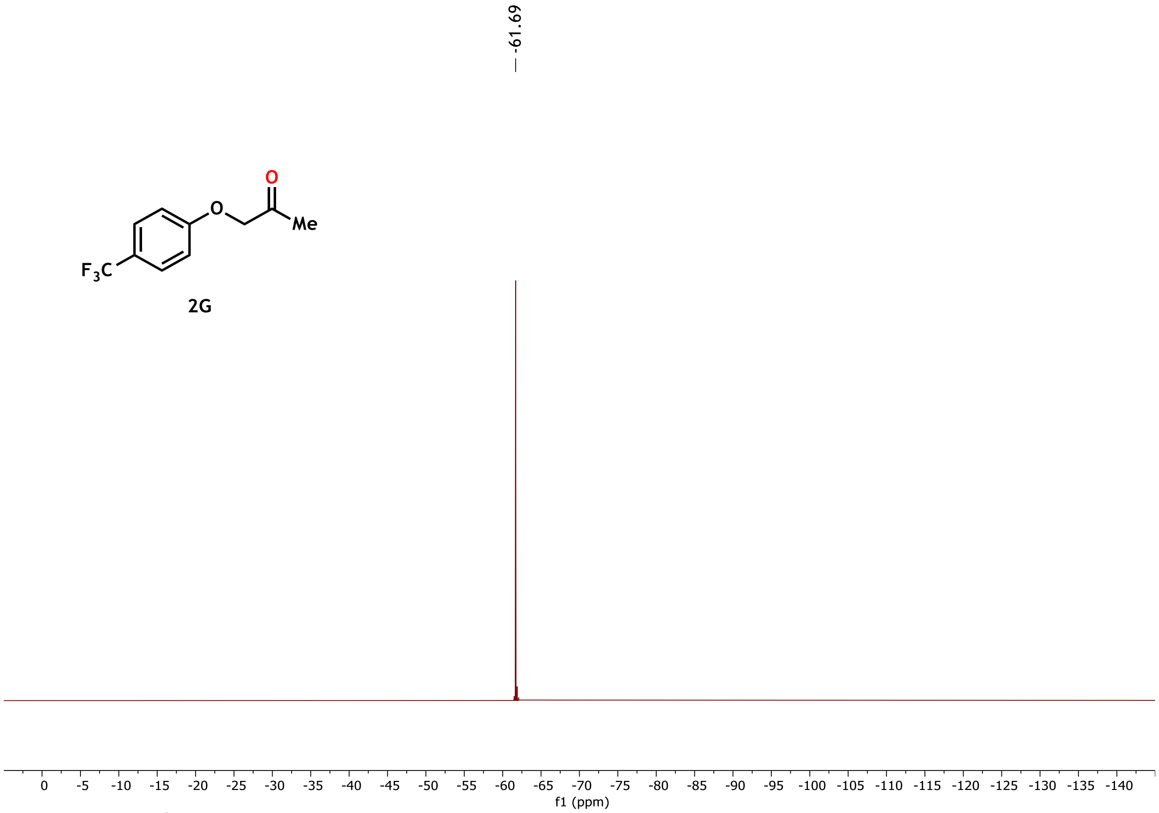

Figure S60. ^{19}F NMR (594 MHz, $CDCl_3$) of 1-(4-(trifluoromethyl)phenoxy)propan-2-one (**2G**)

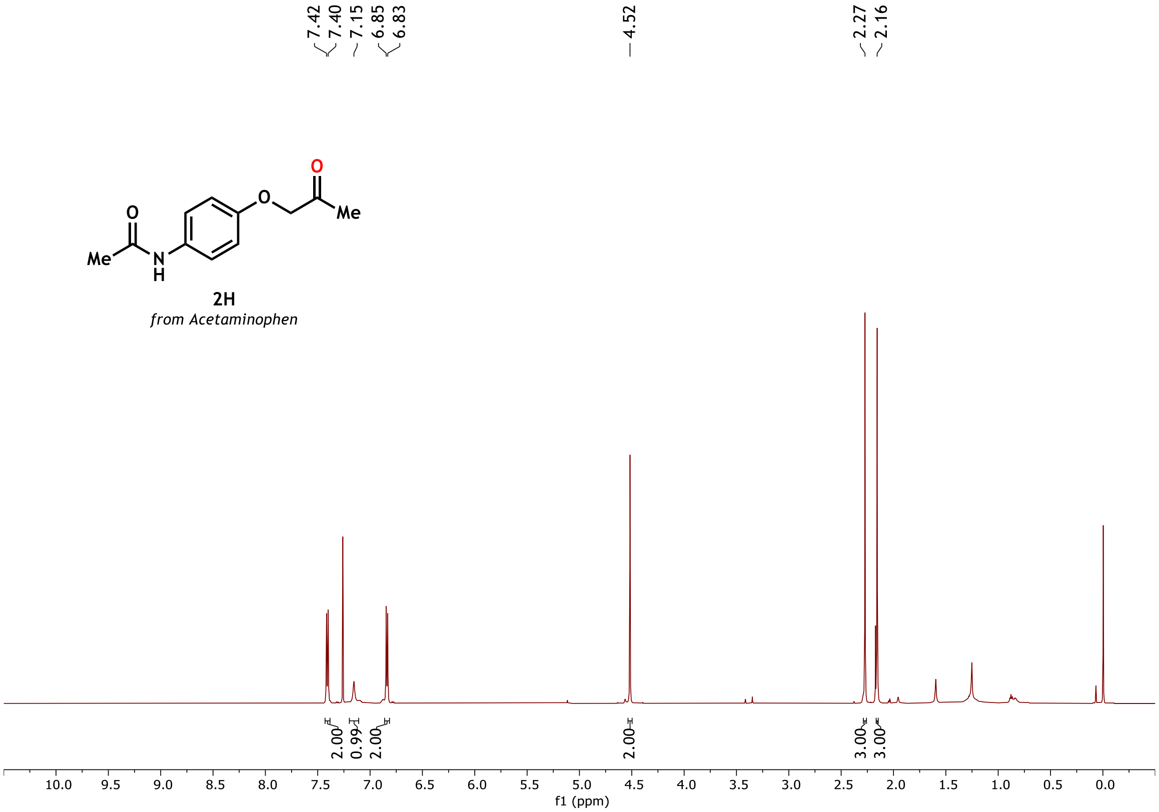

Figure S61. ^{1}H NMR (600 MHz, $CDCl_3$) of N-(4-(2-oxopropoxy)phenyl)acetamide (**2H**)

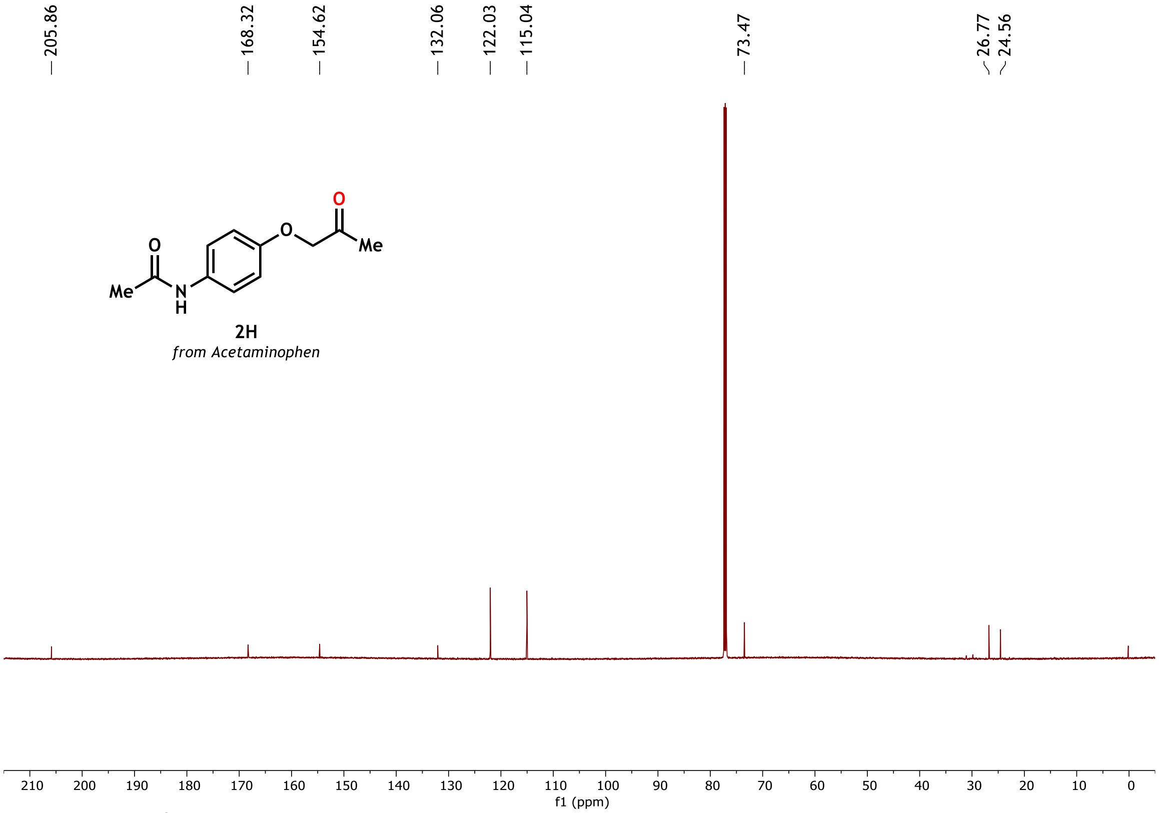

Figure S62. ^{13}C NMR (151 MHz, $CDCl_3$) of N-(4-(2-oxopropoxy)phenyl)acetamide (**2H**)

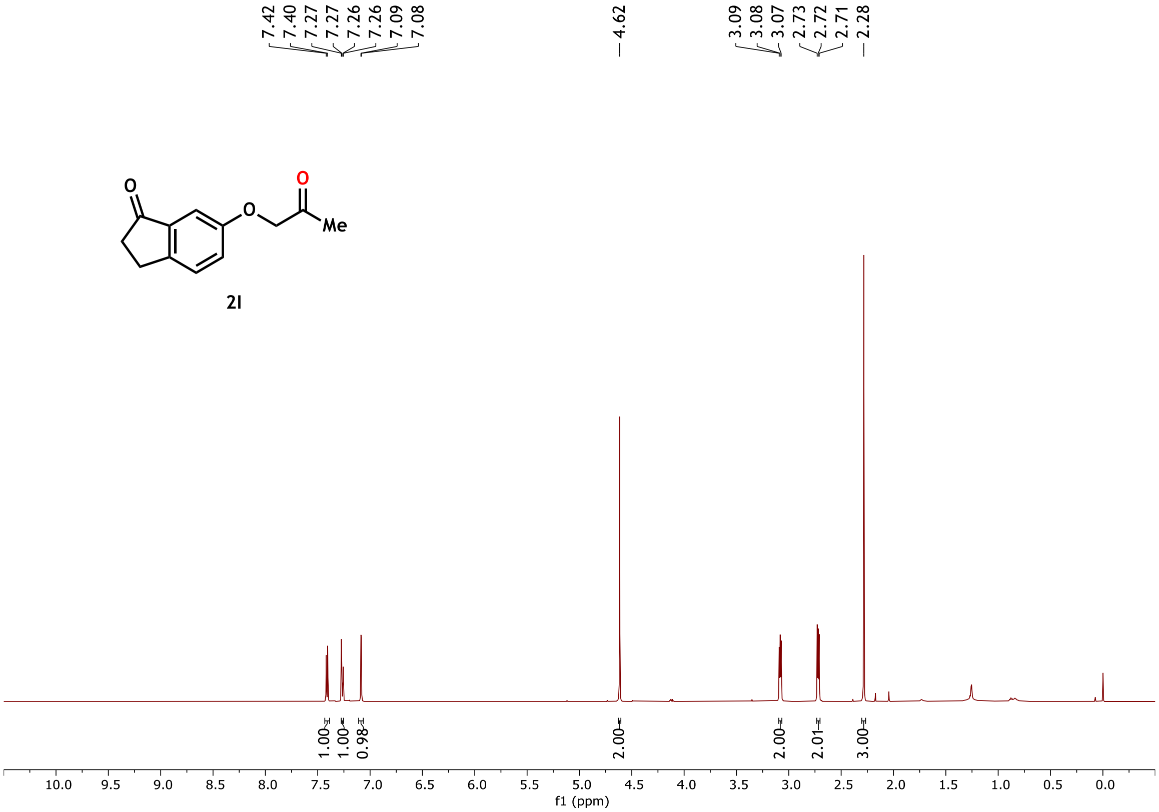

Figure S63. ^{1}H NMR (600 MHz, $CDCl_3$) of 6-(2-oxopropoxy)-2,3-dihydro-1H-inden-1-one (**2l**)

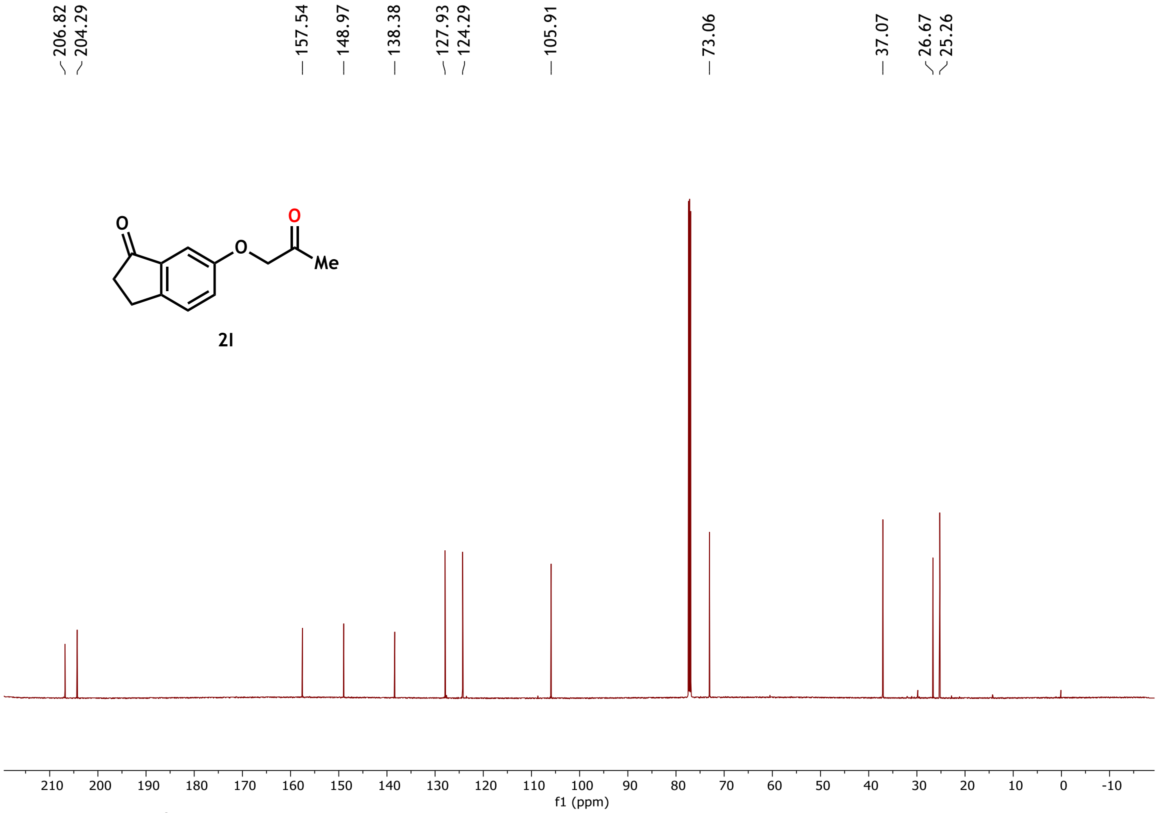

Figure S64. ^{13}C NMR (151 MHz, $CDCl_3$) of 6-(2-oxopropoxy)-2,3-dihydro-1H-inden-1-one (**2l**)

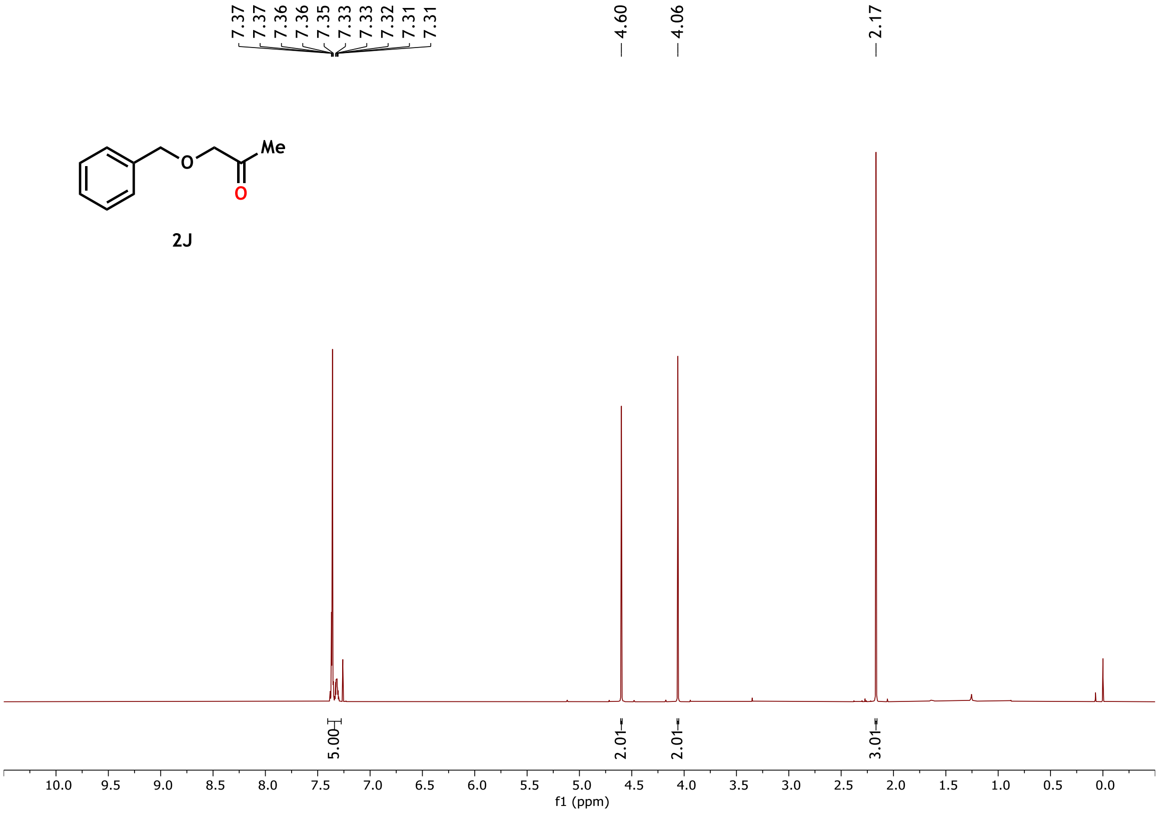

Figure S65. ^{1}H NMR (600 MHz, $CDCl_3$) of 1-(benzyloxy)propan-2-one (**2J**)

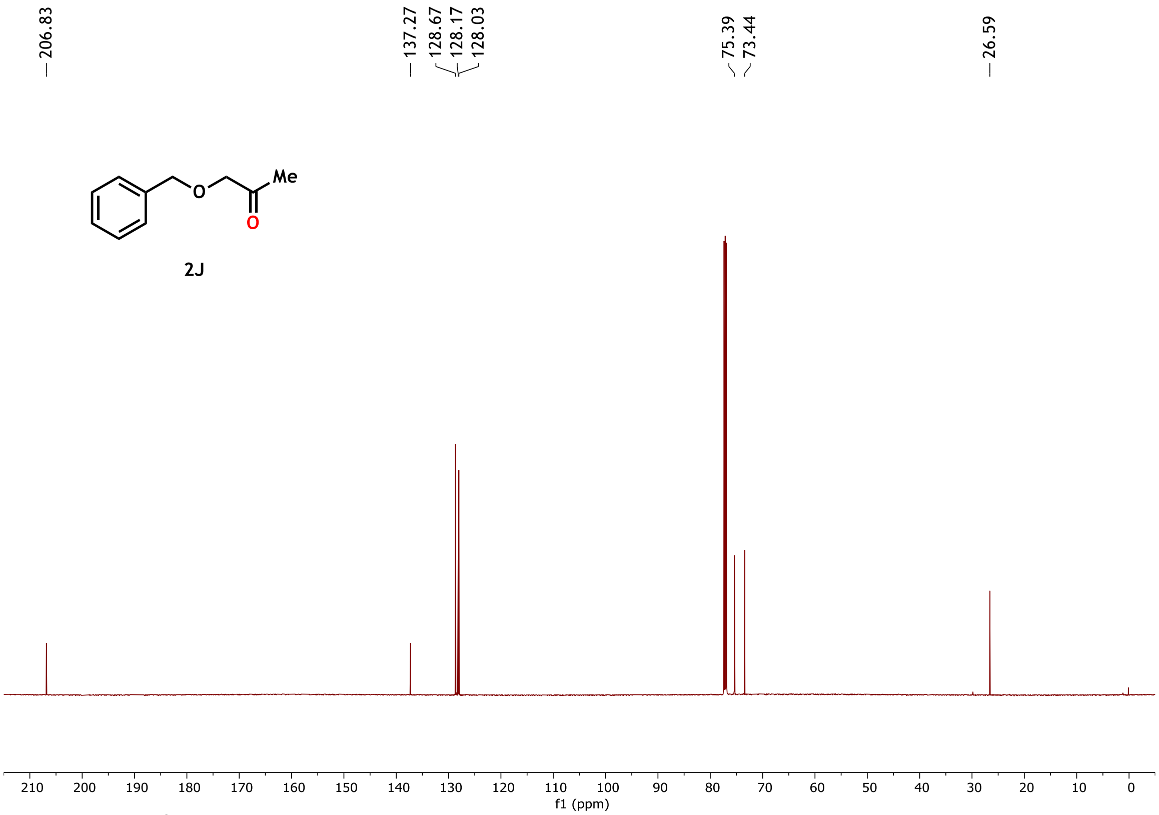

Figure S66. ^{13}C NMR (151 MHz, $CDCl_3$) of 1-(benzyloxy)propan-2-one (**2J**)

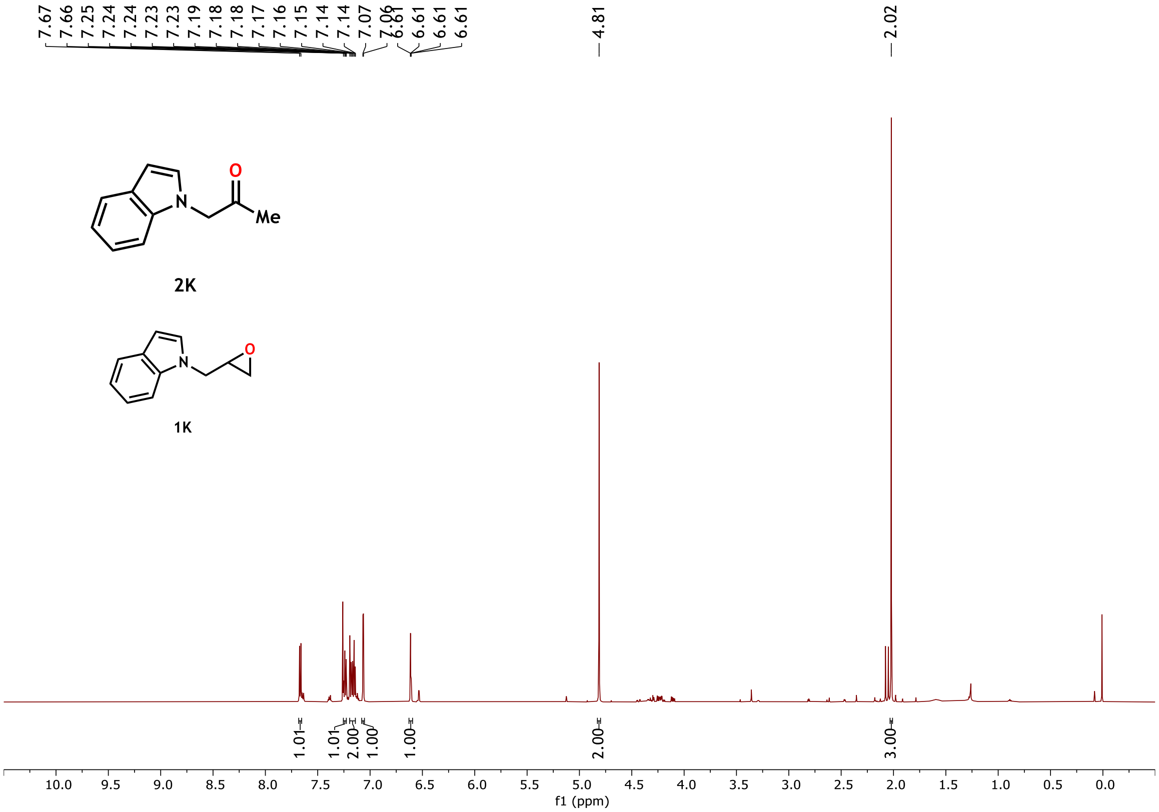

Figure S67. ^{1}H NMR (600 MHz, $CDCl_3$) of 1-(1H-indol-1-yl)propan-2-one (**2K**)

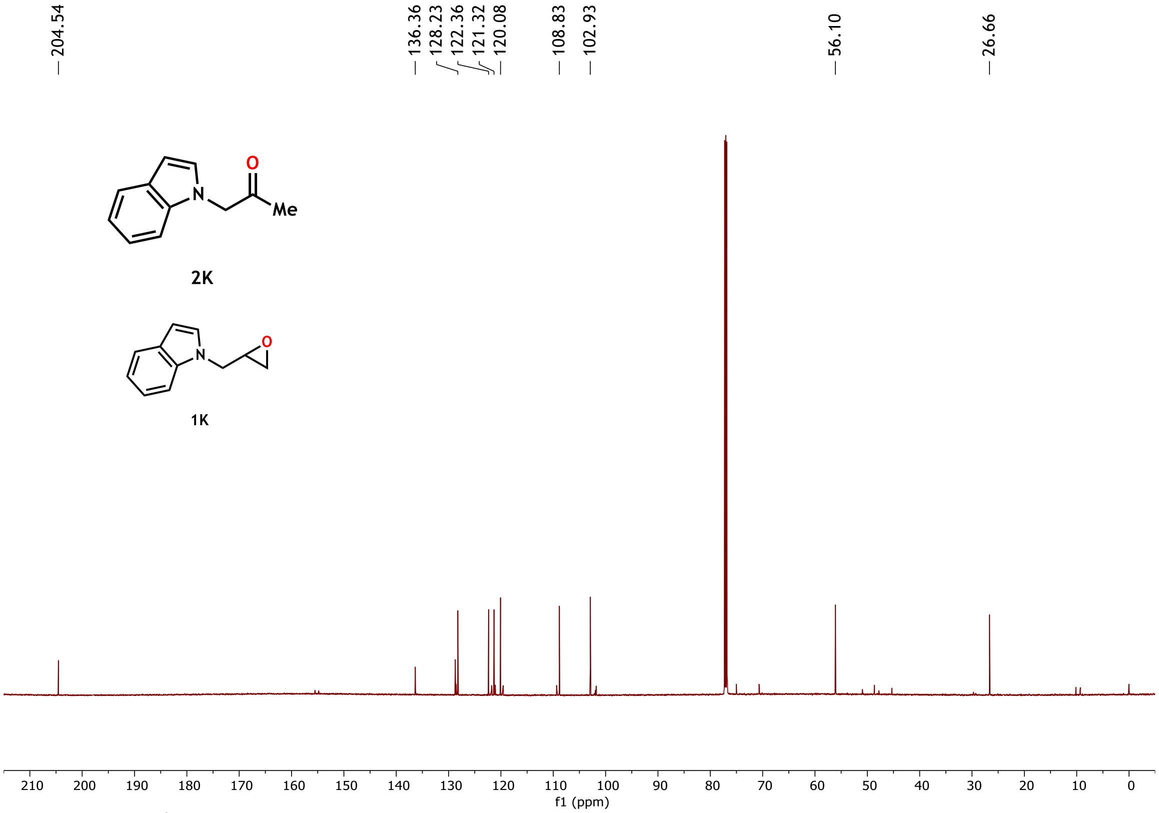

Figure S68. ^{13}C NMR (151 MHz, $CDCl_3$) of 1-(1H-indol-1-yl)propan-2-one (**2K**)

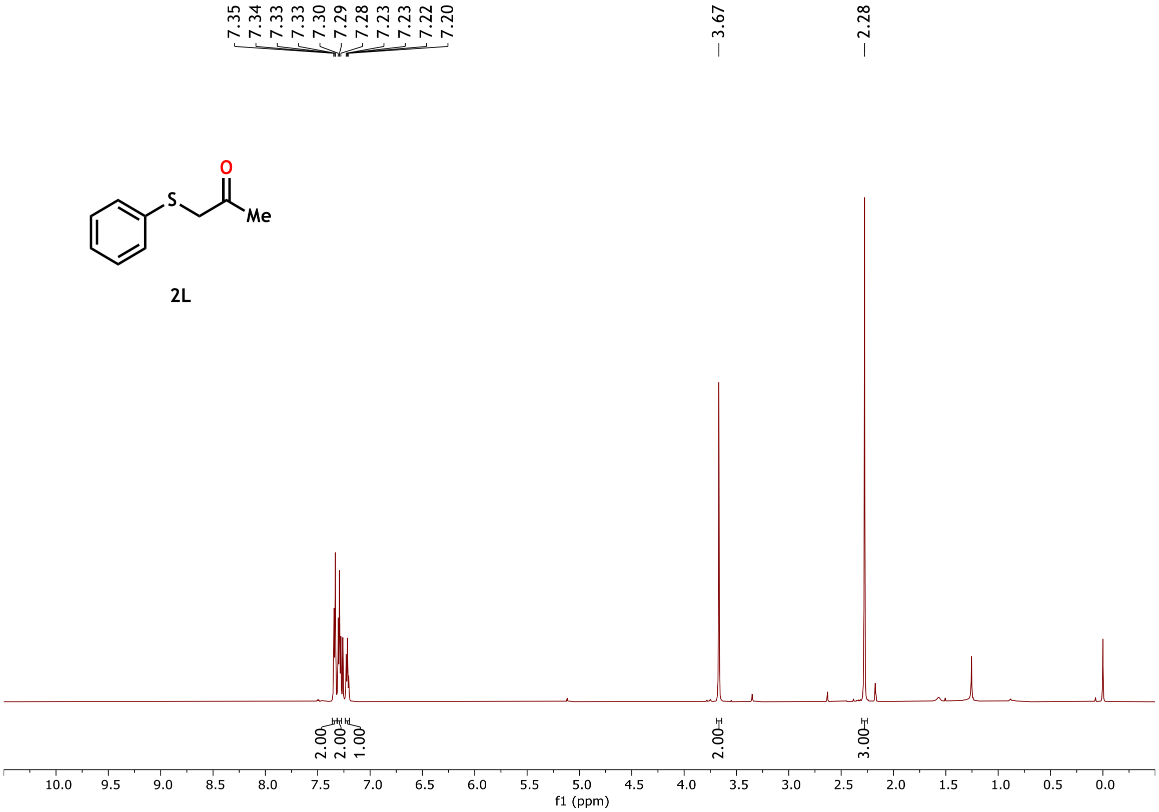

Figure S69. ^{1}H NMR (600 MHz, $CDCl_3$) of 1-(phenylthio)propan-2-one (**2L**)

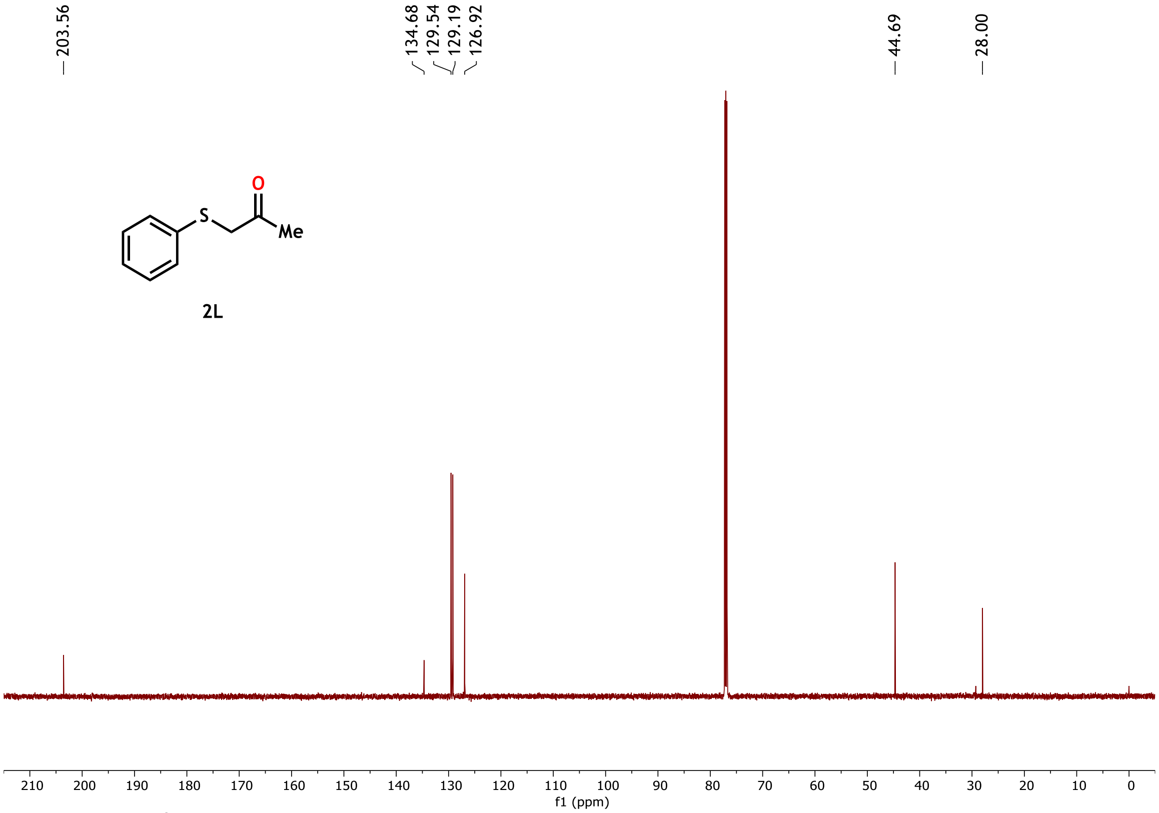

Figure S70. ^{13}C NMR (151 MHz, $CDCl_3$) of 1-(phenylthio)propan-2-one (**2L**)

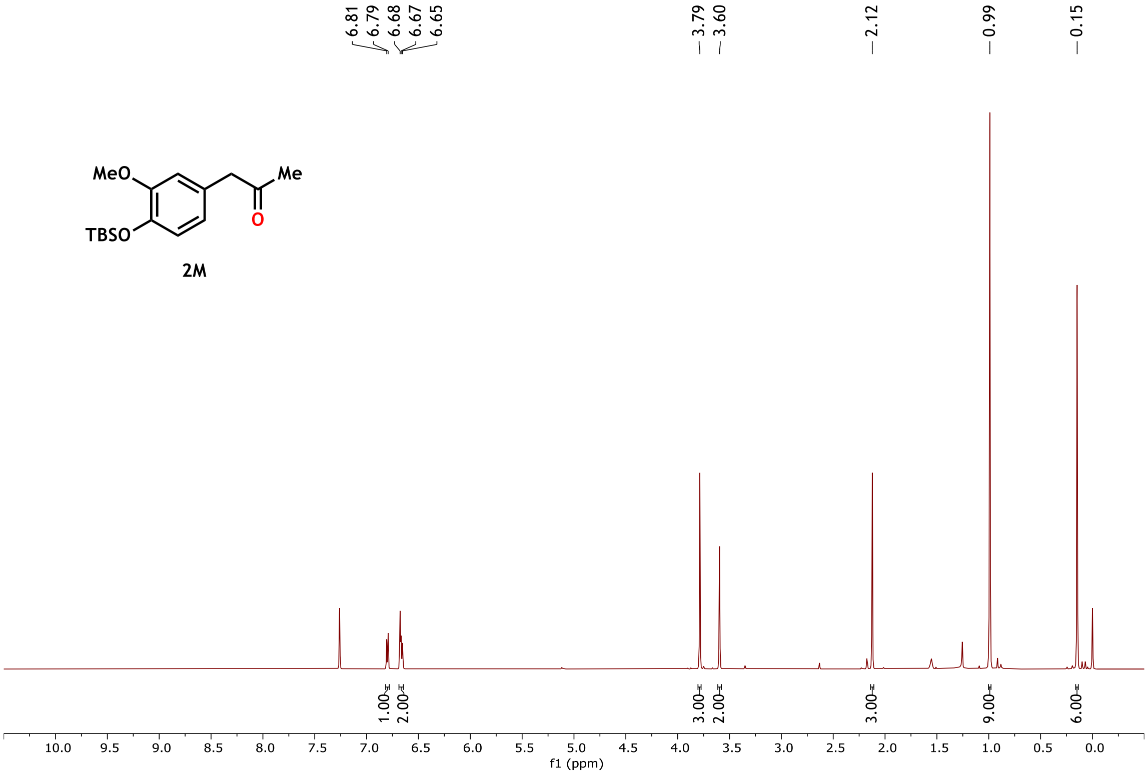

Figure S71. ^{1}H NMR (600 MHz, $CDCl_3$) of 1-(4-((tert-butyldimethylsilyl)oxy)-3-methoxyphenyl)propan-2-one (**2M**)

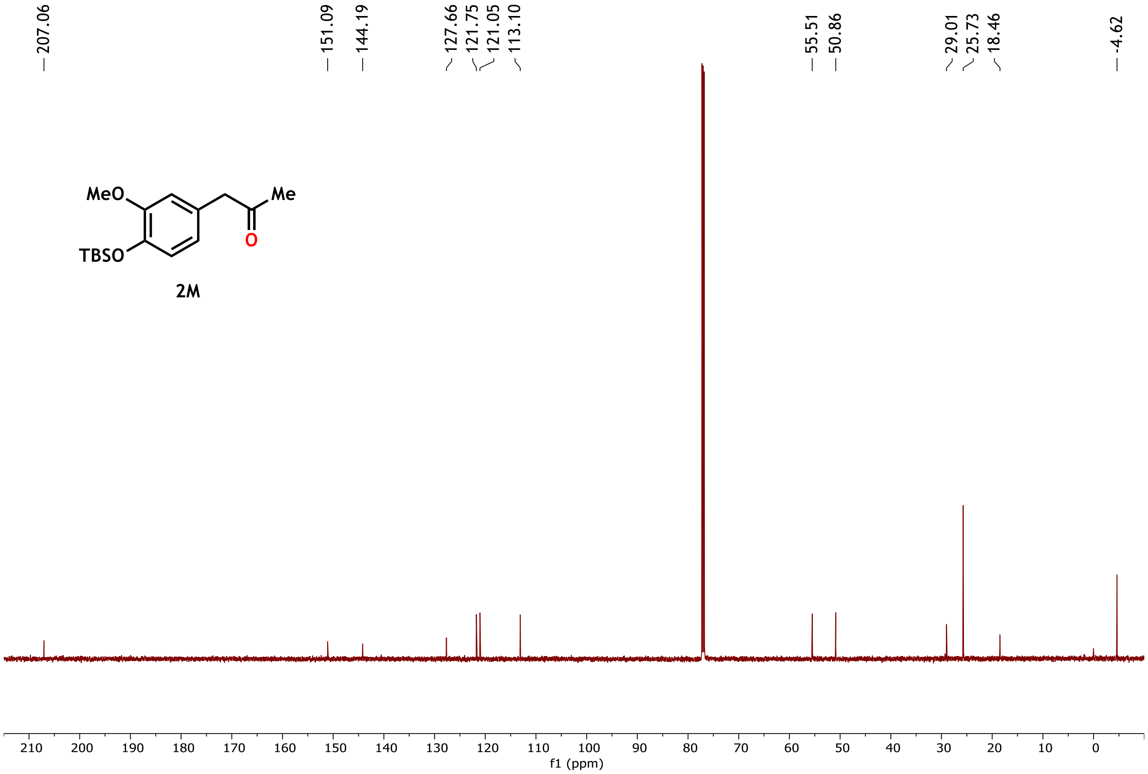

Figure S72. ^{13}C NMR (151 MHz, $CDCl_3$) of 1-(4-((tert-butyldimethylsilyl)oxy)-3-methoxyphenyl)propan-2-one (**2M**)

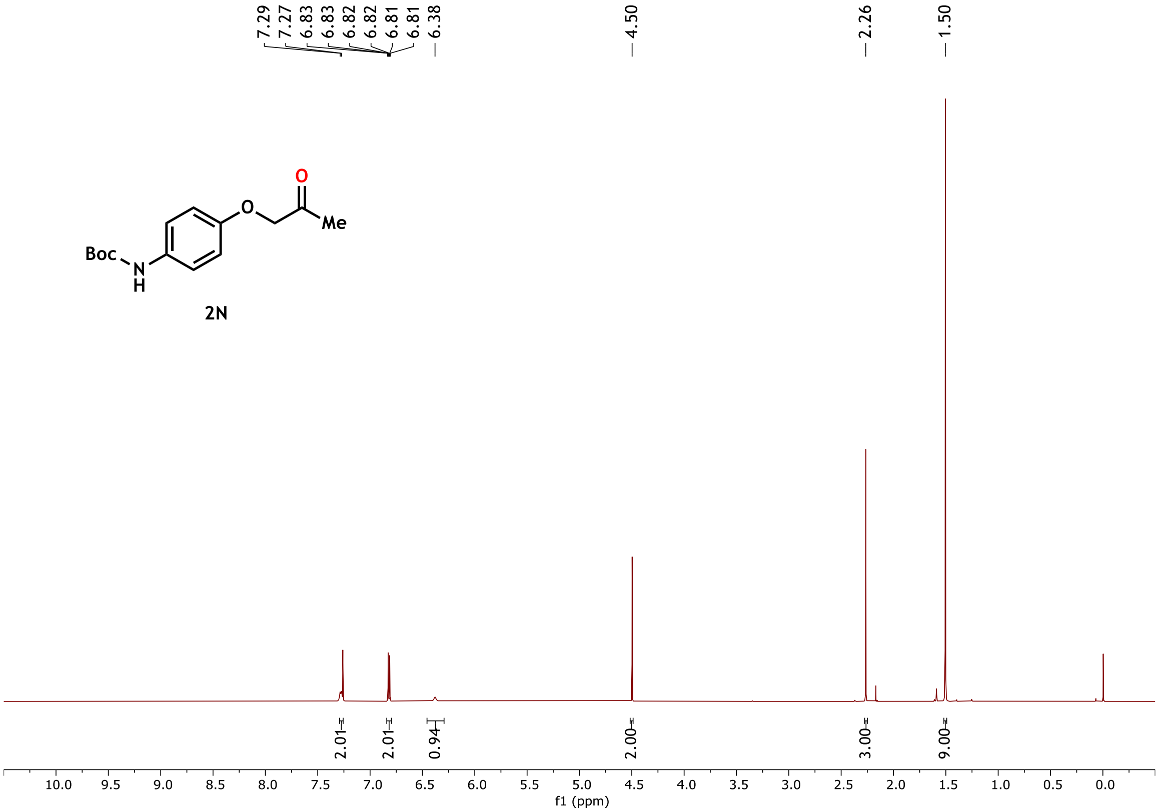

Figure S73. ^{1}H NMR (600 MHz, $CDCl_3$) of tert-butyl (4-(2-oxopropoxy)phenyl)carbamate (**2N**)

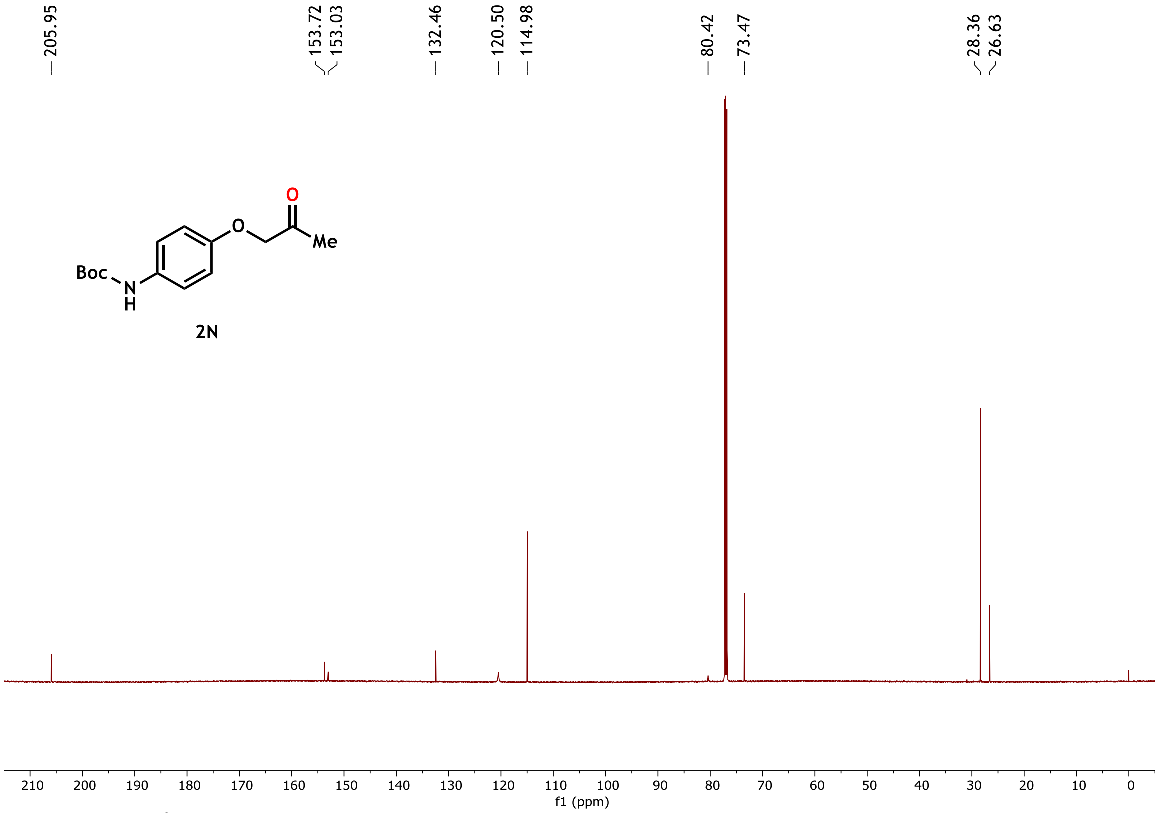

Figure S74. ^{13}C NMR (151 MHz, $CDCl_3$) of tert-butyl (4-(2-oxopropoxy)phenyl)carbamate (**2N**)
